# Supplementary material for: Home-based rehabilitation programme compared with traditional physiotherapy for patients at risk of poor outcome after knee arthroplasty: the CORKA randomised controlled trial
Source: BMJ Open. 2021 Aug 27;11(8):e052598. doi: 10.1136/bmjopen-2021-052598 (PMC8404435; doi:10.1136/bmjopen-2021-052598)
Supplement: Supplementary data [file bmjopen-2021-052598supp001.pdf]

Corka

CORKA Screening Tool

Study code:	Site ID code:	Participants Study Number*:	Initials:
C K –	–		

* Add in once Participant has been randomised

DOB: _ _/_ _ _/_ _ _ _ **Principal Investigator:** _______________ Date _ _/_ _ _/_ _ _ _

Participant Screening Number (number given when participant originally screened) CSN __ __ __ __

From pre-admission clinic, complete the following:

	**SCORE** *(Please Circle)*
1. What is the participant's Body Mass Index (BMI)?	
Normal weight (<25kg/m^2)	**0**
Overweight (25-29.9kg/m^2)	**1**
Obese (≥30kg/m^2)	**2**
2. From Q1 of the Oxford Knee Score (OKS) questionnaire; *'How would you describe the pain you usually have from your knee?'*	
None OR very mild OR mild	**0**
Moderate OR severe	**4**
3. What is the participant's health status?	
Fit and healthy with mild systemic disease i.e. controlled hypertension/diabetes	**0**
Severe systemic disease i.e. unstable coronary heart disease	**2**
4. In the **past four weeks** have you been limited in the kind of work you do or other regular activities you carry out as a result of feeling anxious or depressed?	
None of the time OR a little of the time OR some of the time	**0**
Most of the time OR all of the time	**2**
**TOTAL:**	

For patients with a score of 5 or more, continue to screen for eligibility onto the study.

For patients with a score below 5, stop the screening process and record this in the screening log.

Barker KL, *et al. BMJ Open* 2021; 11:e052598. doi: 10.1136/bmjopen-2021-052598
