# Supplementary material for: Home-based rehabilitation programme compared with traditional physiotherapy for patients at risk of poor outcome after knee arthroplasty: the CORKA randomised controlled trial
Source: BMJ Open. 2021 Aug 27;11(8):e052598. doi: 10.1136/bmjopen-2021-052598 (PMC8404435; doi:10.1136/bmjopen-2021-052598)
Supplement: Supplementary data [file bmjopen-2021-052598supp002.pdf]

Appendix B – content of treatment Interventions

The CORKA home-based intervention was a multicomponent rehabilitation programme delivered to participants in their own home by a mixture of qualified staff and rehabilitation assistants. Its main component was an exercise programme, but it also included functional task practice, adherence approaches and provision of appropriate aids or equipment. It consisted of an initial appointment and 6 follow up sessions.

Initial appointment – assessment

The subjective assessment included their current symptoms and the perceived level of pain or discomfort on a VAS scale from 0-10 with 0 being 'no symptoms' and 10 the 'worse imaginable symptoms', their social history, any medication taken, their history of falls and their activities of daily living (ADLs).

The objective assessment documented the participant's wound, the range of movement in the operated and non-operated knees, strength of quadriceps, hamstrings, hip abductors and the calf muscles, scored on an Oxford grading scale. Standing balance was assessed by observing and recording the ability to stand with feet side by side, or in a semi-tandem or tandem stance. An assessment was made of gait and if appropriate ability to ascend and descend stairs. If deemed necessary and safe any transfers were assessed.

Finally, observation of at least one functional task took place which was identified by the participant as being potentially problematic. The exercise programme was subsequently tailored to meet the specific needs, goals and functional problems of the individual.

Initial appointment – exercise programme

Following the assessment the physiotherapist and rehabilitation assistant set each participant an exercise programme, which would replace any post-operative programme given on discharge from hospital. It would consist of exercises categorised into the following sections :

1. Knee range of motion - flexion
2. Knee range of motion – extension
3. Basic quadriceps strengthening
4. Strengthening exercises – quadriceps
5. Strengthening exercises – hamstrings
6. Strengthening exercises – hip abductors
7. Strengthening exercises – calf
8. Static balance exercises
9. Dynamic balance and walking skills exercises

The selection of each specific exercise took place as follows, for ROM exercises the physiotherapist selected an exercise and then checked that they and the participant were happy with how it was performed. If this was the case the participant was asked to perform 3 sets of 10-15 repetitions, unless a stretch had been selected, when they were asked to undertake 3 sets of 3-5 repetitions with a 30 to 60 second hold.

For strengthening exercises the participant was given an exercise that they could perform for 6-12 repetitions with good form at a moderate intensity. Perceived intensity was measured using the 10 point Rating of Perceived Exertion (RPE) scale (Borg 1998). The participant was given an exercise

where they feel they were working at RPE of 3-4 (moderate to somewhat hard). Participants were asked to complete 1 to 3 sets of 6-12 reps of strengthening exercises.

For the balance section, participants were prescribed an exercise that was sufficiently challenging without putting the participant at risk of falling. Participants were asked to perform 3 repetitions of the exercise with a 30 second hold.

For the final section, gait skills there were a variety of exercises to choose from. The physiotherapist was asked to prescribe an exercise that was sufficiently challenging without putting the participants at risk of falling. The exact number of sets and reps were determined based on the particular exercise selected and the individual patient.

During session 3 a graduated walking programme was introduced. Participants were asked about the maximum time or distance that they have managed to walk since surgery and were encouraged to increase this distance or time gradually in small increments.

Participants were also asked to practice between 1-3 specific functional tasks highlighted in the initial assessment.

CORKA intervention exercise prescription time points

	Range of Movement	Basic Quads strength	Strengthening	Balance	Gait Skills	Task practice	Walking programme
Session 1	✓	✓	✓	✓	✓	✓	
Session 2	✗	✗	⇧	⇧	⇧	⇧	
Session 3	✗	✗	⇧	⇧	⇧	⇧	✓
Session 4	✗	✗	⇧	⇧	⇧	⇧	⇧
Session 5	✗	✗	⇧	⇧	⇧	⇧	⇧
Session 6	✗	✗	⇧	⇧	⇧	⇧	⇧
Session 7	✗	✗	⇧	⇧	⇧	⇧	⇧

✓ = issue exercise

✗ = Stop exercise once appropriate level of function reached

⇧ = Progress exercise

CORKA Intervention Exercises Table

Exercise	Level	Purpose	Functional relevance
Hip and knee flexion 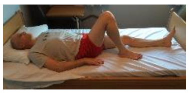	1A	Lower limb range of movement	Walking, stair climbing, kneeling
Passive knee flexion 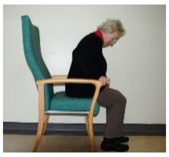	1B	Lower limb range of movement	Walking, stair climbing, sitting and standing up from a chair, kneeling
Active assisted knee flexion 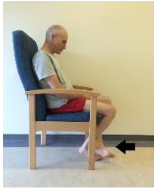	1C	Lower limb range of movement	Walking, stair climbing, sitting and standing up from a chair, kneeling
Hold-relax knee flexion 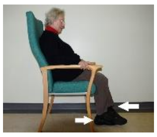	1D	Lower limb range of movement	Walking, stair climbing, sitting and standing up from a chair, kneeling
Active Knee flexion 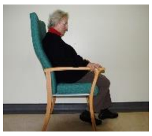	1E	Lower limb range of movement	Walking, stair climbing, sitting and standing up from a chair, kneeling
Hip and knee flexion with overpressure 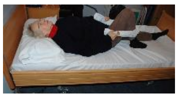	1F	Lower limb range of movement	Walking, stair climbing, kneeling
Knee extension stretch 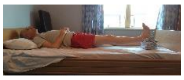	2A	Lower limb range of movement	Walking, stair climbing, standing up from a chair

Knee extension with overpressure 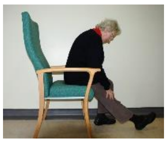	2B	Lower limb range of movement	Walking, stair climbing, standing up from a chair
Knee extension 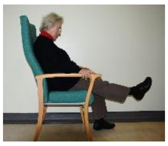	2C	Lower limb range of movement	Walking, stair climbing, standing up from a chair, dressing, getting in/out of car
Hamstring stretch 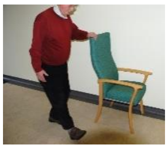	2D	Lower limb range of movement	Walking, stair climbing, dressing
Calf stretch 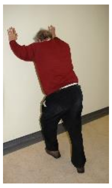	2E	Lower limb range of movement	Walking, stair climbing, dressing
Static Quadriceps 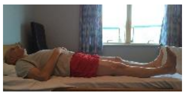	3A	Lower limb strength	Walking, stair climbing, standing up from a chair
Inner range quadriceps 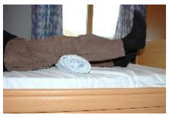	3B	Lower limb strength	Walking, stair climbing, standing up from a chair
Long arc quadriceps 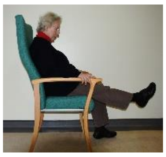	4A	Lower limb strength	Walking, stair climbing, standing up from a chair

Knee extension with ankle weight 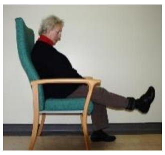	4B	Lower limb strength	Walking, stair climbing, standing up from a chair
Sit to stand using arms 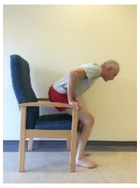	4C	Upper and lower body strength	Walking, stair climbing, standing up from a chair, lifting/carrying household objects, getting in/out of car
Sit to stand without arms 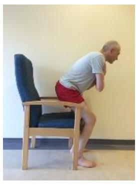	4D	Lower limb and trunk strength	Stair climbing, standing up from a chair, walking, getting in/out of car
Wall slide 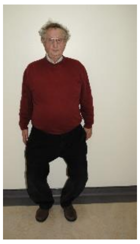	4E	Lower limb strength	Stair climbing, standing up from a chair, walking, getting in/out of car, lifting household objects
Mini Squats 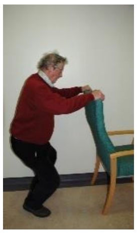	4F	Lower limb strength	Stair climbing, standing up from a chair, walking, getting in/out of car, lifting household objects

Single leg dip 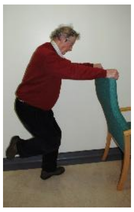	4G	Lower limb strength	Stair climbing, standing up from a chair, walking, lifting household objects
Static hamstrings 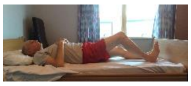	5A	Lower limb strength	Stair climbing, standing up from a chair, walking, lifting household objects
Sliding heel backwards 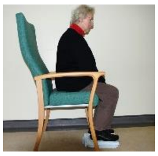	5B	Lower limb strength and range of movement	Stair climbing, standing up from a chair, walking, kneeling
Standing hamstring curls 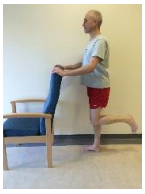	5C	Lower limb strength	Stair climbing, standing up from a chair, walking, lifting household objects
Active hamstrings 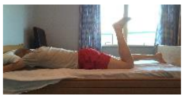	5D	Lower limb strength	Stair climbing, standing up from a chair, walking, lifting household objects
Standing hamstring curls with ankle weight 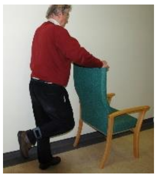	5E	Lower limb strength	Stair climbing, standing up from a chair, walking, lifting household objects
Lunge 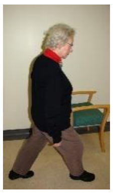	5F	Lower limb strength	Stair climbing, standing up from a chair, walking, lifting household objects

Hip abduction 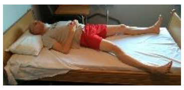	6A	Lower limb strength	Walking, dressing, getting into/out of bed
Bridging 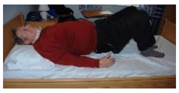	6B	Lower limb strength	Dressing, walking , standing up from a chair
Hip hitching 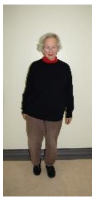	6C	Lower limb strength	Walking, dressing, getting into/out of bed
Standing hip abduction 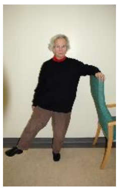	6D	Lower limb strength	Walking, dressing, getting into/out of bed, standing from a chair
Standing hip abduction with ankle weight 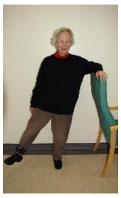	6E	Lower limb strength	Walking, dressing, getting into/out of bed, standing from a chair
Short lever hip abduction in side lying 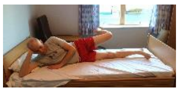	6F	Lower limb strength	Walking, dressing, getting into/out of bed
Long lever hip abduction in side lying 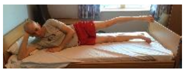	6G	Lower limb strength	Walking, dressing, getting into/out of bed
Hip abduction in side lying with ankle weight 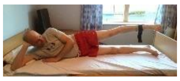	6H	Lower limb strength	Walking, dressing, getting into/out of bed

Bilateral calf raise 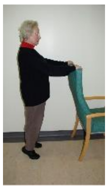	7A	Lower limb strength	Walking and stair climbing
Single calf raise 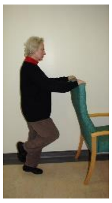	7B	Lower limb strength	Walking and stair climbing
Standing feet together 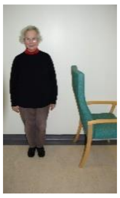	8A	Balance	Falls Prevention
Semi-tandem stance 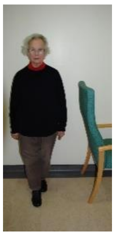	8B	Balance	Falls prevention
Tandem stance 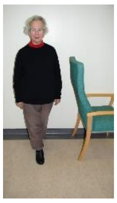	8C	Balance	Falls Prevention
Single leg stance 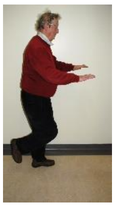	8D	Balance	Falls Prevention

Walking on the spot 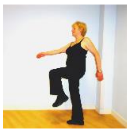	9A	Walking skills and balance	Walking and falls prevention
Waling forwards and backwards 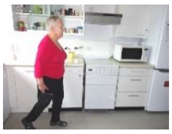	9B	Walking skills and balance	Walking a straight path and falls prevention
Side stepping 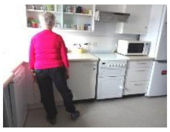	9C	Walking skills and balance	Walking, stepping around obstacles, falls prevention
Step ups 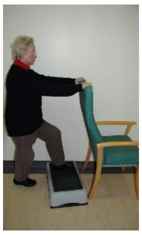	9D	Walking skills and balance	Walking, stepping onto kurbs, stair climbing, falls prevention
Mini lunge step up 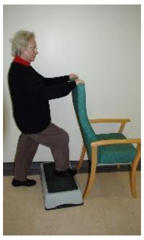	9E	Walking skills and balance	Walking, stepping onto kurbs, stair climbing, falls prevention
Turning 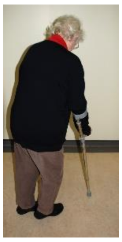	9F	Walking skills and balance	Walking with aid and falls prevention

Figure of 8 walk 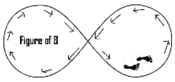	9G	Walking skills and balance	Walking both straight and curved paths, falls prevention
Tandem walking 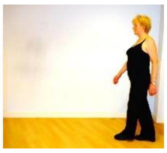	9H	Walking skills and balance	Walking and falls prevention

Usual Care

Participants randomised to usual care underwent standard in and out-patient post-operative physiotherapy as it was delivered routinely at each of the 14 research sites.

On discharge participants were given general written advice following knee arthroplasty and a home exercise programme to continue with until they were seen by an out-patient physiotherapist. To standardise usual care, participants would receive at least 1 session of physiotherapy and up to a maximum of 6 sessions which aligns with what the NHS can realistically provide. The exact duration and content of the physiotherapy sessions were at the treating physiotherapist's discretion, but in general would be between 30 and 60 minutes duration and would include a combination of range of motion and strengthening exercises, gait re-education with progression of walking aids and advice. Any differences seen in usual care across the sites is reflective of the variety of standard care delivered post knee arthroplasty across the UK.

Photographs

All participants consented to the use of their image in the treatment guide
