# Supplementary material for: Home-based rehabilitation programme compared with traditional physiotherapy for patients at risk of poor outcome after knee arthroplasty: the CORKA randomised controlled trial
Source: BMJ Open. 2021 Aug 27;11(8):e052598. doi: 10.1136/bmjopen-2021-052598 (PMC8404435; doi:10.1136/bmjopen-2021-052598)
Supplement: Author's manuscript [file bmjopen-2021-052598.draft_revisions.pdf]

BMJ Open is committed to open peer review. As part of this commitment we make the peer review history of every article we publish publicly available.

When an article is published we post the peer reviewers' comments and the authors' responses online. We also post the versions of the paper that were used during peer review. These are the versions that the peer review comments apply to.

The versions of the paper that follow are the versions that were submitted during the peer review process. They are not the versions of record or the final published versions. They should not be cited or distributed as the published version of this manuscript.

BMJ Open is an open access journal and the full, final, typeset and author-corrected version of record of the manuscript is available on our site with no access controls, subscription charges or pay-per-view fees (http://bmjopen.bmj.com).

If you have any questions on BMJ Open's open peer review process please email info.bmjopen@bmj.com

BMJ Open

COmmunity-based Rehabilitation after Knee Arthroplasty: A randomised controlled trial with economic and qualitative evaluations (CORKA Trial).

Journal:	*BMJ Open*
Manuscript ID	bmjopen-2021-052598
Article Type:	Original research
Date Submitted by the Author:	27-Apr-2021
Complete List of Authors:	Barker, Karen L.; University of Oxford, Nuffield Department of Orthopaedics, Rheumatology and Musculoskeletal Sciences<br>Room, Jonathan; Oxford University Hospitals NHS Foundation Trust Nuffield Orthopaedic Centre<br>Knight, Ruth; University of Oxford,<br>Dutton, Susan; University of Oxford<br>Toye, Francine; Oxford University Hospitals NHS Foundation Trust Nuffield Orthopaedic Centre, Nuffield Orthopaedic Centre Physiotherapy Research Unit<br>Leal, Jose; University of Oxford, Nuffield Department of Population Health<br>Kenealy, Nicola; Oxford University, Nuffield Department of Orthopaedics, Rheumatology and Musculoskeletal Sciences<br>Maia Schlüssel, Michael; University of Oxford, Nuffield Department of Orthopaedics, Rheumatology and Musculoskeletal Sciences<br>Collins, Gary; University of Oxford, Centre for Statistics in Medicine<br>Beard, David; University of Oxford, Nuffield Dept of Orthopaedics, Rheumatology and Musculoskeletal Sciences<br>Price, Andrew; University of Oxford, Nuffield Department of Orthopaedics, Rheumatology and Musculoskeletal Sciences<br>Underwood, Martin; Warwick University, Warwick Medical School<br>Drummond, Avril; University of Nottingham, Div of Rehabilitation and Ageing<br>Lamb, Sarah; University of Exeter, College of Medicine and Health; University of Oxford, NDORMS
Keywords:	Adult orthopaedics < ORTHOPAEDIC & TRAUMA SURGERY, Knee < ORTHOPAEDIC & TRAUMA SURGERY, REHABILITATION MEDICINE

SCHOLARONE™
Manuscripts

BMJ

I, the Submitting Author has the right to grant and does grant on behalf of all authors of the Work (as defined in the below author licence), an exclusive licence and/or a non-exclusive licence for contributions from authors who are: i) UK Crown employees; ii) where BMJ has agreed a CC-BY licence shall apply, and/or iii) in accordance with the terms applicable for US Federal Government officers or employees acting as part of their official duties; on a worldwide, perpetual, irrevocable, royalty-free basis to BMJ Publishing Group Ltd ("BMJ") its licensees and where the relevant Journal is co-owned by BMJ to the co-owners of the Journal, to publish the Work in this journal and any other BMJ products and to exploit all rights, as set out in our licence.

The Submitting Author accepts and understands that any supply made under these terms is made by BMJ to the Submitting Author unless you are acting as an employee on behalf of your employer or a postgraduate student of an affiliated institution which is paying any applicable article publishing charge ("APC") for Open Access articles. Where the Submitting Author wishes to make the Work available on an Open Access basis (and intends to pay the relevant APC), the terms of reuse of such Open Access shall be governed by a Creative Commons licence – details of these licences and which Creative Commons licence will apply to this Work are set out in our licence referred to above.

Other than as permitted in any relevant BMJ Author's Self Archiving Policies, I confirm this Work has not been accepted for publication elsewhere, is not being considered for publication elsewhere and does not duplicate material already published. I confirm all authors consent to publication of this Work and authorise the granting of this licence.

For peer review only

Community-based Rehabilitation after Knee Arthroplasty: A randomised controlled trial with economic and qualitative evaluations (CORKA Trial).

Karen L. Barker [1,2] *, Jon Room[1,2], Ruth Knight[3], Susan J. Dutton[3], Fran Toye[2], Jose Leal[4], Nicola Kenealy[1], Michael Maia Schussel[3], Gary Collins[3], David J. Beard[1], Andrew Price[1], Martin Underwood[5], Avril Drummond[6], Sarah E Lamb[1,7,] on behalf of CORKA Trial group.

1 Nuffield Department of Orthopaedics, Rheumatology and Musculoskeletal Sciences, University of Oxford, Oxford, UK.

2 Physiotherapy Research Unit, Nuffield Orthopaedic Centre, Oxford University Hospitals NHS Foundation Trust, Oxford, UK.

3 Centre for Statistics in Medicine, Oxford Clinical Trials Research Unit, University of Oxford, Oxford, UK.

4 Health Economics Research Centre, Nuffield Department of Population Health, University of Oxford, Oxford, UK.

5 Warwick Clinical Trials Unit, Warwick Medical School, University of Warwick, Coventry, UK

6 School of Health Sciences, University Of Nottingham, Nottingham, UK

7 School of Medicine and Health, University of Exeter

*Corresponding Author Email karen.barker@ouh.nhs.uk.

Physiotherapy Research Unit

Nuffield Orthopedic Centre

Windmill Rd, Oxford OX3 7LD

Tel no: 01865 738080

Keywords: knee arthroplasty, physical therapy, rehabilitation

Word Count : 4336

Abstract

For peer review only

Objectives: To evaluate whether a home-based rehabilitation programme for people assessed as being at risk of a poor outcome after knee arthroplasty offers superior outcomes to traditional out-patient physiotherapy.

Design: A prospective, single-blind, two-arm randomised controlled trial.

Setting: 14 National Health Service (NHS) physiotherapy departments in the UK.

Participants: 621 participants identified at high risk of a poor outcome after knee arthroplasty using a bespoke screening tool.

Interventions: A multi-component home-based rehabilitation programme delivered by rehabilitation assistants with supervision from qualified therapists versus usual care out-patient physiotherapy.

Main outcome measures: The primary outcome was the Late Life Function and Disability Instrument (LLFDI) at 12 months. Secondary outcomes were the Oxford Knee Score (OKS; a disease-specific measure of function), Knee injury and Osteoarthritis Outcome Score (KOOS) Quality of Life subscale, Physical Activity Scale for the Elderly (PASE), EQ-5D-5L, and physical function assessed using the Figure of 8 Walk test, 30 Second Chair Stand Test (30SCST), and Single Leg Stance.

Results: 621 participants were randomised. 309 were assigned to CORKA home-based rehabilitation, receiving a median 5 treatment sessions (IQR 4-7). 312 were assigned to usual care, receiving a median 4 sessions (IQR 2-6). The primary outcome, LLFDI function total score at 12 months, was collected for 279 participants (89%) in the home-based CORKA group and 287 participants (92%) in the usual-care group. No clinically or statistically significant difference was found between the groups (intention-to-treat adjusted difference = 0.49 points; 95% confidence interval -0.89 to 1.88; p = 0.48). There were no statistically significant differences between the groups on any of the patient-reported or physical secondary outcome measures at 6 or 12 months.

For peer review only

. There were 18 participants in the intervention group reporting a serious adverse event (5.8%), only one directly related to the intervention., all other adverse events recorded throughout the trial related to underlying chronic medical conditions.

Conclusions: The CORKA intervention was not superior to usual care. The trial detected no significant differences, clinical or statistical, between the two groups on either primary or secondary outcomes. CORKA offers an evaluation of an intervention utilising a different service delivery model for this patient group.

Trial Registration: ISRCTN 13517704

Funding: National Institute for Health Research Health Technology Assessment programme (HTA 12/196/08)

Article Summary

Strengths and limitations of this study:

- Large sample size and low loss to follow up which ensured sufficient numbers to make the study sufficiently powered to detect any between group differences.
- The trial was pragmatic and utilised an innovative workforce model that could readily translate into NHS practice within existing commissioning and financial constraints.
- The study offers an alternative service delivery model in line with the latest UK guidance from NICE.
- Limitations are that the screening tool did not identify patients that were a good fit of risk of poor outcome for the intervention that we developed.
- Another limitation, common to all trials of post operative physiotherapy after knee arthroplasty was the absence of a control group. As post discharge out-patient physiotherapy remains an accepted and expected core component of UK arthroplasty practice it was not deemed ethical or practicable to attempt to recruit to a no

For peer review only

treatment comparator in a group who had already been identified as at higher risk of a poor outcome.

Introduction

Knee Arthroplasty (KA) operations in the UK continue to rise with the National Joint Registry reporting over 100,000 KAs in 2017 alone (1). This is an increase of over 3% from 2010. The population receiving KA is getting older, frailer and with more co-existing health conditions. Data from patient-reported outcome measures (PROMs) have shown that most patients achieve a satisfactory outcome. However, between 10% and 15% are unsatisfied or report little or no improvement after KA (2). We do not yet know how to identify these patients or target rehabilitation to improve their outcomes.

Physiotherapy provision in the UK varies following discharge from hospital after KA but typically patients will continue to have rehabilitation needs to help restore muscle strength and endurance, range of motion, walking distance, and performance of higher-level functional activities. Patients are commonly referred for further physiotherapy in an out-patient setting to assist with these issues. The timing of this referral varies, with some hospitals advocating immediate commencement of therapy after discharge from hospital and others waiting until the first post-operative review at around 6 weeks before considering referral. This typically involves between 4 and 6 sessions of physiotherapy in an out-patient clinic setting. Patients do not routinely receive Occupational Therapy input following KA surgery unless they are highlighted to have particular difficulties with their daily activities or have specific barriers to discharge home. Concern has been raised that many exercise programmes lack adequate intensity to enable optimal recovery (3,4). Internationally, where much longer courses of physiotherapy are often provided, research has indicated that 12-18 hours of physiotherapy (5) or a mean of 17 visits (6) may be needed to produce a benefit. These levels of care are substantially higher than those provided in the UK and, in the current economic climate, may be more than the National Health Service (NHS) can afford.

For peer review only

Given the rising number of KA operations, the relatively limited therapy resources available, and the increasing age and frailty of patients receiving this surgery, it is important that rehabilitation resources are concentrated on those patients who need the most help to achieve a good outcome. Not all patients need post-operative out-patient physiotherapy and some will make a full recovery using self-directed rehabilitation (7–10). Current rehabilitation strategies do not meet the needs of all patients, particularly those who are socially isolated, do not have easy access to transport, and are frail. It is thus pertinent to focus our resources on those who are most likely to be at risk of a poor outcome, least likely to be able to engage with a self-management approach, and able to benefit most from rehabilitation input To maximise access to therapy and to provide this within a similar cost envelope to current practice we designed an intervention to be delivered in the patients' homes utilising a workforce model that utilise rehabilitation assistants supported by qualified therapy staff, drawing on the success of this model in delivering therapy programmes such as the OTAGO falls prevention intervention (11). This trial was carried out to determine if a targeted home-based intervention for those at risk of poor outcome, delivered by a mix of qualified staff and rehabilitation assistants, could improve patient reported and physical function outcomes at 1 year following KA.

Methods

The l CORKA trial was a multicentre prospective, single blinded, two-arm randomised controlled trial with assessment for the clinical outcomes at baseline, 6 and 12 months. The study was carried out according to the published protocol (12).

Recruitment

Participants were recruited from 14 NHS Trusts across England. Patients aged 55 years and over due to undergo KA were sent information concerning the trial prior to their pre-operative assessment appointment. Those expressing interest were screened in the clinic using a screening tool developed for the trial. This aimed to identify patients at risk of a poorer outcome based on data collected as part of standard pre-operative assessment screening. The tool was developed using all data from the Knee Arthroplasty Trial (KAT) dataset and further

For peer review only

details of its development has been previously published (13). The screening tool had a range of possible scores from 0 – 10, patients with a score of 5 or more were classified as being at increased risk of a poor outcome after 1 year defined as an Oxford Knee Score of 26 or less. [Appendix A]. The performance of the model was fair, as measured by its discrimination. Participants who were eligible were screened again post-operatively to confirm their eligibility and consent. Exclusion criteria included any major peri-operative complications, further surgery planned within the next 12 months and absolute contraindications to exercise. Screening was performed by research clinicians at the local sites, and screening logs recorded the reasons for ineligibility and why eligible patients declined to participate.

Randomisation

Randomisation took place on the third post-operative day, or on hospital discharge if earlier. Participants were randomly allocated by a computer-generated system to either 'usual care' or 'home-based rehabilitation programme' in a 1:1 ratio using permuted blocks of various sizes (sizes 2, 4 and 6 in a 1:2:1 ratio), and this was stratified by recruitment site. Participants and those delivering the rehabilitation were aware of the treatment allocation due to the nature of the intervention. Those carrying out follow-up outcome measurements remained blinded to treatment allocation.

Interventions

<u>CORKA home-based intervention</u>

The CORKA home-based intervention is a multicomponent rehabilitation programme developed in collaboration with clinicians and patients; it has been described in detail in a previous publication (14). Its aim was to improve the function and participation in activities of participants at risk of a poor outcome after KA surgery. The primary component of the rehabilitation programme was an individually adapted exercise package conducted in participants' own homes. Additional components consisted of functional task practice, adherence approaches, and, if required, the provision of appropriate aids and equipment. The CORKA intervention started up to 4 weeks after surgery, with most participants seen within 2

For peer review only

weeks. In the period between surgery and the initial appointment participants continued with an exercise programme given to them by ward physiotherapists in line with standard current practice. It consisted of an initial assessment appointment and up to 6 follow-up sessions. The physiotherapist undertook the initial assessment and prescription of the exercise programme, observed by the rehabilitation assistant. For future sessions the rehabilitation assistant continued the programme and modified as required. The physiotherapist undertook one further session in the middle of the programme to review the participant's progress and their exercise programme. The way this was operationalised is outlined in Appendix B.

Usual care

Those allocated to the usual-care arm received standard post-operative physiotherapy. Usual care after KA surgery could vary considerably across the trial's different UK locations. However, it was highly likely that usual care would include several of the following: between 1 and 6 sessions of physiotherapy in an out-patient setting, class-based setting, or hydrotherapy; written advice on home exercises on discharge from hospital; and an assessment of any potential home requirements or barriers to discharge by an occupational therapist. To standardise usual care as much as possible, participants were expected to attend a minimum of 1 session and a maximum of 6 sessions of usual care physiotherapy.

Outcomes

Baseline data were collected face-to-face no more than four weeks before surgery. Follow-up data collection was by face-to-face clinical assessments at 6 and 12 months following randomisation. Where face-to-face assessment was not possible, postal and telephone data collection methods were used to obtain self-reported core data.

Primary outcome

The primary outcome was the Late Life Function and Disability Instrument (LLFDI) overall function score. It was developed specifically to assess change in community-dwelling older

For peer review only

adults. It assesses and responds to meaningful change in two distinct outcomes: a person's ability to do discrete actions or activities using a 32-item *function* component (Primary outcome) and a person's performance of socially defined life tasks using a 16-item *disability* component (secondary outcome).

Secondary outcomes

Secondary outcomes consisted of self-reported and physical measures. The self-reported measures were the Oxford Knee Score (OKS), Physical Activity Scale for the Elderly (PASE), Knee Injury and Osteoarthritis Outcome Score (KOOS) Quality of Life subscale, and EQ-5D-5L questionnaires. The physical measures were the Figure of 8 Walk Test (F8WT), 30 Second Chair Stand Test (30SCST), and Single Leg Stance (SLS). A health resource diary collected the exercises undertaken, medication taken, use of healthcare services and personnel, and falls.

Analysis

A detailed statistical analysis plan has previously been published (15). The LLFDI score was chosen as a clinically relevant outcome for the population and the sample size calculation was therefore based on this variable. As there is no formal minimally clinically important difference for the LLFDI the calculation was based on a moderately small standard effect size of 0.275 which gave a sample of 620 participants (310 per arm) with 90% power, 5% (two-sided) significance, allowing for 10% loss to follow up.

Two analysis populations were considered: the intention-to-treat (ITT) population and the per-protocol (PP) population. The ITT population included all randomised participants analysed according to their allocated intervention. The PP population included only participants who received at least one session of their allocated intervention, did not receive more treatment than intended (more than 6 sessions of usual care or 7 sessions of home-based rehabilitation), and provided follow-up data.

For peer review only

LLFDI function scores at 6 and 12-months post-randomisation were summarised by treatment group and analysed using a linear mixed effects model with repeated measures adjusted for baseline score and recruiting site (stratification factor). Time was treated as categorical and an interaction between the outcome measurement time point and randomised group was included to allow the treatment effect to be estimated at each time point, reported as the adjusted mean difference in LLFDI between groups with 95% CI and associated p-value. The underlying assumptions of this model were assessed. Twelve months post-randomisation was considered the primary endpoint. The primary analysis was performed for the ITT population using multiple imputation to impute missing data. As sensitivity analyses this was repeated for the ITT population using available cases and the PP population using available cases.

Linear mixed effects models with repeated measures, similar to that described for the primary outcome, were used to analyse each of the secondary PROMs (LLFDI disability limitation, LLFDI disability frequency, OKS, KOOS QoL, PASE, EQ-5D- 5L utility and VAS), 30SCST (number of stands), and F8WT (time and steps). Differences between the two groups in SLS times were compared using a Wilcoxon rank-sum test since the assumptions of the linear mixed effects model were not satisfied. All of these analyses were performed for the ITT population using available cases only. For the key secondary outcomes (LLFDI disability limitation, LLFDI disability frequency, OKS, and KOOS QoL), these analyses were repeated for the ITT population using multiple imputation (imputation model as outlined for the primary outcome) and the PP population using available cases. To estimate the effect of compliance on the primary outcome we used complier average causal effect (CACE) analysis (16).

The number and proportion of SAEs were reported by treatment group, as well as the number and proportion of participants experiencing an SAE. The rates were compared across treatment groups.

Patient and Public Involvement

For peer review only

The study was developed with a group of service users that identified concern about the access to rehabilitation for older more frail patients after knee arthroplasty. The intervention was developed with active involvement of PPI stakeholders who worked alongside us to develop the intervention. A PPI representative collaborated on the application for funding and was a full voting member of the trial steering group which benefitted from their expertise and active engagement throughout the period of the study.

Results

2,788 patients were screened for eligibility. and 621 participants were randomised, 312 to usual care and 309 to the home based rehabilitation programme. A CONSORT flow chart summarises the flow of participants through the trial including details on the number of participants randomised and the numbers allocated to and receiving at least 1 session of each treatment (See Figure 1). Of the 621 participants, 34 (5.5%) withdrew from follow-up during the course of the trial. Three participants died between the 6 and 12 month follow ups.

The mean age of participants was 70.4 (SD 8.07), 59.7% were female and mean BMI was 31.5 (SD 4.74). Most participants scored 5 or 6 on the screening tool (79.5%) with 5 being the minimum score needed to be eligible for the study and indicating a risk of poor outcome. The baseline characteristics of the participants is summarised in Table 1.

Participants were defined as complying with usual care if they attended at least 1 treatment session and for home-based rehabilitation at least 4 treatment sessions. We used different thresholds for the two treatments as usual care varied considerably between sites including as little as one session. Therefore, compliance in this arm was defined as participants receiving at least one session. For the intervention arm, it was pre-specified that at least 4 of the 7 planned sessions were required to be compliant. Therapy compliance was 95.2% in usual care and 87.1% for the CORKA intervention. The median number of sessions for usual care was 4 (IQR 2,6) and

For peer review only

for the CORKA intervention was 5 (IQR 4,7). CACE analysis was performed using the definition of compliance with the intervention arm (16)

Primary outcome analysis

For the ITT analysis there was no statistically significant difference between the two treatment groups at the primary time point of 12 months post-randomisation (adjusted difference 0.49, 95% CI: -0.89 to 1.88) or at 6 months (adjusted difference 0.66, 95% CI:-0.70, 2.03). This equates to a standardised effect size of 0.03 at 12 months.

Trends in LLFDI function scores over time are plotted for each treatment group in Figure 2. There was a substantial improvement between baseline and 6 months in both groups, with minimal additional difference between 6 and 12 months post-randomisation.

Secondary outcome analysis

For the secondary PROMS ITT analysis, no significant differences between the two treatment groups at either time point (6 and 12 months) was identified for any of the secondary outcomes (see Table 2). The sensitivity analyses of the key secondary PROMS identified only one difference, of borderline statistical significance, for the LLFDI disability limitation at 6 months using the ITT population and multiple imputation (adjusted difference 2.67, 95% CI: 0.14 to 5.19,).

The physical measures are summarised by treatment group over time in Table 3. With the exception of one of the SLS tests, there were no differences in change in performance between the groups on the physical measures.

The total number of SAEs experienced in this trial was low; 18 events in 14 participants in the usual care group (4.5%) and 20 events in 18 participants in the CORKA intervention group (5.8%), experienced . Adverse events were independently reviewed and categorised using the

For peer review only

System Organ Classes codes and as related to the intervention or not. For all SAEs only one was classified as 'possibly related'. In total there were 7 SAEs in the musculoskeletal and connective tissue disorder category, with one categorised as related to the treatment interventions (Table 4). The majority of the SAEs were related to medical conditions unconnected with the participant's KA surgery and were classified into categories such as cardiac disorders, renal and urinary disorder, or immune system disorders. There was not a significant difference in the number of participants experiencing an SAE between the two groups (Risk difference 1.3%, 95% CI -2.1% to 4.8%).

Discussion

We found no statistical or clinically meaningful differences were found between CORKA and usual out-patient physiotherapy. Outcomes across a range of measures were closely matched irrespective of which of the two interventions was received. The use of a workforce model using rehabilitation assistants made treatment cheaper to deliver and did not result in any increase in adverse events.

Strengths and limitations of the study

Strengths of this study include the large sample size and low loss to follow up which ensured sufficient numbers to make the study sufficiently powered to detect any between group differences. The trial was pragmatic and utilised an innovative workforce model that could readily translate into NHS practice within existing commissioning and financial constraints. The study offers an alternative service delivery model in line with the latest UK guidance from NICE. Limitations are that the screening tool did not identify patients that were a good fit of risk of poor outcome for the intervention that we developed. It has not been validated, but uses items that are used by many knee arthroplasty trials to determine entry, these are broad and inclusive, reflecting common clinical practice. Recent recommendations from the UK National Institute for Health and Clinical Excellence noted that it would be useful if a tool existed that indicated those who would benefit from supervised rehabilitation and recognised that there is a

For peer review only

significant proportion of patients to whom this will apply [17]. In developing our screening tool for the trial we attempted just this, but recognise that there was not a good match between the patients screened and the intervention that we developed.

Another limitation, common to all trials of post-operative physiotherapy after knee arthroplasty, was the absence of a control group. As out-patient physiotherapy remains an accepted and expected core component of UK arthroplasty practice it was not deemed ethical or practicable to attempt to recruit to a no treatment comparator in a group who had already been identified as at higher risk of a poor outcome. As with most physiotherapy trials it was not feasible to blind patients or therapists to treatment allocation. We also chose to deliver an intervention that met current commissioning guidance of around six sessions. It is arguable that more intensive treatment as is common in other countries may have been more effective, but this is not supported by studies using longer interventions [18-23]

We chose to use the LLFDI as our primary outcome over a more knee focused outcome as it is designed to map to the WHO ICF and assess change in community dwelling older adults encompassing the range of activities relevant for older, potentially frailer patients after arthroplasty. At the time of designing the trial the MCID was not published. Subsequently, Beauchamp et al (2019) reported that substantial changes are reflected in a 5-point change in the overall function scale [24]. We found substantial changes from baseline to 1 year within both groups, but no between group effect. The CORKA home-based intervention showed an overall change of 8.9 points and usual care 8.7 points, indicating that the combined effect of surgery and rehabilitation unsurprisingly produced a highly significant improvement in function. Most of this improvement was probably due to the surgical procedure, but we cannot disaggregate the contribution of physiotherapy to quantify the contribution of one without the other. We found no significant between group difference. It is arguable that a more specific measure targeted at KA may have been a better choice. We included the OKS as a secondary measure to address this potential limitation. The results of the LLFDI and OKS were well-matched.

Implications for patients and policy

For peer review only

The benefit of post-operative physiotherapeutic interventions are poorly established. A recent evidence review by NICE, of all patients receiving arthroplasty surgery, found no clinically important differences in quality of life, PROMS or functional outcomes between group or individually based supervision and self-directed rehabilitation. They recommended self-directed rehabilitation as an acceptable option for people who are well enough and have personal circumstances that make it possible, but recognised that there are likely to be subgroups who will benefit from a more supervised rehabilitation programme (17). The CORKA trial is one of few trials to specifically target individuals who are at risk of poor outcome post KA. The TRIO trial (25) also targeted physiotherapy at patients considered at risk of poor outcome, based upon Oxford Knee Score at 6 weeks. Similar to this study they found no statistical differences between the two treatment arms of outpatient physiotherapy and a single physiotherapy review and home exercise based regimen.

Although we screened for patients deemed at risk of poor outcome the OKS of 37.8 points was higher than the reported average for all knee arthroplasty patients at one year of 36 points [ref]. The TRIO trial similarly found their results, although a little lower, were similar to the unselected average. Most of this improvement was probably due to the surgical procedure, but we cannot disaggregate the contribution of physiotherapy to quantify the contribution of one without the other.

The CORKA intervention was based on pre-operative outcome measures, with a home-based rehabilitation programme. There may also be benefits to home supervised rehabilitation that are not captured by the current evidence or the outcome measures used in existing studies. For example, participants in our embedded qualitative study were very positive about the CORKA home-based intervention and the supervision provided by the therapists and rehabilitation assistants (27). The CORKA home-based intervention could also benefit, for example, those too frail to travel a significant distance to a larger hospital. Using the CORKA combination of qualified therapists and rehabilitation assistants might be a practical option for certain remote areas of the UK or countries that have large geographical areas not covered by therapists. This

For peer review only

suggests there is an argument for choice, according to personal preference or the practicalities of service provision.

It also addresses the workforce shortage issue by using an innovative workforce model of advanced rehabilitation assistants, moving UK service provision closer to that which has been proven to be effective in North America, where the use of physical therapy assistant graded staff is well embedded. This is a particular concern given both the projected increased need for joint arthroplasty over the next decade to accommodate an ageing population and the pressure of potential reductions in NHS funding. Evaluating the value of treatment modalities offered to these patients is crucial because many more patients are being discharged home earlier from the acute setting leaving less time available for acute physical recovery, rehabilitation, and education in hospital, increasing the potential burden of care for these patients and their families.

Future research

Further research should focus on developing a screening tool that is more sensitive in identifying those patients who will benefit from additional input, as recommended by the NICE guidance on primary joint replacement (17).

The CORKA home-based intervention was delivered by rehabilitation assistants supervised by qualified therapists in a ratio of 5:2 sessions. Given the workforce challenges in the NHS further work looking at different workforce models and interventions solely using rehabilitation assistants should be explored.

Conclusions

This large randomised controlled trial found no important differences in outcomes when KA rehabilitation was delivered using either a home-based, rehabilitation assistant delivered

For peer review only

rehabilitation package or a traditional out-patient physiotherapy model. Home based rehabilitation was feasible but did not confer benefits over standard out patient physiotherapy.

Contributorship Statement:

Karen L Barker (Professor of Physiotherapy) – Chief Investigator, led funding application, trial conception and design, development of interventions, supervision, and writing and reviewing report.

Jonathan Room (Research Physiotherapist) – Principal Investigator, development of intervention, and writing and reviewing report.

Ruth Knight (Statistician) – Conducting statistical analysis, and writing and reviewing report.
Seamus Kent (Health Economist) – Conducting economic analysis, and writing and reviewing report.

Susan Dutton (Statistician) – Designing and conducting statistical analysis, and writing and reviewing report.

Fran Toye (Qualitative lead) -, conducting interviews and analysis, and writing chapter for report.

Jose Leal (Health Economist) – Designing and conducting economic analysis, supervision, and writing and reviewing report.

Nicola Kenealy (Trial Manager) – Trial management and writing and reviewing report.

For peer review only

Michael Maia Schlussel (Statistician) – Screening tool development and writing for report.

Gary Collins (co-applicant) – Supervised screening tool development.

David Beard (co-applicant) – Clinical trials expertise and contributed to report.

Andrew Price (co-applicant) – Surgical expertise and contributed to report.

Martin Underwood (co-applicant) – Primary care expertise and contributed to report.

Avril Drummond (co-applicant) – Occupational therapy expertise and contributed to report.

Sarah Lamb (co-applicant) – Clinical trials expertise, key member of trial management group, and writing and reviewing report.

Acknowledgements:
The authors would like to acknowledge the site principal investigators and research clinicians

Leon Palmer-Wilson, Ana Glennon (Ashford Hospital), Helen Wilson (Countess of Chester Hospital), Christian Brookes, Denise Hill (Dorset County Hospital), Susan Dowdle, Hazel Burt (Dorset HealthCare Hospital), Jane Harrison (Epsom Hospital), Sarah Adcock, Justine Theaker (Manchester Royal Infirmary), Gladys Nadar Arulmani (Medway Community Hospital), Sunil Jain (Medway Maritime Hospital), Mike Reed (North Tyneside General Hospital), Jonathan Room (Oxford University Hospitals), Genevieve Simpson, Gemma Knight (Royal United Hospitals Bath), Tricia Monroe (Moorgreen Hospital), Gareth Stephens, Sarah Rich (Royal Orthopaedic Hospital Birmingham).

Funding support: This trial was funded by the National Institute for Health Research Health Technology Assessment programme (HTA 12/196/08)

For peer review only

Conflict of Interest:

G Collins is a member of the HTA Commissioning Board

A Price has a consultancy with Zimmer Biomet, outside the submitted work

M Underwood has grants from NIHR, during the conduct of the study; grants from NIHR, Personal fees from NIHR, Personal fees from NICE, non financial support from Stryker PLC, grants from SERCO ltd.

S Lamb has grants from NIHR Health Technology Assessment Programme during the conduct of the study and is a member of the following Boards:

HTA Additional Capacity Funding Board 2010-2015

HTA Clinicial Trials Board 2010-2015

HTA End of Life Care and Add on Studies 2015-2015

HTA Funding Boards Policy Group (Formally CSG) 2010-2015

HTA MNCH Methods Group 2013-2015

HTA Post-board funding teleconference (PG members to attend) 2010-2015

HTA Primary Care Themed Call board 2013-2014

HTA Prioritisation Group 2014-2015

NIHR CTU Standing Advisory Committee 2012-2016

Ethical approvals

The study protocol was approved by the South-Central Research Ethics Committee (Reference 15/SC/0019). Ethics permission was obtained for all participating sites.

Data Sharing

Data are available upon request, please contact the corresponding author

For peer review only

References

1. National Joint Registry for England, Wales NI and the I of M. 15th Annual Report 2018. 15th Annu Rep. 2018;
2. Jones CA, Beaupre LA, Johnston DWC, Suarez-Almazor ME. Total Joint Arthroplasties: Current Concepts of Patient Outcomes after Surgery. Rheumatic Disease Clinics of North America. 2007.
3. Naylor J, Harmer A, Fransen M, Crosbie J, Innes L. Status of physiotherapy rehabilitation after total knee replacement in Australia. Physiother Res Int. 2006;
4. Westby MD, Kennedy D, Jones DL, Jones A, Doyle-Waters M, Backman C. Post-acute physiotherapy for primary total knee arthroplasty. Cochrane Database of Systematic Reviews. 2008.
5. Moffet H, Collet JP, Shapiro SH, Paradis G, Marquis F, Roy L. Effectiveness of intensive rehabilitation on functional ability and quality of life after first total knee arthroplasty: A single-blind randomized controlled trial. Arch Phys Med Rehabil. 2004;
6. Petterson SC, Mizner RL, Stevens JE, Raisis LEO, Bodenstab A, Newcomb W, et al. Improved function from progressive strengthening interventions after total knee arthroplasty: A randomized clinical trial with an imbedded prospective cohort. Arthritis Care Res. 2009;
7. Rajan RA, Pack Y, Jackson H, Gillies C, Asirvatham R. No need for outpatient physiotherapy following total knee arthroplasty: A randomized trial of 120 patients. Acta Orthop Scand. 2004;
8. Piva SR, Moore CG, Schneider M, Gil AB, Almeida GJ, Irrgang JJ. A randomized trial to compare exercise treatment methods for patients after total knee replacement: protocol paper. BMC Musculoskelet Disord. 2015;
9. Artz N, Dixon S, Wylde V, Marques E, Beswick AD, Lenguerrand E, et al. Comparison of group-based outpatient physiotherapy with usual care after total knee replacement: A feasibility study for a randomized controlled trial. Clin Rehabil. 2017;
10. Fillingham YA, Darrith B, Lonner JH, Culvern C, Crizer M, Della Valle CJ. Formal Physical Therapy May Not Be Necessary After Unicompartmental Knee Arthroplasty: A Randomized Clinical Trial. J Arthroplasty. 2018;
11. Hubert TE, Smith ML, Goto L, Jiang L, Ory MG. Otago Exercise Program in the United States: Comparison of 2 Implementation Models. Phys Ther. 2017;97(2):187-97.
12. Barker KL, Beard D, Price A, Toye F, Underwood M, Drummond A, Collins G, Dutton S, Campbell H, Kenealey N, Room J, Lamb SE. Community-based rehabilitation after knee arthroplasty (CORKA): study protocol for a randomised controlled trial. Trials 2016 Oct 13;17(1):501.
13. Schlussel M, Collins G, Dutton S, Barker K. Development of the CORKA trial screening tool for identifying patients at increased risk of poor outcome following knee replacement. Trials. 2017;18:S1, P430.
14. Room J, Batting M, Barker KL. Development of a functional rehabilitation intervention for post knee arthroplasty patients: community based Rehabilitation post Knee Arthroplasty (CORKA) trial. Physiotherapy. 2020 Mar;106:52-64. doi: 10.1016/j.physio.2019.06.004 (United Kingdom). 2020;

For peer review only - http://bmjopen.bmj.com/site/about/guidelines.xhtml

15. Vadher K, Knight R, Barker KL, Dutton SJ. Community based rehabilitation after knee arthroplasty (CORKA): statistical analysis plan for a randomised controlled trial. Trials. 2018 Nov 19;19(1):638
16. Dunn G, Maracy M, Tomenson B. Estimating treatment effects from randomized clinical trials with noncompliance and loss to follow-up: the role of instrumental variable methods. Stat Methods Med Res. 2005;14(4):369-95.
17. National Institute for Health and Care Excellence. Joint replacement (primary): hip, knee and shoulder [R] Evidence review for outpatient hip and knee postoperative rehabilitation. NICE guideline [NG157]. 2020. www.nice.org.uk/guidance/ng157. Accessed 16.06.2020..
18. Hamilton DF, Loth FC, MacDonald DJ, MacFarlane GJ, Beard DJ, Simpson AHRW, et al. Exploring variation in patient access of post-discharge physiotherapy following total hip and knee arthroplasty under a choice based system in the UK: An observational cohort study. BMJ Open. 2019;
19. Khan F, Ng L, Gonzalez S, Hale T, Turner-Stokes L. Multidisciplinary rehabilitation programmes following joint replacement at the hip and knee in chronic arthropathy. Cochrane Database of Systematic Reviews. 2008.
20. Artz N, Elvers KT, Lowe CM, Sackley C, Jepson P, Beswick AD. Effectiveness of physiotherapy exercise following total knee replacement: Systematic review and meta-analysis. BMC Musculoskeletal Disorders. 2015.
21. Roos EM. Effectiveness and practice variation of rehabilitation after joint replacement. Current Opinion in Rheumatology. 2003.
22. Smith TO, Dainty JR, Clark EM, Whitehouse MR, Price AJ, MacGregor AJ. Demographic and geographical variability in physiotherapy provision following hip and knee replacement. An analysis from the National Joint Registry for England, Wales, Northern Ireland and the Isle of Man. Physiotherapy 2020;106:1-11. doi:10.1016/j. physio.2019.11.003
23. Buhagiar MA, Naylor JM, Harris IA, Xuan W, Adie S, Lewin A. Assessment of Outcomes of Inpatient or Clinic-Based vs Home-Based Rehabilitation After Total Knee Arthroplasty: A Systematic Review and Meta-analysis. JAMA Netw Open 2019;2:e192810. doi:10.1001/ jamanetworkopen.2019.2810

24 Beauchamp MK, Ward RE, Jette AM, Bean JF. Meaningful Change Estimates for the Late-Life Function and Disability Instrument in Older Adults. J Gerontol A Biol Sci Med Sci. 2019;74(4):556-9.

25. Hamilton DF, Beard DJ, Barker KL, Macfarlane GJ, Tuck CE, Stoddart A, Wilton T, Hutchinson JD, Murray GD, Simpson AHRW. Targeting rehabilitation to improve outcomes after total knee arthroplasty in patients at risk of poor outcomes: randomised controlled trial. BMJ 2020 Oct 13;371:m3576.

25. Barker KL, Room J, Knight R, Dutton SJ, Toye F, Leal J, Kent S, Kenealy N, Schussel MM, Collins G, Beard DJ, Price A, Underwood M, Drummond A, Cook E, Lamb SE. COmmunity-based Rehabilitation after Knee Arthroplasty: A randomised controlled trial with

economic and qualitative evaluations (CORKA Trial). Health Technology Asessment: 2020; 24: 1-116

For peer review only

Table 1 - Descriptive characteristics at baseline by treatment group

	Usual care (n = 312)	Home-based rehab (n = 309)	Total (n = 621)
**Gender[a]**			
Male	125 (40.1%)	125 (40.5%)	250 (40.3%)
Female	187 (59.9%)	184 (59.5%)	371 (59.7%)
**Age (years)[b]**	70.18 (8.14)	70.67 (8.01)	70.42 (8.07)
**BMI[b]**	31.65 (4.99)	31.34 (4.48)	31.50 (4.74)
**Side of operation[a]**			
Right	169 (54.2%)	169 (54.7%)	338 (54.4%)
Left	142 (45.5%)	139 (45.0%)	281 (45.2%)
Not recorded	1 (0.3%)	1 (0.3%)	2 (0.3%)
**Knee arthroplasty type[a]**			
TKA	229 (73.4%)	231 (74.8%)	460 (74.1%)
UKA	82 (26.3%)	77 (24.9%)	159 (25.6%)
Not recorded	1 (0.3%)	1 (0.3%)	2 (0.3%)
**ASA grade[a]**			
Healthy	38 (12.2%)	55 (17.8%)	93 (15.0%)
Mild systemic disease	218 (69.9%)	202 (65.4%)	420 (67.6%)
Severe systemic disease	43 (13.8%)	44 (14.2%)	87 (14.0%)
Not recorded	13 (4.2%)	8 (2.6%)	21 (3.4%)
**Falls in the last year[a]**			
Yes	77 (24.7%)	89 (28.8%)	166 (26.7%)
No	235 (75.3%)	220 (71.2%)	455 (73.3%)
**If yes, number of falls[c]**	1 (1, 3)	2 (1, 3)	2 (1, 3)
**Previous lower limb surgery[a]**			
Yes	200 (64.1%)	189 (61.2%)	389 (62.6%)
No	112 (35.9%)	120 (38.8%)	232 (37.4%)
**Screening tool score[c]**	6 (5, 6)	6 (5, 6)	6 (5, 6)

For peer review only

**Charnley ABC[a]**			
A - single knee arthroplasty	134 (42.9%)	138 (44.7%)	272 (43.8%)
B - both knees affected	140 (44.9%)	145 (46.9%)	285 (45.9%)
C - multiple joint disease/other disability	38 (12.2%)	26 (8.4%)	64 (10.3%)
**Stairs mobility[a]**			
Normal	19 (6.1%)	19 (6.1%)	38 (6.1%)
One step at a time	34 (10.9%)	39 (12.6%)	73 (11.8%)
Down with rail	18 (5.8%)	19 (6.1%)	37 (6.0%)
Up/down with rail	225 (72.1%)	216 (69.9%)	441 (71.0%)
Unable down	2 (0.6%)	3 (1.0%)	5 (0.8%)
Unable	14 (4.5%)	12 (3.9%)	26 (4.2%)
Missing	0 (0.0%)	1 (0.3%)	1 (0.2%)
**Support mobility[a]**			
None	178 (57.1%)	178 (57.6%)	356 (57.3%)
Stick outdoors	83 (26.6%)	78 (25.2%)	161 (25.9%)
Stick always	34 (10.9%)	31 (10.0%)	65 (10.5%)
2 sticks	6 (1.9%)	7 (2.3%)	13 (2.1%)
2 crutches	5 (1.6%)	7 (2.3%)	12 (1.9%)
Walking frame	6 (1.9%)	8 (2.6%)	14 (2.3%)
**Functional comorbidity index[d]**			
0	189 (60.6%)	176 (57.0%)	365 (58.8%)
1-3	112 (35.9%)	125 (40.5%)	237 (38.2%)
4-6	8 (2.6%)	6 (1.9%)	14 (2.3%)
7+	2 (0.6%)	0 (0.0%)	2 (0.3%)
Missing	1 (0.3%)	2 (0.6%)	3 (0.5%)

ASA = American Society of Anaesthesiologists; BMI = body mass index; TKA = total knee arthroplasty; UKA = unicompartmental knee arthroplasty.
[a] These values are reported as numbers and percentages
[b] These values are reported as means and standard deviations
[c] These values are reported as medians and interquartile ranges
[d] The functional comorbidity index (85) counts the number of comorbidities experienced by each patient, giving more weight to more severe conditions. This was categorised in four groups: (i) no comorbidities (0), (ii) small number of comorbidities (1-3), (iii) medium number of comorbidities (4-6), and (iv) high number of comorbidities (7+).

For peer review only

For peer review only

Table 2 - Comparison of treatment groups for secondary outcomes (intention-to-treat population using available cases)

		Usual care		Home-based rehab		Adjusted difference (95% CI)	p-value
		Mean (SD)	Total	Mean (SD)	Total		
**LLFDI Disability (frequency)**	**Baseline**	51.28 (7.25)	311	51.55 (7.46)	308	-	-
	**6 months**	54.27 (6.86)	283	54.91 (6.85)	275	0.64 (-0.50, 1.78)	0.27
	**12 months**	54.13 (6.87)	287	55.05 (6.87)	279	0.93 (-0.21, 2.06)	0.11
**LLFDI Disability (limitation)**	**Baseline**	66.26 (11.96)	312	66.25 (12.48)	305	-	-
	**6 months**	72.33 (15.25)	284	74.72 (15.24)	275	2.39 (-0.14, 4.92)	0.06
	**12 months**	74.10 (15.27)	287	74.75 (15.28)	277	0.65 (-1.87, 3.18)	0.61
**OKS**	**Baseline**	20.59 (7.50)	312	20.81 (7.31)	308	-	-
	**6 months**	35.69 (7.69)	284	36.23 (7.69)	277	0.54 (-0.73, 1.81)	0.40
	**12 months**	37.34 (7.71)	287	37.80 (7.71)	278	0.46 (-0.81, 1.73)	0.48
**KOOS – QoL subscale**	**Baseline**	25.50 (16.58)	301	25.50 (15.89)	300	-	-
	**6 months**	61.26 (22.31)	273	62.47 (22.29)	269	1.21 (-2.55, 4.97)	0.53
	**12 months**	65.53 (22.37)	277	65.26 (22.36)	271	-0.27 (-4.02, 3.47)	0.89
**PASE**	**Baseline**	121.24 (73.90)	302	115.11 (72.95)	297	-	-
	**6 months**	150.62 (66.72)	259	149.41 (66.46)	245	-1.22 (-12.86, 10.42)	0.84
	**12 months**	156.58 (66.82)	263	154.63 (66.73)	251	-1.95 (-13.51, 9.61)	0.74
**EQ5D-5L Utility**	**Baseline**	0.52 (0.22)	308	0.52 (0.23)	308	-	-
	**6 months**	0.75 (0.18)	277	0.75 (0.18)	275	0.00 (-0.03, 0.03)	0.85
	**12 months**	0.76 (0.18)	282	0.76 (0.18)	278	-0.00 (-0.03, 0.03)	0.99
**EQ5D-5LVAS**	**Baseline**	68.53 (19.46)	312	68.03 (19.13)	308	-	-
	**6 months**	75.81 (14.55)	283	75.51 (14.54)	275	-0.30 (-2.71, 2.11)	0.81
	**12 months**	76.80 (14.57)	287	77.05 (14.58)	278	0.24 (-2.16, 2.65)	0.84

For peer review only

Notes: LLFDI = Late Life Function and Disability Instrument, each LLFDI disability scale ranges from 0 to 100 with higher scores indicating less disability. OKS = Oxford Knee Score, scores range from 0 to 48 with higher scores indicating better knee function. KOOS = Knee injury and Osteoarthritis Outcome Score, scores range from 0 to 100 with higher scores indicating better QoL. PASE = Physical Activity Scale for the Elderly, higher scores indicate greater levels of physical activity. EQ5D-5L utility scores range from -0.594 to 1 and EQ5D-5L visual analogue scale (VAS) ranges from 0 to 100, in both cases higher scores indicate better quality of life. CI = confidence interval; SD = standard deviation.

For peer review only

Table 3 - Comparison of physical measures between treatment groups at follow-up time points

	Usual care (n = 312)		Home-based rehab (n = 309)		Adjusted difference (95% CI)	p-value
	Summary	N	Summary	N		
***30SCST # stands*[a]**						
**Baseline**	8.2 (3.5)	312	8.4 (3.7)	309	-	-
**6 months**	11.1 (3.1)	278	11.0 (3.1)	275	-0.06 (-0.548, 0.46)	0.82
**12 months**	11.7 (3.1)	279	11.5 (3.1)	276	-0.22 (-0.74, 0.3)	0.41
***F8WT (seconds)*[a]**						
**Baseline**	11.8 (5.4)	312	12.1 (4.9)	309	-	-
**6 months**	10.2 (3.6)	277	9.6 (3.6)	275	-0.57 (-1.17, 0.04)	0.07
**12 months**	9.3 (3.6)	280	9.1 (3.6)	274	-0.17 (-0.78, 0.43)	0.58
***F8WT steps*[a]**						
**Baseline**	16.4 (4.5)	312	17.3 (5.0)	309	-	-
**6 months**	15.5 (3.2)	277	15.2 (3.2)	275	-0.36 (-0.91, 0.18)	0.19
**12 months**	15.2 (3.2)	280	14.9 (3.3)	274	-0.23 (-0.77, 0.31)	0.40
***KA leg SLS average (seconds)*[a]**						
**Baseline**	5.3 (2.6, 13.0)	310	4.5 (1.9, 14.5)	308	-	-
**6 months**	10.0 (3.9, 30.3)	279	10.0 (3.6, 26.8)	276	-	0.57
**12 months**	13.3 (5.9, 31.1)	280	11.7 (3.4, 31.1)	276	-	0.14
Contralateral leg SLS average*[b] *(seconds)						
**Baseline**	7.2 (3.6, 22.0)	310	6.3 (2.6, 18.0)	308	-	-
**6 months**	10.9 (4.2, 30.8)	279	9.8 (3.5, 29.7)	276	-	0.43
**12 months**	14.7 (5.6, 30.7)	280	11.0 (3.2, 29.7)	276	-	0.03

Notes: 30SCST = 30 Seconds Chair Stand Test, which records the number of stands in 30 seconds and where higher scores indicate better function. F8WT = Figure of 8 Walk Test, where a quicker time and fewer steps indicates better function. SLS = Single Leg Stance, in which stance time is averaged over three trials at standing on the same leg, with a maximum score of 45 seconds and higher scores indicate better function

[a] *Summaries are mean (SD)*

[b] *Summaries are median (IQR)*

For peer review only

Table 4: SOC codes of SAEs by treatment group

	Usual Care (n= 14 (4.5%))	Home-based Rehab (n= 18 5.8%)
**Blood/lymphatic**	2	2
**Cardiac**	2	1
**Endocrine**	1	0
**Gastrointestinal**	2	2
**Immune system**	0	1
**Infections/infestations**	1	3
**Musculoskeletal**	1	6
**Nervous system**	0	1
**Renal/urinary**	2	0
**Respiratory/thoracic**	0	1
**Skin**	1	0
**Social circumstances**	1	0
**Vascular**	1	0
**Unknown**		1

For peer review only

Figure 1 - CONSORT flow chart

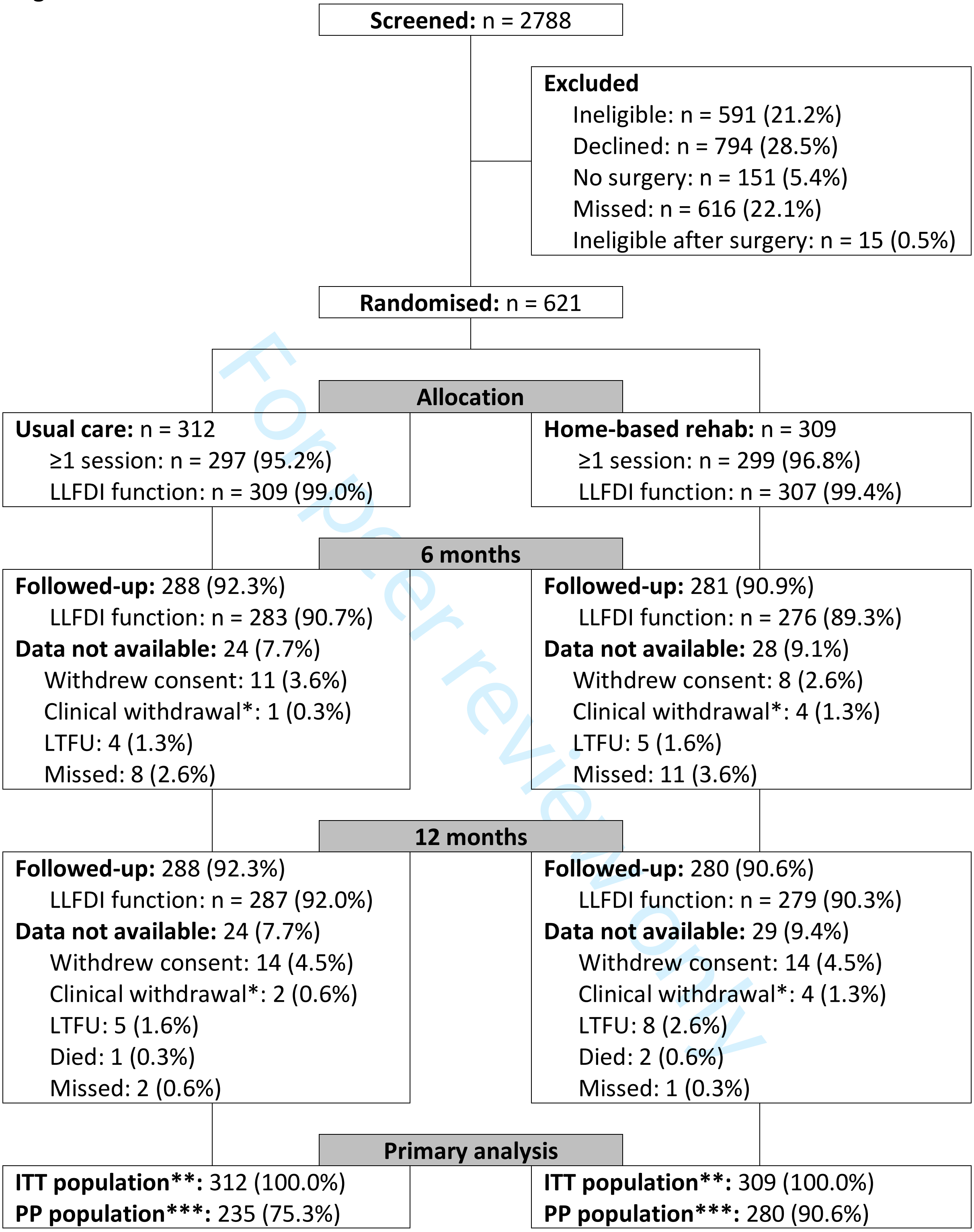

**Clinical withdrawals include those who withdrew due to an adverse event, serious adverse event, or medical contraindication*

**Missing follow-up data in the intent-to-treat (ITT) population was imputed using multiple imputation for the primary outcome analysis.*

***Analysis of the per-protocol (PP) population was based on available data only.*

LLFDI: Late Life Function and Disability Instrument; LTFU: lost to follow-up

For peer review only

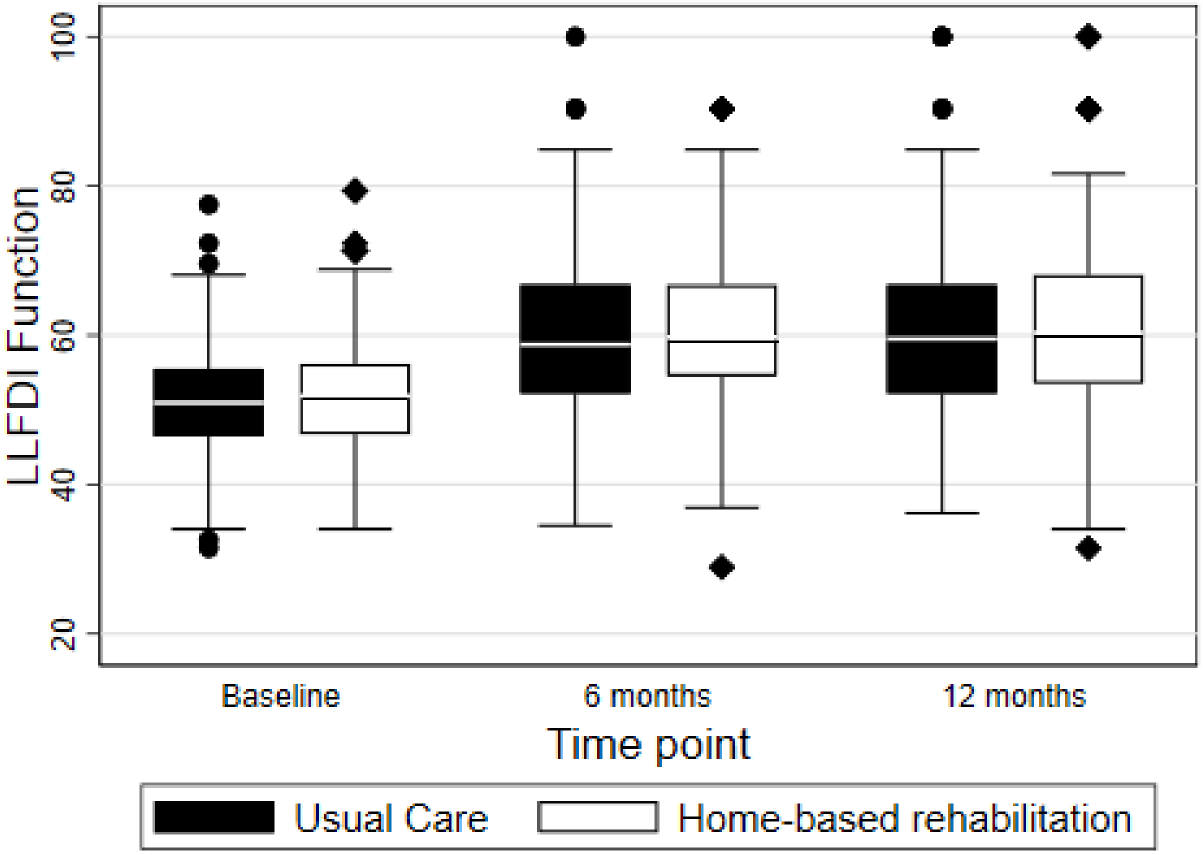

For peer review only - http://bmjopen.bmj.com/site/about/guidelines.xhtml

Corka

CORKA Screening Tool

Study code:	Site ID code:	Participants Study Number*:	Initials:
C K –	–		

* Add in once Participant has been randomised

DOB: _ _/_ _ _/_ _ _ _ **Principal Investigator:** ________________ Date _ _/_ _ _/_ _ _ _

Participant Screening Number (number given when participant originally screened) CSN __ __ __ __

From pre-admission clinic, complete the following:

	**SCORE** *(Please Circle)*
1. What is the participant's Body Mass Index (BMI)?	
Normal weight ($<25kg/m^2$)	**0**
Overweight ($25\text{-}29.9kg/m^2$)	**1**
Obese ($\geq 30kg/m^2$)	**2**
2. From Q1 of the Oxford Knee Score (OKS) questionnaire; *'How would you describe the pain you usually have from your knee?'*	
None OR very mild OR mild	**0**
Moderate OR severe	**4**
3. What is the participant's health status?	
Fit and healthy with mild systemic disease i.e. controlled hypertension/diabetes	**0**
Severe systemic disease i.e. unstable coronary heart disease	**2**
4. In the **past four weeks** have you been limited in the kind of work you do or other regular activities you carry out as a result of feeling anxious or depressed?	
None of the time OR a little of the time OR some of the time	**0**
Most of the time OR all of the time	**2**
**TOTAL:**	

For patients with a score of 5 or more, continue to screen for eligibility onto the study.

For patients with a score below 5, stop the screening process and record this in the screening log.

For peer review only - http://bmjopen.bmj.com/site/about/guidelines.xhtml

Appendix B – content of treatment Interventions

The CORKA home-based intervention was a multicomponent rehabilitation programme delivered to participants in their own home by a mixture of qualified staff and rehabilitation assistants. Its main component was an exercise programme, but it also included functional task practice, adherence approaches and provision of appropriate aids or equipment. It consisted of an initial appointment and 6 follow up sessions.

Initial appointment – assessment

The subjective assessment included their current symptoms and the perceived level of pain or discomfort on a VAS scale from 0-10 with 0 being 'no symptoms' and 10 the 'worse imaginable symptoms', their social history, any medication taken, their history of falls and their activities of daily living (ADLs).

The objective assessment documented the participant's wound, the range of movement in the operated and non-operated knees, strength of quadriceps, hamstrings, hip abductors and the calf muscles, scored on an Oxford grading scale. Standing balance was assessed by observing and recording the ability to stand with feet side by side, or in a semi-tandem or tandem stance. An assessment was made of gait and if appropriate ability to ascend and descend stairs. If deemed necessary and safe any transfers were assessed.

Finally, observation of at least one functional task took place which was identified by the participant as being potentially problematic. The exercise programme was subsequently tailored to meet the specific needs, goals and functional problems of the individual.

Initial appointment – exercise programme

Following the assessment the physiotherapist and rehabilitation assistant set each participant an exercise programme, which would replace any post-operative programme given on discharge from hospital. It would consist of exercises categorised into the following sections :

1. Knee range of motion - flexion
2. Knee range of motion – extension
3. Basic quadriceps strengthening
4. Strengthening exercises – quadriceps
5. Strengthening exercises – hamstrings
6. Strengthening exercises – hip abductors
7. Strengthening exercises – calf
8. Static balance exercises
9. Dynamic balance and walking skills exercises

The selection of each specific exercise took place as follows, for ROM exercises the physiotherapist selected an exercise and then checked that they and the participant were happy with how it was performed. If this was the case the participant was asked to perform 3 sets of 10-15 repetitions, unless a stretch had been selected, when they were asked to undertake 3 sets of 3-5 repetitions with a 30 to 60 second hold.

For strengthening exercises the participant was given an exercise that they could perform for 6-12 repetitions with good form at a moderate intensity. Perceived intensity was measured using the 10 point Rating of Perceived Exertion (RPE) scale (Borg 1998). The participant was given an exercise

For peer review only

where they feel they were working at RPE of 3-4 (moderate to somewhat hard). Participants were asked to complete 1 to 3 sets of 6-12 reps of strengthening exercises.

For the balance section, participants were prescribed an exercise that was sufficiently challenging without putting the participant at risk of falling. Participants were asked to perform 3 repetitions of the exercise with a 30 second hold.

For the final section, gait skills there were a variety of exercises to choose from. The physiotherapist was asked to prescribe an exercise that was sufficiently challenging without putting the participants at risk of falling. The exact number of sets and reps were determined based on the particular exercise selected and the individual patient.

During session 3 a graduated walking programme was introduced. Participants were asked about the maximum time or distance that they have managed to walk since surgery and were encouraged to increase this distance or time gradually in small increments.

Participants were also asked to practice between 1-3 specific functional tasks highlighted in the initial assessment.

CORKA intervention exercise prescription time points

	Range of Movement	Basic Quads strength	Strengthening	Balance	Gait Skills	Task practice	Walking programme
Session 1	✓	✓	✓	✓	✓	✓	
Session 2	✗	✗	⇧	⇧	⇧	⇧	
Session 3	✗	✗	⇧	⇧	⇧	⇧	✓
Session 4	✗	✗	⇧	⇧	⇧	⇧	⇧
Session 5	✗	✗	⇧	⇧	⇧	⇧	⇧
Session 6	✗	✗	⇧	⇧	⇧	⇧	⇧
Session 7	✗	✗	⇧	⇧	⇧	⇧	⇧

✓ = issue exercise

✗ = Stop exercise once appropriate level of function reached

⇧ = Progress exercise

For peer review only

CORKA Intervention Exercises Table

Exercise	Level	Purpose	Functional relevance
Hip and knee flexion 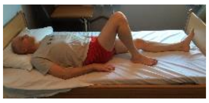	1A	Lower limb range of movement	Walking, stair climbing, kneeling
Passive knee flexion 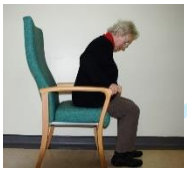	1B	Lower limb range of movement	Walking, stair climbing, sitting and standing up from a chair, kneeling
Active assisted knee flexion 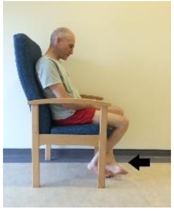	1C	Lower limb range of movement	Walking, stair climbing, sitting and standing up from a chair, kneeling
Hold-relax knee flexion 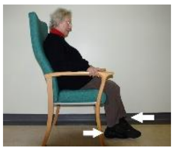	1D	Lower limb range of movement	Walking, stair climbing, sitting and standing up from a chair, kneeling
Active Knee flexion 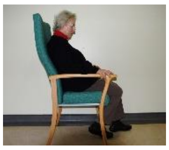	1E	Lower limb range of movement	Walking, stair climbing, sitting and standing up from a chair, kneeling
Hip and knee flexion with overpressure 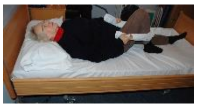	1F	Lower limb range of movement	Walking, stair climbing, kneeling
Knee extension stretch 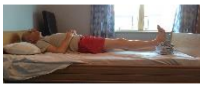	2A	Lower limb range of movement	Walking, stair climbing, standing up from a chair

For peer review only

Knee extension with overpressure 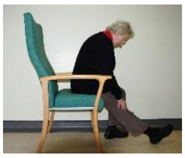	2B	Lower limb range of movement	Walking, stair climbing, standing up from a chair
Knee extension 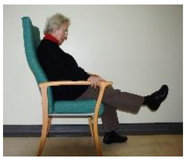	2C	Lower limb range of movement	Walking, stair climbing, standing up from a chair, dressing, getting in/out of car
Hamstring stretch 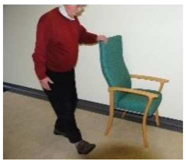	2D	Lower limb range of movement	Walking, stair climbing, dressing
Calf stretch 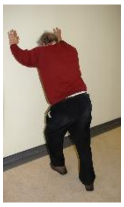	2E	Lower limb range of movement	Walking, stair climbing, dressing
Static Quadriceps 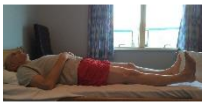	3A	Lower limb strength	Walking, stair climbing, standing up from a chair
Inner range quadriceps 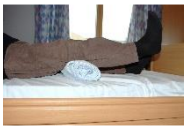	3B	Lower limb strength	Walking, stair climbing, standing up from a chair
Long arc quadriceps 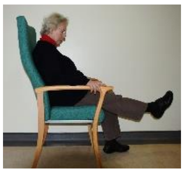	4A	Lower limb strength	Walking, stair climbing, standing up from a chair

For peer review only

Knee extension with ankle weight 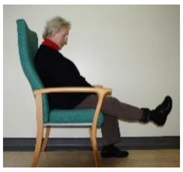	4B	Lower limb strength	Walking, stair climbing, standing up from a chair
Sit to stand using arms 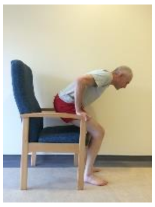	4C	Upper and lower body strength	Walking, stair climbing, standing up from a chair, lifting/carrying household objects, getting in/out of car
Sit to stand without arms 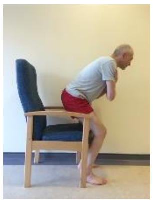	4D	Lower limb and trunk strength	Stair climbing, standing up from a chair, walking, getting in/out of car
Wall slide 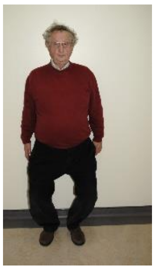	4E	Lower limb strength	Stair climbing, standing up from a chair, walking, getting in/out of car, lifting household objects
Mini Squats 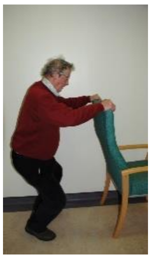	4F	Lower limb strength	Stair climbing, standing up from a chair, walking, getting in/out of car, lifting household objects

For peer review only

Single leg dip 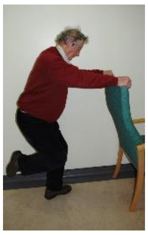	4G	Lower limb strength	Stair climbing, standing up from a chair, walking, lifting household objects
Static hamstrings 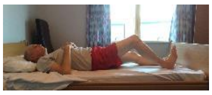	5A	Lower limb strength	Stair climbing, standing up from a chair, walking, lifting household objects
Sliding heel backwards 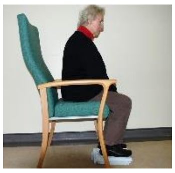	5B	Lower limb strength and range of movement	Stair climbing, standing up from a chair, walking, kneeling
Standing hamstring curls 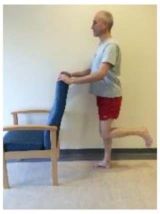	5C	Lower limb strength	Stair climbing, standing up from a chair, walking, lifting household objects
Active hamstrings 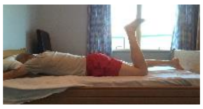	5D	Lower limb strength	Stair climbing, standing up from a chair, walking, lifting household objects
Standing hamstring curls with ankle weight 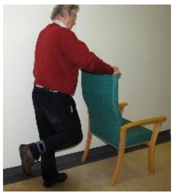	5E	Lower limb strength	Stair climbing, standing up from a chair, walking, lifting household objects
Lunge 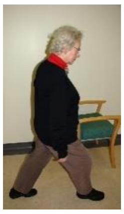	5F	Lower limb strength	Stair climbing, standing up from a chair, walking, lifting household objects

For peer review only

Hip abduction 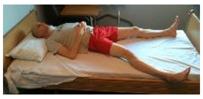	6A	Lower limb strength	Walking, dressing, getting into/out of bed
Bridging 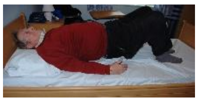	6B	Lower limb strength	Dressing, walking , standing up from a chair
Hip hitching 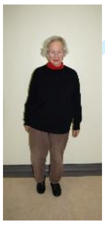	6C	Lower limb strength	Walking, dressing, getting into/out of bed
Standing hip abduction 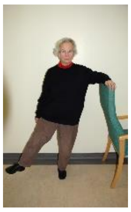	6D	Lower limb strength	Walking, dressing, getting into/out of bed, standing from a chair
Standing hip abduction with ankle weight 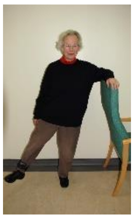	6E	Lower limb strength	Walking, dressing, getting into/out of bed, standing from a chair
Short lever hip abduction in side lying 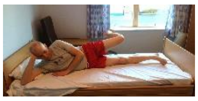	6F	Lower limb strength	Walking, dressing, getting into/out of bed
Long lever hip abduction in side lying 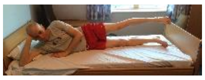	6G	Lower limb strength	Walking, dressing, getting into/out of bed
Hip abduction in side lying with ankle weight 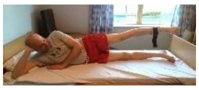	6H	Lower limb strength	Walking, dressing, getting into/out of bed

Bilateral calf raise 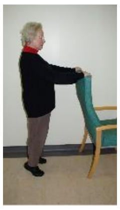	7A	Lower limb strength	Walking and stair climbing
Single calf raise 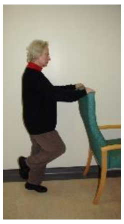	7B	Lower limb strength	Walking and stair climbing
Standing feet together 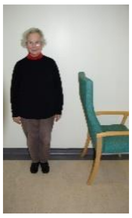	8A	Balance	Falls Prevention
Semi-tandem stance 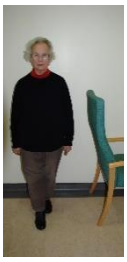	8B	Balance	Falls prevention
Tandem stance 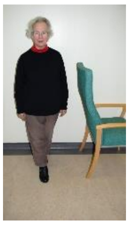	8C	Balance	Falls Prevention
Single leg stance 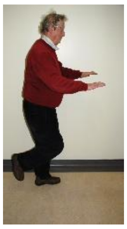	8D	Balance	Falls Prevention
Walking on the spot 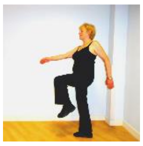	9A	Walking skills and balance	Walking and falls prevention

For peer review only

Waling forwards and backwards 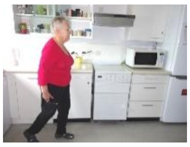	9B	Walking skills and balance	Walking a straight path and falls prevention
Side stepping 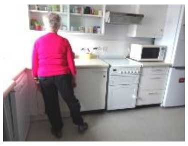	9C	Walking skills and balance	Walking, stepping around obstacles, falls prevention
Step ups 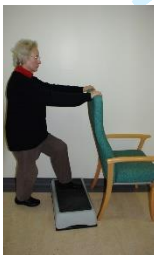	9D	Walking skills and balance	Walking, stepping onto kurbs, stair climbing, falls prevention
Mini lunge step up 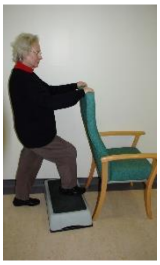	9E	Walking skills and balance	Walking, stepping onto kurbs, stair climbing, falls prevention
Turning 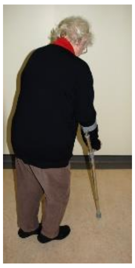	9F	Walking skills and balance	Walking with aid and falls prevention
Figure of 8 walk 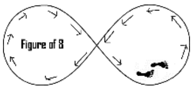	9G	Walking skills and balance	Walking both straight and curved paths, falls prevention

For peer review only

Tandem walking 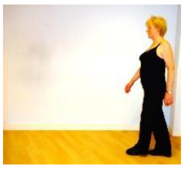	9H	Walking skills and balance	Walking and falls prevention

Usual Care

Participants randomised to usual care underwent standard in and out-patient post-operative physiotherapy as it was delivered routinely at each of the 14 research sites.

On discharge participants were given general written advice following knee arthroplasty and a home exercise programme to continue with until they were seen by an out-patient physiotherapist. To standardise usual care, participants would receive at least 1 session of physiotherapy and up to a maximum of 6 sessions which aligns with what the NHS can realistically provide. The exact duration and content of the physiotherapy sessions were at the treating physiotherapist's discretion, but in general would be between 30 and 60 minutes duration and would include a combination of range of motion and strengthening exercises, gait re-education with progression of walking aids and advice. Any differences seen in usual care across the sites is reflective of the variety of standard care delivered post knee arthroplasty across the UK.

For peer review only

CONSORT 2010 checklist of information to include when reporting a randomised trial*

Section/Topic	Item No	Checklist item	Reported on page No
**Title and abstract**			
	1a	Identification as a randomised trial in the title	1
	1b	Structured summary of trial design, methods, results, and conclusions (for specific guidance see CONSORT for abstracts)	1-2
**Introduction**			
Background and objectives	2a	Scientific background and explanation of rationale	3-4
	2b	Specific objectives or hypotheses	1 & 4
**Methods**			
Trial design	3a	Description of trial design (such as parallel, factorial) including allocation ratio	4-8
	3b	Important changes to methods after trial commencement (such as eligibility criteria), with reasons	n/a
Participants	4a	Eligibility criteria for participants	5
	4b	Settings and locations where the data were collected	6
Interventions	5	The interventions for each group with sufficient details to allow replication, including how and when they were actually administered	6
Outcomes	6a	Completely defined pre-specified primary and secondary outcome measures, including how and when they were assessed	7
	6b	Any changes to trial outcomes after the trial commenced, with reasons	n/a
Sample size	7a	How sample size was determined	7
	7b	When applicable, explanation of any interim analyses and stopping guidelines	n/a
Randomisation:			
Sequence generation	8a	Method used to generate the random allocation sequence	5
	8b	Type of randomisation; details of any restriction (such as blocking and block size)	5
Allocation concealment mechanism	9	Mechanism used to implement the random allocation sequence (such as sequentially numbered containers), describing any steps taken to conceal the sequence until interventions were assigned	5
Implementation	10	Who generated the random allocation sequence, who enrolled participants, and who assigned participants to interventions	5
Blinding	11a	If done, who was blinded after assignment to interventions (for example, participants, care providers, those	5

For peer review only

		assessing outcomes) and how	
	11b	If relevant, description of the similarity of interventions	5-6
Statistical methods	12a	Statistical methods used to compare groups for primary and secondary outcomes	6-8
	12b	Methods for additional analyses, such as subgroup analyses and adjusted analyses	6-8
**Results**			
Participant flow (a diagram is strongly recommended)	13a	For each group, the numbers of participants who were randomly assigned, received intended treatment, and were analysed for the primary outcome	9,22
	13b	For each group, losses and exclusions after randomisation, together with reasons	9,22
Recruitment	14a	Dates defining the periods of recruitment and follow-up	6
	14b	Why the trial ended or was stopped	n/a
Baseline data	15	A table showing baseline demographic and clinical characteristics for each group	Table 1
Numbers analysed	16	For each group, number of participants (denominator) included in each analysis and whether the analysis was by original assigned groups	9 & 24
Outcomes and estimation	17a	For each primary and secondary outcome, results for each group, and the estimated effect size and its precision (such as 95% confidence interval)	27-29
	17b	For binary outcomes, presentation of both absolute and relative effect sizes is recommended	n/a
Ancillary analyses	18	Results of any other analyses performed, including subgroup analyses and adjusted analyses, distinguishing pre-specified from exploratory	9
Harms	19	All important harms or unintended effects in each group (for specific guidance see CONSORT for harms)	11,30
**Discussion**			
Limitations	20	Trial limitations, addressing sources of potential bias, imprecision, and, if relevant, multiplicity of analyses	12
Generalisability	21	Generalisability (external validity, applicability) of the trial findings	11-13
Interpretation	22	Interpretation consistent with results, balancing benefits and harms, and considering other relevant evidence	11-13
**Other information**			
Registration	23	Registration number and name of trial registry	2
Protocol	24	Where the full trial protocol can be accessed, if available	4
Funding	25	Sources of funding and other support (such as supply of drugs), role of funders	2

*We strongly recommend reading this statement in conjunction with the CONSORT 2010 Explanation and Elaboration for important clarifications on all the items. If relevant, we also recommend reading CONSORT extensions for cluster randomised trials, non-inferiority and equivalence trials, non-pharmacological treatments, herbal interventions, and pragmatic trials. Additional extensions are forthcoming: for those and for up to date references relevant to this checklist, see www.consort-statement.org.

For peer review only - http://bmjopen.bmj.com/site/about/guidelines.xhtml

For peer review only

TIDieR
Template for Intervention Description and Replication

The TIDieR (Template for Intervention Description and Replication) Checklist*:

Information to include when describing an intervention and the location of the information

Item number	Item	Where located ** Primary paper (page or appendix number)	Other † (details)
	**BRIEF NAME**		
**1.**	Provide the name or a phrase that describes the intervention.	P5 lines 126-136	Paper Ref 13
	**WHY**		
**2.**	Describe any rationale, theory, or goal of the elements essential to the intervention.	_P3 l83-89	_Ref 13 & 22_
	**WHAT**		
**3.**	Materials: Describe any physical or informational materials used in the intervention, including those provided to participants or used in intervention delivery or in training of intervention providers. Provide information on where the materials can be accessed (e.g. online appendix, URL).	______________	_Ref 13 & 22_
**4.**	Procedures: Describe each of the procedures, activities, and/or processes used in the intervention, including any enabling or support activities.	______________	__Ref 13 & 22_
	**WHO PROVIDED**		
**5.**	For each category of intervention provider (e.g. psychologist, nursing assistant), describe their expertise, background and any specific training given.	_P5 l 135-136_	______________
	**HOW**		
**6.**	Describe the modes of delivery (e.g. face-to-face or by some other mechanism, such as internet or telephone) of the intervention and whether it was provided individually or in a group.	_p5 l 126-146__	______________
	**WHERE**		
**7.**	Describe the type(s) of location(s) where the intervention occurred, including any necessary infrastructure or relevant features.	__p5 126-146_	______________

For peer review only - http://bmjopen.bmj.com/site/about/guidelines.xhtml

	**WHEN and HOW MUCH**		
**8.**	Describe the number of times the intervention was delivered and over what period of time including the number of sessions, their schedule, and their duration, intensity or dose.	_p5 l133-135	______________
	**TAILORING**		
**9.**	If the intervention was planned to be personalised, titrated or adapted, then describe what, why, when, and how.	______________	__Ref 13 & 22_
	**MODIFICATIONS**		
**10.‡**	If the intervention was modified during the course of the study, describe the changes (what, why, when, and how).	________n/a___	______________
	**HOW WELL**		
**11.**	Planned: If intervention adherence or fidelity was assessed, describe how and by whom, and if any strategies were used to maintain or improve fidelity, describe them.	______________	_Ref 11 & 22_
**12.‡**	Actual: If intervention adherence or fidelity was assessed, describe the extent to which the intervention was delivered as planned.	______________	_Ref 22

For peer review only

** **Authors** - use N/A if an item is not applicable for the intervention being described. **Reviewers** – use '?' if information about the element is not reported/not sufficiently reported.

† If the information is not provided in the primary paper, give details of where this information is available. This may include locations such as a published protocol or other published papers (provide citation details) or a website (provide the URL).

‡ If completing the TIDieR checklist for a protocol, these items are not relevant to the protocol and cannot be described until the study is complete.

* We strongly recommend using this checklist in conjunction with the TIDieR guide (see *BMJ* 2014;348:g1687) which contains an explanation and elaboration for each item.

* The focus of TIDieR is on reporting details of the intervention elements (and where relevant, comparison elements) of a study. Other elements and methodological features of studies are covered by other reporting statements and checklists and have not been duplicated as part of the TIDieR checklist. When a **randomised trial** is being reported, the TIDieR checklist should be used in conjunction with the CONSORT statement (see www.consort-statement.org) as an extension of **Item 5 of the CONSORT 2010 Statement.** When a **clinical trial protocol** is being reported, the TIDieR checklist should be used in conjunction with the SPIRIT statement as an extension of **Item 11 of the SPIRIT 2013 Statement** (see www.spirit-statement.org). For alternate study designs, TIDieR can be used in conjunction with the appropriate checklist for that study design (see www.equator-network.org).

For peer review only - http://bmjopen.bmj.com/site/about/guidelines.xhtml

BMJ Open

Does a home-based rehabilitation programme for those at risk of poor outcome after knee arthroplasty offer superior outcomes compared to traditional physiotherapy? The CORKA randomised controlled trial.

Journal:	*BMJ Open*
Manuscript ID	bmjopen-2021-052598.R1
Article Type:	Original research
Date Submitted by the Author:	21-Jul-2021
Complete List of Authors:	Barker, Karen L.; University of Oxford, Nuffield Department of Orthopaedics, Rheumatology and Musculoskeletal Sciences<br>Room, Jonathan; Oxford University Hospitals NHS Foundation Trust Nuffield Orthopaedic Centre<br>Knight, Ruth; University of Oxford, Oxford Clinicial Trials Research Unit (OCTRU); Centre Statistics in Medicine<br>Dutton, Susan; University of Oxford, Oxford Clinical Trials Research Unit (OCTRU); Centre statistics in Medicine<br>Toye, Francine; Oxford University Hospitals NHS Foundation Trust Nuffield Orthopaedic Centre, Nuffield Orthopaedic Centre Physiotherapy Research Unit<br>Leal, Jose; University of Oxford, Nuffield Department of Population Health<br>Kenealy, Nicola; Oxford University, Nuffield Department of Orthopaedics, Rheumatology and Musculoskeletal Sciences<br>Maia Schlüssel, Michael; University of Oxford, Nuffield Department of Orthopaedics, Rheumatology and Musculoskeletal Sciences<br>Collins, Gary; University of Oxford, Centre for Statistics in Medicine<br>Beard, David; University of Oxford, Nuffield Dept of Orthopaedics, Rheumatology and Musculoskeletal Sciences<br>Price, Andrew; University of Oxford, Nuffield Department of Orthopaedics, Rheumatology and Musculoskeletal Sciences<br>Underwood, Martin; Warwick University, Warwick Medical School<br>Drummond, Avril; University of Nottingham, Div of Rehabilitation and Ageing<br>Lamb, Sarah; University of Exeter, College of Medicine and Health; University of Oxford, NDORMS
**Primary Subject Heading**:	Evidence based practice
Secondary Subject Heading:	Rehabilitation medicine
Keywords:	Adult orthopaedics < ORTHOPAEDIC & TRAUMA SURGERY, Knee < ORTHOPAEDIC & TRAUMA SURGERY, REHABILITATION MEDICINE

I, the Submitting Author has the right to grant and does grant on behalf of all authors of the Work (as defined in the below author licence), an exclusive licence and/or a non-exclusive licence for contributions from authors who are: i) UK Crown employees; ii) where BMJ has agreed a CC-BY licence shall apply, and/or iii) in accordance with the terms applicable for US Federal Government officers or employees acting as part of their official duties; on a worldwide, perpetual, irrevocable, royalty-free basis to BMJ Publishing Group Ltd ("BMJ") its licensees and where the relevant Journal is co-owned by BMJ to the co-owners of the Journal, to publish the Work in this journal and any other BMJ products and to exploit all rights, as set out in our licence.

The Submitting Author accepts and understands that any supply made under these terms is made by BMJ to the Submitting Author unless you are acting as an employee on behalf of your employer or a postgraduate student of an affiliated institution which is paying any applicable article publishing charge ("APC") for Open Access articles. Where the Submitting Author wishes to make the Work available on an Open Access basis (and intends to pay the relevant APC), the terms of reuse of such Open Access shall be governed by a Creative Commons licence – details of these licences and which Creative Commons licence will apply to this Work are set out in our licence referred to above.

Other than as permitted in any relevant BMJ Author's Self Archiving Policies, I confirm this Work has not been accepted for publication elsewhere, is not being considered for publication elsewhere and does not duplicate material already published. I confirm all authors consent to publication of this Work and authorise the granting of this licence.

For peer review only

Does a home-based rehabilitation programme for those at risk of poor outcome after knee arthroplasty offer superior outcomes compared to traditional physiotherapy? The CORKA randomised controlled trial.

Karen L. Barker [1,2] *, Jon Room[1,2], Ruth Knight[3], Susan J. Dutton[3], Fran Toye[2], Jose Leal[4], Nicola Kenealy[1], Michael Maia Schussel[3], Gary Collins[3], David J. Beard[1], Andrew Price[1], Martin Underwood[5], Avril Drummond[6], Sarah E Lamb[1,7,] on behalf of CORKA Trial group.

1 Nuffield Department of Orthopaedics, Rheumatology and Musculoskeletal Sciences, University of Oxford, Oxford, UK.

2 Physiotherapy Research Unit, Nuffield Orthopaedic Centre, Oxford University Hospitals NHS Foundation Trust, Oxford, UK.

3 Oxford Clinical Trials Research Unit (OCTRU), Centre for Statistics in Medicine, Nuffield Department of Orthopaedics, Rheumatology and Musculoskeletal Sciences, University of Oxford, Oxford, UK.

4 Health Economics Research Centre, Nuffield Department of Population Health, University of Oxford, Oxford, UK.

5 Warwick Clinical Trials Unit, Warwick Medical School, University of Warwick, Coventry, UK

6 School of Health Sciences, University of Nottingham, Nottingham, UK

7 School of Medicine and Health, University of Exeter

*Corresponding Author Email karen.barker@ouh.nhs.uk.

Physiotherapy Research Unit

Nuffield Orthopaedic Centre

Windmill Rd, Oxford OX3 7LD

Tel no: 01865 738080

Keywords: knee arthroplasty, physical therapy, rehabilitation

For peer review only

Word Count: 4143

Abstract

Objectives: To evaluate whether a home-based rehabilitation programme for people assessed as being at risk of a poor outcome after knee arthroplasty offers superior outcomes to traditional out-patient physiotherapy.

Design: A prospective, single-blind, two-arm randomised controlled superiority trial.

Setting: 14 National Health Service (NHS) physiotherapy departments in the UK.

Participants: 621 participants identified at high risk of a poor outcome after knee arthroplasty using a bespoke screening tool.

Interventions: A multi-component home-based rehabilitation programme delivered by rehabilitation assistants with supervision from qualified therapists versus usual care out-patient physiotherapy.

Main outcome measures: The primary outcome was the Late Life Function and Disability Instrument (LLFDI) at 12 months. Secondary outcomes were the Oxford Knee Score (OKS; a disease-specific measure of function), Knee injury and Osteoarthritis Outcome Score (KOOS) Quality of Life subscale, Physical Activity Scale for the Elderly (PASE), EQ-5D-5L, and physical function assessed using the Figure of 8 Walk test, 30 Second Chair Stand Test (30SCST), and Single Leg Stance.

Results: 621 participants were randomised between March 2015 and January 2018. 309 were assigned to CORKA home-based rehabilitation, receiving a median 5 treatment sessions (IQR 4-7). 312 were assigned to usual care, receiving a median 4 sessions (IQR 2-6). The primary outcome, LLFDI function total score at 12 months, was collected for 279 participants (89%) in the home-based CORKA group and 287 participants (92%) in the usual-care group. No clinically or statistically significant difference was found between the groups (intention-to-treat adjusted

For peer review only

difference = 0.49 points; 95% confidence interval -0.89 to 1.88; p = 0.48). There were no statistically significant differences between the groups on any of the patient-reported or physical secondary outcome measures at 6 or 12 months.

There were 18 participants in the intervention group reporting a serious adverse event (5.8%), only one directly related to the intervention., all other adverse events recorded throughout the trial related to underlying chronic medical conditions.

Conclusions: The CORKA intervention was not superior to usual care. The trial detected no significant differences, clinical or statistical, between the two groups on either primary or secondary outcomes. CORKA offers an evaluation of an intervention utilising a different service delivery model for this patient group.

Trial Registration: ISRCTN 13517704

Funding: National Institute for Health Research Health Technology Assessment programme (HTA 12/196/08)

Article Summary

Strengths and limitations of this study:

- Large sample size and low loss to follow up which ensured sufficient numbers to make the study sufficiently powered to detect any between group differences.
- The trial was pragmatic and utilised an innovative workforce model that could readily translate into NHS practice within existing commissioning and financial constraints.
- The study offers an alternative service delivery model in line with the latest UK guidance from NICE.
- Limitations are that the screening tool did not identify patients that were a good fit of risk of poor outcome for the intervention that we developed.
- Another limitation, common to all trials of post operative physiotherapy after knee arthroplasty was the absence of a no physiotherapy treatment control group. As post

For peer review only

discharge out-patient physiotherapy remains an accepted and expected core component of UK arthroplasty practice it was not deemed ethical or practicable to attempt to recruit to a no treatment comparator in a group who had already been identified as at higher risk of a poor outcome.

Introduction

Knee Arthroplasty operations in the UK continue to rise with the National Joint Registry reporting over 100,000 in 2017 alone (1). This is an increase of over 3% from 2010. The population receiving surgery is getting older, frailer and with more co-existing health conditions. Data from patient-reported outcome measures (PROMs) have shown that most patients achieve a satisfactory outcome. However, between 10% and 15% are unsatisfied or report little or no improvement after surgery (2). We do not yet know how to identify these patients or target rehabilitation to improve their outcomes.

Physiotherapy provision in the UK varies following discharge from hospital after KA but typically patients will continue to have rehabilitation needs to help restore muscle strength and endurance, range of motion, walking distance, and performance of higher-level functional activities. Patients are commonly referred for further physiotherapy in an out-patient setting to assist with these issues. The timing of this referral varies, with some hospitals advocating immediate commencement of therapy after discharge from hospital and others waiting until the first post-operative review at around 6 weeks before considering referral. This typically involves between 4 and 6 sessions of physiotherapy in an out-patient clinic setting. Patients do not routinely receive Occupational Therapy input following knee arthroplasty surgery unless they are highlighted to have particular difficulties with their daily activities or have specific barriers to discharge home. Concern has been raised that many exercise programmes lack adequate intensity to enable optimal recovery (3,4). Internationally, where much longer courses of physiotherapy are often provided, research has indicated that 12-18 hours of physiotherapy (5) or a mean of 17 visits (6) may be needed to produce a benefit. These levels of

For peer review only

care are substantially higher than those provided in the UK and, in the current economic climate, may be more than the National Health Service (NHS) can afford.

Given the rising number of operations, the relatively limited therapy resources available, and the increasing age and frailty of patients receiving this surgery, it is important that rehabilitation resources are concentrated on those patients who need the most help to achieve a good outcome. Not all patients need post-operative out-patient physiotherapy and some will make a full recovery using self-directed rehabilitation (7–10). Current rehabilitation strategies do not meet the needs of all patients, particularly those who are socially isolated, do not have easy access to transport, and are frail (11). It is thus pertinent to focus our resources on those who are most likely to be at risk of a poor outcome, least likely to be able to engage with a self-management approach, and able to benefit most from rehabilitation input To maximise access to therapy and to provide this within a similar cost envelope to current practice we designed an intervention to be delivered in the patients' homes utilising a workforce model that utilise rehabilitation assistants supported by qualified therapy staff, drawing on the success of this model in delivering therapy programmes such as the OTAGO falls prevention intervention (12). This trial was carried out to determine if a targeted home-based intervention for those at risk of poor outcome, delivered by a mix of qualified staff and rehabilitation assistants, offers superior outcomes in patient reported and physical function at 1 year following KA compared to traditional out-patient physiotherapy.

Methods

The CORKA trial was a multicentre prospective, single blinded, two-arm randomised controlled superiority trial with assessment for the clinical outcomes at baseline, 6 and 12 months. The study was carried out according to the published protocol (13).

Recruitment

Participants were recruited from 14 NHS Trusts across England. Patients aged 55 years and over due to undergo knee arthroplasty were sent information concerning the trial prior to their pre-operative assessment appointment. Those expressing interest were screened in the clinic using

For peer review only

a screening tool developed for the trial. This aimed to identify patients at risk of a poorer outcome based on data collected as part of standard pre-operative assessment screening. The tool was developed using all data from the Knee Arthroplasty Trial dataset and further details of its development has been previously published (14). The screening tool had a range of possible scores from 0 – 10, patients with a score of 5 or more were classified as being at increased risk of a poor outcome after 1 year defined as an Oxford Knee Score of 26 or less. [Appendix A]. The performance of the model was fair, as measured by its discrimination. Participants who were eligible were screened again post-operatively to confirm their eligibility and consent. All participants provided written informed consent. Exclusion criteria included any major peri-operative complications, further surgery planned within the next 12 months and absolute contraindications to exercise. Screening was performed by research clinicians at the local sites, and screening logs recorded the reasons for ineligibility and why eligible patients declined to participate.

Randomisation

Randomisation took place on the third post-operative day, or on hospital discharge if earlier. Should any participant have experienced a severe medical peri-operative event they would not be entered into the trial but continue with usual care as able. Participants were randomly allocated by a web-based randomisation system to either 'usual care' or 'home-based rehabilitation programme' in a 1:1 ratio using permuted blocks of various sizes (sizes 2, 4 and 6 in a 1:2:1 ratio, that is on average there were twice as many blocks of size 4 than either size 2 or size 6) to prevent prediction of treatment allocation, and this was stratified by recruitment site. Once randomised, participants and those delivering the rehabilitation were aware of the treatment allocation due to the nature of the intervention. Those carrying out follow-up outcome measurements remained blinded to treatment allocation.

Interventions

<u>CORKA home-based intervention</u>

For peer review only

The CORKA intervention started up to 4 weeks after surgery, with most participants seen within 2 weeks. In the period between surgery and the initial appointment participants continued with a home exercise programme given to them by ward physiotherapists in line with standard current practice.

The CORKA home-based intervention is a multicomponent rehabilitation programme developed in collaboration with clinicians and patients; it has been described in detail in a previous publication (15). It consisted of an initial assessment appointment and up to 6 follow-up sessions. Its aim was to improve the function and participation in activities of participants at risk of a poor outcome after surgery. The primary component of the rehabilitation programme was an individually adapted exercise package conducted in participants' own homes. During the participants' initial assessment, the physiotherapist focused on tasks that were identified as being problematic. The exercise programme was then tailored to the individuals' needs and goals. If an exercise was felt to be unsafe for any particular participant, therapists could omit it and substitute with an additional exercise from the package; or utilise a more stable starting position. Additional components consisted of functional task practice, adherence approaches such as utilisation of techniques such as goal setting and exercise dairies. If required, they were provided with appropriate aids and equipment,

The physiotherapist undertook the initial assessment and prescription of the exercise programme, observed by the rehabilitation assistant. For future sessions the rehabilitation assistant continued the programme and modified as required using treatment algorithms and decision aids to progress. The physiotherapist undertook one further session in the middle of the programme to review the participant's progress and their exercise programme. The way this was operationalised is outlined in Appendix B.

All therapists delivering the CORKA intervention received a 2-3 hour training session which included instructions on how to assess and treat CORKA participants, prescribe and progress the different categories within the exercise programme.

<u>Usual care</u>

Those allocated to the usual-care arm received standard post-operative physiotherapy. Usual care after surgery could vary considerably across the trial's different UK locations. However, it

For peer review only

was highly likely that usual care would include several of the following: between 1 and 6 sessions of physiotherapy in an out-patient setting, class-based setting, or hydrotherapy; written advice on home exercises on discharge from hospital; and an assessment of any potential home requirements or barriers to discharge by an occupational therapist. To standardise usual care as much as possible, participants were expected to attend a minimum of 1 session and a maximum of 6 sessions of usual care physiotherapy.

Quality assurance checks took place at all CORKA research sites. They included fidelity checks during which assessment and treatment delivery were observed. All aspects of the intervention were checked against a pre-defined fidelity checklist created in line with the study protocol.

Outcomes

Baseline data were collected face-to-face no more than four weeks before surgery. Follow-up data collection was by face-to-face clinical assessments at 6 and 12 months following randomisation. Where face-to-face assessment was not possible, postal and telephone data collection methods were used to obtain self-reported core data. Baseline data included the Functional Co-morbidities index to provide baseline characteristics of the participants.

<u>Primary outcome</u>

The primary outcome was the Late Life Function and Disability Instrument (LLFDI) overall function score. It was developed specifically to assess change in community-dwelling older adults. It assesses and responds to meaningful change in two distinct outcomes: a person's ability to do discrete actions or activities using a 32-item *function* component (Primary outcome) and a person's performance of socially defined life tasks using a 16-item *disability* component (secondary outcome).

<u>Secondary outcomes</u>

Secondary outcomes consisted of self-reported and physical measures. The self-reported measures were the Oxford Knee Score (OKS), Physical Activity Scale for the Elderly (PASE), Knee

For peer review only

Injury and Osteoarthritis Outcome Score (KOOS) Quality of Life subscale, and EQ-5D-5L questionnaires. The physical measures were the Figure of 8 Walk Test (F8WT), 30 Second Chair Stand Test (30SCST), and Single Leg Stance (SLS). A health resource diary collected the exercises undertaken, medication taken, use of healthcare services and personnel, and falls.

Analysis

A detailed statistical analysis plan has previously been published (16). The LLFDI score was chosen as a clinically relevant outcome for the population and the sample size calculation was therefore based on this variable. As there is no formal minimally clinically important difference for the LLFDI the calculation was based on a moderately small standard effect size of 0.275 which gave a sample of 620 participants (310 per arm) with 90% power, 5% (two-sided) significance, allowing for 10% loss to follow up.

Two analysis populations were considered: the intention-to-treat (ITT) population and the per-protocol (PP) population. The ITT population included all randomised participants analysed according to their allocated intervention. The PP population included only participants who received at least one session of their allocated intervention, did not receive more treatment than intended (more than 6 sessions of usual care or 7 sessions of home-based rehabilitation), and provided follow-up data.

LLFDI function scores at 6 and 12-months post-randomisation were summarised by treatment group and analysed using a linear mixed effects model with repeated measures adjusted for baseline score and recruiting site (stratification factor). Time was treated as categorical and an interaction between the outcome measurement time point and randomised group was included to allow the treatment effect to be estimated at each time point, reported as the adjusted mean difference in LLFDI between groups with 95% CI and associated p-value. The underlying assumptions of this model were assessed. Twelve months post-randomisation was considered the primary endpoint. The primary analysis was performed for the ITT population using multiple imputation (MI) to impute missing data. MI was performed separately for each

For peer review only

treatment group and the model included type of knee replacement (TKR vs. UKR), recruiting centre, gender, whether or not the participant had had previous lower limb surgery, Charnley ABC score and current mobility support used. As sensitivity analyses this was repeated for the ITT population using available cases, the PP population using available cases and using a complier average causal effect (CACE) analysis (17).

Linear mixed effects models with repeated measures, similar to that described for the primary outcome, were used to analyse each of the secondary PROMs (LLFDI disability limitation, LLFDI disability frequency, OKS, KOOS QoL, PASE, EQ-5D- 5L utility and VAS), 30SCST (number of stands), and F8WT (time and steps). Differences between the two groups in SLS times were compared using a Wilcoxon rank-sum test since the assumptions of the linear mixed effects model were not satisfied. All of these analyses were performed for the ITT population using available cases only. For the key secondary outcomes (LLFDI disability limitation, LLFDI disability frequency, OKS, and KOOS QoL), these analyses were repeated for the ITT population using multiple imputation (imputation model as outlined for the primary outcome) and the PP population using available cases.

The number and proportion of SAEs were reported by treatment group, as well as the number and proportion of participants experiencing an SAE. The rates were compared across treatment groups.

Additional Analysis

An economic analysis and nested qualitative analysis were conducted as part of this study. Both economic and qualitative analysis will be reported elsewhere.

Patient and Public Involvement

The study was developed with a group of service users that identified concern about the access to rehabilitation for older more frail patients after knee arthroplasty. The intervention was developed with active involvement of PPI stakeholders who worked alongside us to develop the

For peer review only

intervention. A PPI representative collaborated on the application for funding and was a full voting member of the trial steering group which benefitted from their expertise and active engagement throughout the period of the study.

Results

The CORKA recruited participants between March 2015 and January 2018.2,788 patients were screened for eligibility. and 621 participants were randomised, 312 to usual care and 309 to the home based rehabilitation programme. A CONSORT flow chart summarises the flow of participants through the trial including details on the number of participants randomised and the numbers allocated to and receiving at least 1 session of each treatment (See Figure 1). Of the 621 participants, 34 (5.5%) withdrew from follow-up during the course of the trial. Three participants died between the 6 and 12 months follow ups.

The mean age of participants was 70.4 (SD 8.07), 59.7% were female and mean BMI was 31.5 (SD 4.74). Most participants scored 5 or 6 on the screening tool (79.5%) with 5 being the minimum score needed to be eligible for the study and indicating a risk of poor outcome. The baseline characteristics of the participants is summarised in Table 1.

Participants were defined as complying with usual care if they attended at least 1 treatment session and for home-based rehabilitation at least 4 treatment sessions. We used different thresholds for the two treatments as usual care varied considerably between sites including as little as one session. Therefore, compliance in this arm was defined as participants receiving at least one session. For the intervention arm, it was pre-specified that at least 4 of the 7 planned sessions were required to be compliant. Therapy compliance was 95.2% in usual care and 87.1% for the CORKA intervention. The median number of sessions for usual care was 4 (IQR 2,6) and for the CORKA intervention was 5 (IQR 4,7).

Primary outcome analysis

For peer review only - http://bmjopen.bmj.com/site/about/guidelines.xhtml

For the ITT analysis using multiple imputation there was no statistically significant difference between the two treatment groups at the primary time point of 12 months post-randomisation (adjusted difference 0.49, 95% CI: -0.89 to 1.88) or at 6 months (adjusted difference 0.66, 95% CI: -0.70, 2.03). This equates to a standardised effect size of 0.03 at 12 months.

Trends in LLFDI function scores over time are plotted for each treatment group in Figure 2. There was a substantial improvement between baseline and 6 months in both groups, with minimal additional difference between 6 and 12 months post-randomisation.

None of the sensitivity analyses conducted (ITT using available cases, per-protocol analysis, CACE analysis) demonstrated a different result.

Secondary outcome analysis

For the secondary PROMS ITT analysis using available cases, no significant differences between the two treatment groups at either time point (6 and 12 months) was identified for any of the secondary outcomes (see Table 2). The sensitivity analyses of the key secondary PROMS identified only one difference, of borderline statistical significance, for the LLFDI disability limitation at 6 months using the ITT population and multiple imputation (adjusted difference 2.67, 95% CI: 0.14 to 5.19,).

The physical measures are summarised by treatment group over time in Table 3. With the exception of one of the SLS tests, there were no differences in change in performance between the groups on the physical measures.

The total number of SAEs experienced in this trial was low; 18 events in 14 participants in the usual care group (4.5%) and 20 events in 18 participants in the CORKA intervention group (5.8%), experienced. Adverse events were independently reviewed and categorised using the System Organ Classes codes and as related to the intervention or not. For all SAEs only one was classified as ‘possibly related’. In total there were 7 SAEs in the musculoskeletal and connective

For peer review only

tissue disorder category, with one categorised as related to the treatment interventions (Table 4). The majority of the SAEs were related to medical conditions unconnected with the participant's surgery and were classified into categories such as cardiac disorders, renal and urinary disorder, or immune system disorders. There was not a significant difference in the number of participants experiencing an SAE between the two groups (Risk difference 1.3%, 95% CI -2.1% to 4.8%).

Discussion

No statistical or clinically meaningful differences were found between CORKA and usual out-patient physiotherapy. Outcomes across a range of measures were closely matched irrespective of which of the two interventions was received. The use of a workforce model using rehabilitation assistants offered flexibility of staffing and did not result in any increase in adverse events.

Strengths and limitations of the study

Strengths of this study include the large sample size and low loss to follow up which ensured sufficient numbers to make the study sufficiently powered to detect any between group differences. The trial was pragmatic and utilised an innovative workforce model that could readily translate into NHS practice within existing commissioning and financial constraints. The study offers an alternative service delivery model in line with the latest UK guidance from NICE. Limitations are that the screening tool did not identify patients that were a good fit of risk of poor outcome for the intervention that we developed. The screening tool has not been validated; but uses items that are used by many knee arthroplasty trials to determine entry, these are broad and inclusive, reflecting common clinical practice. Recent recommendations from the UK National Institute for Health and Clinical Excellence noted that it would be useful if a tool existed that indicated those who would benefit from supervised rehabilitation and recognised that there is a significant proportion of patients to whom this will apply (18). In developing our screening tool for the trial, we attempted just this, but recognise that there was

For peer review only

a poor match between the patients screened and the intervention that we developed. Many of our participants were borderline high risk scoring just above the cut-point of 5, resulting in fewer participants at higher risk than anticipated which may have affected the results. Another limitation, common to all trials of post-operative physiotherapy after knee arthroplasty, was the absence of a control group. As out-patient physiotherapy remains an accepted and expected core component of UK arthroplasty practice it was not deemed ethical or practicable to attempt to recruit to a no treatment comparator in a group who had already been identified as at higher risk of a poor outcome. As with most physiotherapy trials it was not feasible to blind patients or therapists to treatment allocation. We also chose to deliver an intervention that met current commissioning guidance of around six sessions. It is arguable that more intensive treatment as is common in other countries may have been more effective, but this is not supported by studies using longer interventions (19-24).

We chose to use the LLFDI as our primary outcome over a more knee focused outcome as it is designed to map to the WHO ICF and assess change in community dwelling older adults encompassing the range of activities relevant for older, potentially frailer patients after arthroplasty. At the time of designing the trial the MCID was not published. Subsequently, Beauchamp et al reported that substantial changes are reflected in a 5-point change in the overall function scale (25). We found substantial changes from baseline to 1 year within both groups, but no between group effect. The CORKA home-based intervention showed an overall change of 8.9 points and usual care 8.7 points, indicating that the combined effect of surgery and rehabilitation unsurprisingly produced a highly significant improvement in function. Most of this improvement was probably due to the surgical procedure, but we cannot disaggregate the contribution of physiotherapy to quantify the contribution of one without the other. We found no significant between group difference. It is arguable that a more specific outcome measure targeted at knee arthroplasty may have been a better choice. We included the OKS as a secondary measure to address this potential limitation. The results of the LLFDI and OKS were well-matched.

Implications for patients and policy

For peer review only

The benefit of post-operative physiotherapeutic interventions are poorly established. A recent evidence review by NICE, of all patients receiving arthroplasty surgery, found no clinically important differences in quality of life, PROMS or functional outcomes between group or individually based supervision and self-directed rehabilitation. They recommended self-directed rehabilitation as an acceptable option for people who are well enough and have personal circumstances that make it possible; but recognised that there are likely to be subgroups who will benefit from a more supervised rehabilitation programme. The CORKA trial is one of few trials to specifically target individuals who are at risk of poor outcome post surgery. The TRIO trial (26) also targeted physiotherapy at patients considered at risk of poor outcome, based upon Oxford Knee Score at 6 weeks. Similar to this study they found no statistical differences between the two treatment arms of outpatient physiotherapy and a single physiotherapy review and home exercise based regimen.

Although we screened for patients deemed at risk of poor outcome the OKS of 37.8 points was higher than the reported average for all knee arthroplasty patients at one year of 36 points.. The TRIO trial similarly found their results, although a little lower, were similar to the unselected average (26). Most of this improvement was probably due to the surgical procedure, but we cannot disaggregate the contribution of physiotherapy to quantify the contribution of one without the other.

The CORKA intervention was based on pre-operative outcome measures, with a home-based rehabilitation programme. There may also be benefits to home supervised rehabilitation that are not captured by the current evidence or the outcome measures used in existing studies. For example, participants in our embedded qualitative study were very positive about the CORKA home-based intervention and the supervision provided by the therapists and rehabilitation assistants (27). The CORKA home-based intervention could also benefit, for example, those too frail to travel a significant distance to a larger hospital. Using the CORKA combination of qualified therapists and rehabilitation assistants might be a practical option for certain remote areas of the UK or countries that have large geographical areas not covered by therapists. This

For peer review only

suggests there is an argument for choice, according to personal preference or the practicalities of service provision.

It also addresses the workforce shortage issue by using an innovative workforce model of advanced rehabilitation assistants, moving UK service provision closer to that which has been proven to be effective in North America, where the use of physical therapy assistant graded staff is well embedded. This is a particular concern given both the projected increased need for joint arthroplasty over the next decade to accommodate an ageing population and the pressure of potential reductions in NHS funding. Evaluating the value of treatment modalities offered to these patients is crucial because many more patients are being discharged home earlier from the acute setting leaving less time available for acute physical recovery, rehabilitation, and education in hospital, increasing the potential burden of care for these patients and their families.

Future research

Further research should focus on developing a screening tool that is more sensitive in identifying those patients who will benefit from additional input beyond self-directed rehabilitation as recommended by the NICE guidance on primary joint replacement (18).

The CORKA home-based intervention was delivered by rehabilitation assistants supervised by qualified therapists in a ratio of 5:2 sessions. Given the workforce challenges in the NHS further work looking at different workforce models and interventions solely using rehabilitation assistants should be explored.

Conclusions

This large randomised controlled trial found no important differences in outcomes when rehabilitation was delivered using either a home-based, rehabilitation assistant delivered

For peer review only

rehabilitation package or a traditional out-patient physiotherapy model. Home based rehabilitation was feasible but did not confer benefits over standard out patient physiotherapy.

Contributorship Statement:

Karen L Barker (Professor of Physiotherapy) – Chief Investigator, led funding application, trial conception and design, development of interventions, supervision, and writing and reviewing report.

Jonathan Room (Research Physiotherapist) – Principal Investigator, development of intervention, and writing and reviewing report.

Ruth Knight (Statistician) – Conducting statistical analysis and writing and reviewing report.
Seamus Kent (Health Economist) – Conducting economic analysis and writing and reviewing report.

Susan Dutton (Statistician) – Designing and conducting statistical analysis and writing and reviewing report.

Fran Toye (Qualitative lead) -, conducting interviews and analysis, and writing chapter for report.

Jose Leal (Health Economist) – Designing and conducting economic analysis, supervision, and writing and reviewing report.

Nicola Kenealy (Trial Manager) – Trial management and writing and reviewing report.

For peer review only

Michael Maia Schlussel (Statistician) – Screening tool development and writing for report.

Gary Collins (co-applicant) – Supervised screening tool development.

David Beard (co-applicant) – Clinical trials expertise and contributed to report.

Andrew Price (co-applicant) – Surgical expertise and contributed to report.

Martin Underwood (co-applicant) – Primary care expertise and contributed to report.

Avril Drummond (co-applicant) – Occupational therapy expertise and contributed to report.

Sarah Lamb (co-applicant) – Clinical trials expertise, key member of trial management group, and writing and reviewing report.

Acknowledgements:

The authors would like to acknowledge the site principal investigators and research clinicians

Leon Palmer-Wilson, Ana Glennon (Ashford Hospital), Helen Wilson (Countess of Chester Hospital), Christian Brookes, Denise Hill (Dorset County Hospital), Susan Dowdle, Hazel Burt (Dorset HealthCare Hospital), Jane Harrison (Epsom Hospital), Sarah Adcock, Justine Theaker (Manchester Royal Infirmary), Gladys Nadar Arulmani (Medway Community Hospital), Sunil Jain (Medway Maritime Hospital), Mike Reed (North Tyneside General Hospital), Jonathan Room (Oxford University Hospitals), Genevieve Simpson, Gemma Knight (Royal United Hospitals Bath), Tricia Monroe (Moorgreen Hospital), Gareth Stephens, Sarah Rich (Royal Orthopaedic Hospital Birmingham).

Funding support: This trial was funded by the National Institute for Health Research Health Technology Assessment programme (HTA 12/196/08)

For peer review only

Conflict of Interest:

G Collins is a member of the HTA Commissioning Board

A Price has a consultancy with Zimmer Biomet, outside the submitted work

M Underwood has grants from NIHR, during the conduct of the study; grants from NIHR, Personal fees from NIHR, Personal fees from NICE, non financial support from Stryker PLC, grants from SERCO ltd.

S Lamb has grants from NIHR Health Technology Assessment Programme during the conduct of the study and is a member of the following Boards:

HTA Additional Capacity Funding Board 2010-2015

HTA Clinical Trials Board 2010-2015

HTA End of Life Care and Add on Studies 2015-2015

HTA Funding Boards Policy Group (Formally CSG) 2010-2015

HTA MNCH Methods Group 2013-2015

HTA Post-board funding teleconference (PG members to attend) 2010-2015

HTA Primary Care Themed Call board 2013-2014

HTA Prioritisation Group 2014-2015

NIHR CTU Standing Advisory Committee 2012-2016

Ethical approvals

The study protocol was approved by the South-Central Research Ethics Committee (Reference 15/SC/0019). Ethics permission was obtained for all participating sites.

Data Sharing

Deidentified individual participant data will be available on reasonable request to the Chief Investigator. Data will be available beginning 9 months and ending 36 months following article publication to investigators who provide a methodologically sound proposal. Proposals may be

For peer review only

submitted up to 36 months following article publication. To gain access data requestors will need to sign a data access agreement and the study in question.

Figure Caption

Figure 1 - CONSORT flow chart

Figure 2 - The LLFDI function score from baseline to 12 months post randomisation for each treatment group

References

1. National Joint Registry for England, Wales NI and the I of M. 15th Annual Report 2018. www.hqip.org.uk/wp-content/uploads/2018/11/NJR-15th-Annual-Report-2018. Accessed july 2021.
2. Jones CA, Beaupre LA, Johnston DWC, Suarez-Almazor ME. Total Joint Arthroplasties: Current Concepts of Patient Outcomes after Surgery. Rheumatic Disease Clinics of North America. 2007;33(1):71-86..
3. Naylor J, Harmer A, Fransen M, Crosbie J, Innes L. Status of physiotherapy rehabilitation after total knee replacement in Australia. Physiother Res Int. 2006;11(1):35-47.
4. Westby MD, Kennedy D, Jones DL, Jones A, Doyle-Waters M, Backman C. Post-acute physiotherapy for primary total knee arthroplasty. Cochrane Database of Systematic Reviews. 2008 (2).
5. Moffet H, Collet JP, Shapiro SH, Paradis G, Marquis F, Roy L. Effectiveness of intensive rehabilitation on functional ability and quality of life after first total knee arthroplasty: A single-blind randomized controlled trial. Arch Phys Med Rehabil. 2004;85:546-56.
6. Petterson SC, Mizner RL, Stevens JE, Raisis LEO, Bodenstab A, Newcomb W, et al. Improved function from progressive strengthening interventions after total knee arthroplasty: A randomized clinical trial with an imbedded prospective cohort. Arthritis Care Res. 2009;61 (2):174-83.
7. Rajan RA, Pack Y, Jackson H, Gillies C, Asirvatham R. No need for outpatient physiotherapy following total knee arthroplasty: A randomized trial of 120 patients. Acta Orthop Scand. 2004;75 91):71-3.;
8. Piva SR, Gil AB, Almeida GJM, DiGioia AM, Levison TJ, Fitzgerald GK.. A balance exercise program appears to improve function for patients with total knee arthroplasty : a randomized clinical trial.Phys Ther 2010;90: 880-94.
9. Artz N, Dixon S, Wylde V, Marques E, Beswick AD, Lenguerrand E, et al. Comparison of group-based outpatient physiotherapy with usual care after total knee replacement: A feasibility study for a randomized controlled trial. Clin Rehabil. 2017;31 (4):487-99.
10. Fillingham YA, Darrith B, Lonner JH, Culvern C, Crizer M, Della Valle CJ. Formal Physical Therapy May Not Be Necessary After Unicompartmental Knee Arthroplasty: A Randomized Clinical Trial. J Arthroplasty. 2018;33 (7s): S93.
11 Judge A, Arden NK, Cooper C, Kassim Javaid M, Carr AJ, Field RE, et al. Predictors of

For peer review only

outcomes of total knee replacement surgery. Rheumatology (Oxford). 2012;51(10):1804-13
12. Shubert TE, Smith ML, Goto L, Jiang L, Ory MG. Otago Exercise Program in the United States: Comparison of 2 Implementation Models. Phys Ther. 2017;97(2):187-97.
13. Barker KL, Beard D, Price A, Toye F, Underwood M, Drummond A, Collins G, Dutton S, Campbell H, Kenealey N, Room J, Lamb SE. Community-based rehabilitation after knee arthroplasty (CORKA): study protocol for a randomised controlled trial. Trials 2016 Oct 13;17(1):501.
14. Schlussel M, Collins G, Dutton S, Barker K. Development of the CORKA trial screening tool for identifying patients at increased risk of poor outcome following knee replacement. Trials. 2017;18:S1, P430.
15. Room J, Batting M, Barker KL. Development of a functional rehabilitation intervention for post knee arthroplasty patients: community based Rehabilitation post Knee Arthroplasty (CORKA) trial. Physiotherapy. 2020 Mar;106:52-64.
16. Vadher K, Knight R, Barker KL, Dutton SJ. Community based rehabilitation after knee arthroplasty (CORKA): statistical analysis plan for a randomised controlled trial. Trials. 2018 Nov 19;19(1):638
17. Dunn G, Maracy M, Tomenson B. Estimating treatment effects from randomized clinical trials with noncompliance and loss to follow-up: the role of instrumental variable methods. Stat Methods Med Res. 2005;14(4):369-95.
18. National Institute for Health and Care Excellence. Joint replacement (primary): hip, knee and shoulder [R] Evidence review for outpatient hip and knee postoperative rehabilitation. NICE guideline [NG157]. 2020. www.nice.org.uk/guidance/ng157. Accessed 16.06.2020.
19. Hamilton DF, Loth FC, MacDonald DJ, MacFarlane GJ, Beard DJ, Simpson AHRW, et al. Exploring variation in patient access of post-discharge physiotherapy following total hip and knee arthroplasty under a choice based system in the UK: An observational cohort study. BMJ Open. 2019;
20. Khan F, Ng L, Gonzalez S, Hale T, Turner-Stokes L. Multidisciplinary rehabilitation programmes following joint replacement at the hip and knee in chronic arthropathy. Cochrane Database of Systematic Reviews. 2008.
21. Artz N, Elvers KT, Lowe CM, Sackley C, Jepson P, Beswick AD. Effectiveness of physiotherapy exercise following total knee replacement: Systematic review and meta-analysis. BMC Musculoskeletal Disorders. 2015.
22. Roos EM. Effectiveness and practice variation of rehabilitation after joint replacement. Current Opinion in Rheumatology. 2003.
23. Smith TO, Dainty JR, Clark EM, Whitehouse MR, Price AJ, MacGregor AJ. Demographic and geographical variability in physiotherapy provision following hip and knee replacement. An analysis from the National Joint Registry for England, Wales, Northern Ireland and the Isle of Man. Physiotherapy 2020;106:1-11. doi:10.1016/j. physio.2019.11.003
24. Buhagiar MA, Naylor JM, Harris IA, Xuan W, Adie S, Lewin A. Assessment of Outcomes of Inpatient or Clinic-Based vs Home-Based Rehabilitation After Total Knee Arthroplasty: A Systematic Review and Meta-analysis. JAMA Netw Open 2019;2:e192810. doi:10.1001/

For peer review only

jamanetworkopen.2019.2810

25 Beauchamp MK, Ward RE, Jette AM, Bean JF. Meaningful Change Estimates for the Late-Life Function and Disability Instrument in Older Adults. J Gerontol A Biol Sci Med Sci. 2019;74(4):556-9.

26. Hamilton DF, Beard DJ, Barker KL, Macfarlane GJ, Tuck CE, Stoddart A, Wilton T, Hutchinson JD, Murray GD, Simpson AHRW. Targeting rehabilitation to improve outcomes after total knee arthroplasty in patients at risk of poor outcomes: randomised controlled trial. BMJ 2020 Oct 13;371:m3576.

27. Barker KL, Room J, Knight R, Dutton SJ, Toye F, Leal J, Kent S, Kenealy N, Schussel MM, Collins G, Beard DJ, Price A, Underwood M, Drummond A, Cook E, Lamb SE. COmmunity-based Rehabilitation after Knee Arthroplasty: A randomised controlled trial with economic and qualitative evaluations (CORKA Trial). Health Technology Assessment: 2020; 24: 1-116

For peer review only

Table 1 - Descriptive characteristics at baseline by treatment group

	Usual care (n = 312)	Home-based rehab (n = 309)	Total (n = 621)
**Gender[a]**			
Male	125 (40.1%)	125 (40.5%)	250 (40.3%)
Female	187 (59.9%)	184 (59.5%)	371 (59.7%)
**Age (years)[b]**	70.18 (8.14)	70.67 (8.01)	70.42 (8.07)
**BMI[b]**	31.65 (4.99)	31.34 (4.48)	31.50 (4.74)
**Side of operation[a]**			
Right	169 (54.2%)	169 (54.7%)	338 (54.4%)
Left	142 (45.5%)	139 (45.0%)	281 (45.2%)
Not recorded	1 (0.3%)	1 (0.3%)	2 (0.3%)
**Knee arthroplasty type[a]**			
TKA	229 (73.4%)	231 (74.8%)	460 (74.1%)
UKA	82 (26.3%)	77 (24.9%)	159 (25.6%)
Not recorded	1 (0.3%)	1 (0.3%)	2 (0.3%)
**ASA grade[a]**			
Healthy	38 (12.2%)	55 (17.8%)	93 (15.0%)
Mild systemic disease	218 (69.9%)	202 (65.4%)	420 (67.6%)
Severe systemic disease	43 (13.8%)	44 (14.2%)	87 (14.0%)
Not recorded	13 (4.2%)	8 (2.6%)	21 (3.4%)
**Falls in the last year[a]**			
Yes	77 (24.7%)	89 (28.8%)	166 (26.7%)
No	235 (75.3%)	220 (71.2%)	455 (73.3%)
**If yes, number of falls[c]**	1 (1, 3)	2 (1, 3)	2 (1, 3)
**Previous lower limb surgery[a]**			
Yes	200 (64.1%)	189 (61.2%)	389 (62.6%)
No	112 (35.9%)	120 (38.8%)	232 (37.4%)
**Screening tool score[c]**	6 (5, 6)	6 (5, 6)	6 (5, 6)

For peer review only

**Charnley ABC**[a]			
A - single knee arthroplasty	134 (42.9%)	138 (44.7%)	272 (43.8%)
B - both knees affected	140 (44.9%)	145 (46.9%)	285 (45.9%)
C - multiple joint disease/other disability	38 (12.2%)	26 (8.4%)	64 (10.3%)
**Stairs mobility**[a]			
Normal	19 (6.1%)	19 (6.1%)	38 (6.1%)
One step at a time	34 (10.9%)	39 (12.6%)	73 (11.8%)
Down with rail	18 (5.8%)	19 (6.1%)	37 (6.0%)
Up/down with rail	225 (72.1%)	216 (69.9%)	441 (71.0%)
Unable down	2 (0.6%)	3 (1.0%)	5 (0.8%)
Unable	14 (4.5%)	12 (3.9%)	26 (4.2%)
Missing	0 (0.0%)	1 (0.3%)	1 (0.2%)
**Support mobility**[a]			
None	178 (57.1%)	178 (57.6%)	356 (57.3%)
Stick outdoors	83 (26.6%)	78 (25.2%)	161 (25.9%)
Stick always	34 (10.9%)	31 (10.0%)	65 (10.5%)
2 sticks	6 (1.9%)	7 (2.3%)	13 (2.1%)
2 crutches	5 (1.6%)	7 (2.3%)	12 (1.9%)
Walking frame	6 (1.9%)	8 (2.6%)	14 (2.3%)
**Functional comorbidity index**[d]			
0	189 (60.6%)	176 (57.0%)	365 (58.8%)
1-3	112 (35.9%)	125 (40.5%)	237 (38.2%)
4-6	8 (2.6%)	6 (1.9%)	14 (2.3%)
7+	2 (0.6%)	0 (0.0%)	2 (0.3%)
Missing	1 (0.3%)	2 (0.6%)	3 (0.5%)

ASA = American Society of Anaesthesiologists; BMI = body mass index; TKA = total knee arthroplasty; UKA = unicompartmental knee arthroplasty.
[a] These values are reported as numbers and percentages
[b] These values are reported as means and standard deviations
[c] These values are reported as medians and interquartile ranges
[d] The functional comorbidity index (85) counts the number of comorbidities experienced by each patient, giving more weight to more severe conditions. This was categorised in four groups: (i) no comorbidities (0), (ii) small number of comorbidities (1-3), (iii) medium number of comorbidities (4-6), and (iv) high number of comorbidities (7+).

For peer review only

Table 2 - Comparison of treatment groups for secondary outcomes (intention-to-treat population using available cases)

		Usual care		Home-based rehab		Adjusted difference (95% CI)[1]	p-value
		Mean (SD)	Total	Mean (SD)	Total		
**LLFDI Disability (frequency)**	**Baseline**	51.28 (7.25)	311	51.55 (7.46)	308	-	-
	**6 months**	54.27 (6.86)	283	54.91 (6.85)	275	0.64 (-0.50, 1.78)	0.27
	**12 months**	54.13 (6.87)	287	55.05 (6.87)	279	0.93 (-0.21, 2.06)	0.11
**LLFDI Disability (limitation)**	**Baseline**	66.26 (11.96)	312	66.25 (12.48)	305	-	-
	**6 months**	72.33 (15.25)	284	74.72 (15.24)	275	2.39 (-0.14, 4.92)	0.06
	**12 months**	74.10 (15.27)	287	74.75 (15.28)	277	0.65 (-1.87, 3.18)	0.61
**OKS**	**Baseline**	20.59 (7.50)	312	20.81 (7.31)	308	-	-
	**6 months**	35.69 (7.69)	284	36.23 (7.69)	277	0.54 (-0.73, 1.81)	0.40
	**12 months**	37.34 (7.71)	287	37.80 (7.71)	278	0.46 (-0.81, 1.73)	0.48
**KOOS – QoL subscale**	**Baseline**	25.50 (16.58)	301	25.50 (15.89)	300	-	-
	**6 months**	61.26 (22.31)	273	62.47 (22.29)	269	1.21 (-2.55, 4.97)	0.53
	**12 months**	65.53 (22.37)	277	65.26 (22.36)	271	-0.27 (-4.02, 3.47)	0.89
**PASE**	**Baseline**	121.24 (73.90)	302	115.11 (72.95)	297	-	-
	**6 months**	150.62 (66.72)	259	149.41 (66.46)	245	-1.22 (-12.86, 10.42)	0.84
	**12 months**	156.58 (66.82)	263	154.63 (66.73)	251	-1.95 (-13.51, 9.61)	0.74
**EQ5D-5L Utility**	**Baseline**	0.52 (0.22)	308	0.52 (0.23)	308	-	-
	**6 months**	0.75 (0.18)	277	0.75 (0.18)	275	0.00 (-0.03, 0.03)	0.85
	**12 months**	0.76 (0.18)	282	0.76 (0.18)	278	-0.00 (-0.03, 0.03)	0.99
**EQ5D-5LVAS**	**Baseline**	68.53 (19.46)	312	68.03 (19.13)	308	-	-
	**6 months**	75.81 (14.55)	283	75.51 (14.54)	275	-0.30 (-2.71, 2.11)	0.81
	**12 months**	76.80 (14.57)	287	77.05 (14.58)	278	0.24 (-2.16, 2.65)	0.84

For peer review only

Notes: Totals refer to the total number of participants in each group that provided each score at each timepoint. Adjusted differences were calculated using linear mixed effects models adjusted for stratification factors and baseline scores. LLFDI = Late Life Function and Disability Instrument, each LLFDI disability scale ranges from 0 to 100 with higher scores indicating less disability. OKS = Oxford Knee Score, scores range from 0 to 48 with higher scores indicating better knee function. KOOS = Knee injury and Osteoarthritis Outcome Score, scores range from 0 to 100 with higher scores indicating better QoL. PASE = Physical Activity Scale for the Elderly, higher scores indicate greater levels of physical activity. EQ5D-5L utility scores range from -0.594 to 1 and EQ5D-5L visual analogue scale (VAS) ranges from 0 to 100, in both cases higher scores indicate better quality of life. CI = confidence interval; SD = standard deviation.

For peer review only

Table 3 - Comparison of physical measures between treatment groups at follow-up time points

	Usual care (n = 312)		Home-based rehab (n = 309)		Adjusted difference (95% CI)	p-value
	Summary	N	Summary	N		
30SCST # stands[a]						
**Baseline**	8.2 (3.5)	312	8.4 (3.7)	309	-	-
**6 months**	11.1 (3.1)	278	11.0 (3.1)	275	-0.06 (-0.548, 0.46)	0.82
**12 months**	11.7 (3.1)	279	11.5 (3.1)	276	-0.22 (-0.74, 0.3)	0.41
F8WT (seconds)[a]						
**Baseline**	11.8 (5.4)	312	12.1 (4.9)	309	-	-
**6 months**	10.2 (3.6)	277	9.6 (3.6)	275	-0.57 (-1.17, 0.04)	0.07
**12 months**	9.3 (3.6)	280	9.1 (3.6)	274	-0.17 (-0.78, 0.43)	0.58
F8WT steps[a]						
**Baseline**	16.4 (4.5)	312	17.3 (5.0)	309	-	-
**6 months**	15.5 (3.2)	277	15.2 (3.2)	275	-0.36 (-0.91, 0.18)	0.19
**12 months**	15.2 (3.2)	280	14.9 (3.3)	274	-0.23 (-0.77, 0.31)	0.40
KA leg SLS average (seconds)[a]						
**Baseline**	5.3 (2.6, 13.0)	310	4.5 (1.9, 14.5)	308	-	-
**6 months**	10.0 (3.9, 30.3)	279	10.0 (3.6, 26.8)	276	-	0.57
**12 months**	13.3 (5.9, 31.1)	280	11.7 (3.4, 31.1)	276	-	0.14
Contralateral leg SLS average[b] (seconds)						
**Baseline**	7.2 (3.6, 22.0)	310	6.3 (2.6, 18.0)	308	-	-
**6 months**	10.9 (4.2, 30.8)	279	9.8 (3.5, 29.7)	276	-	0.43
**12 months**	14.7 (5.6, 30.7)	280	11.0 (3.2, 29.7)	276	-	0.03

Notes: Totals refer to the total number of participants in each group that provided each score at each timepoint. 30SCST = 30 Seconds Chair Stand Test, which records the number of stands in 30 seconds and where higher scores indicate better function. F8WT = Figure of 8 Walk Test, where a quicker time and fewer steps indicates better function. SLS = Single Leg Stance, in which stance time is averaged over three trials at standing on the same leg, with a maximum score of 45 seconds and higher scores indicate better function

[a] Summaries are mean (SD)

[b] Summaries are median (IQR)

For peer review only

Table 4: SOC codes of SAEs by treatment group

	Usual Care (n= 14; 4.5%)	Home-based Rehab (n= 18; 5.8%)
**Blood/lymphatic**	2	2
**Cardiac**	2	1
**Endocrine**	1	0
**Gastrointestinal**	2	2
**Immune system**	0	1
**Infections/infestations**	1	3
**Musculoskeletal**	1	6
**Nervous system**	0	1
**Renal/urinary**	2	0
**Respiratory/thoracic**	0	1
**Skin**	1	0
**Social circumstances**	1	0
**Vascular**	1	0
**Unknown**	0	1

For peer review only

Figure 1 - CONSORT flow chart

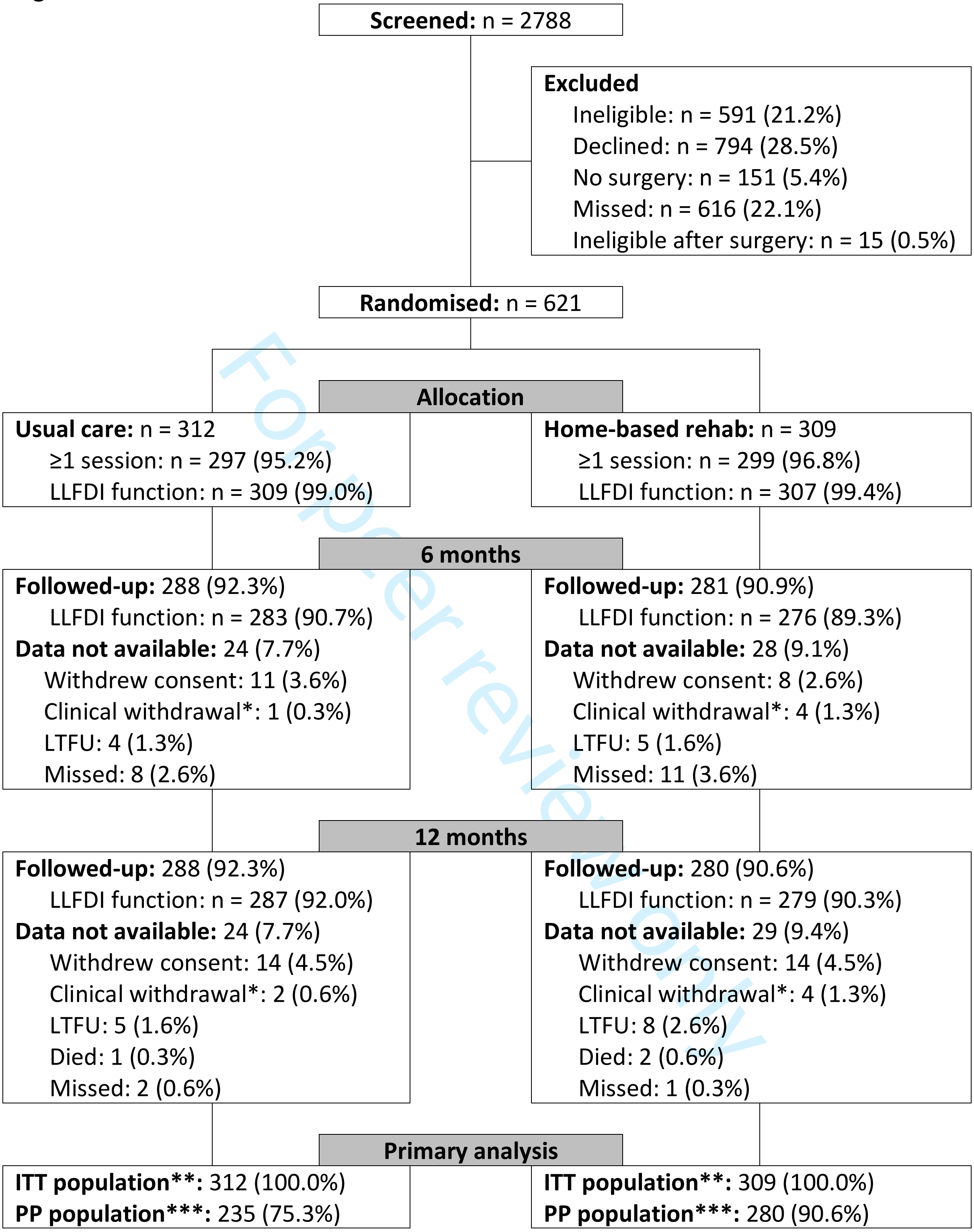

**Clinical withdrawals include those who withdrew due to an adverse event, serious adverse event, or medical contraindication*

**Missing follow-up data in the intent-to-treat (ITT) population was imputed using multiple imputation for the primary outcome analysis.*

***Analysis of the per-protocol (PP) population was based on available data only.*

LLFDI: Late Life Function and Disability Instrument; LTFU: lost to follow-up

For peer review only

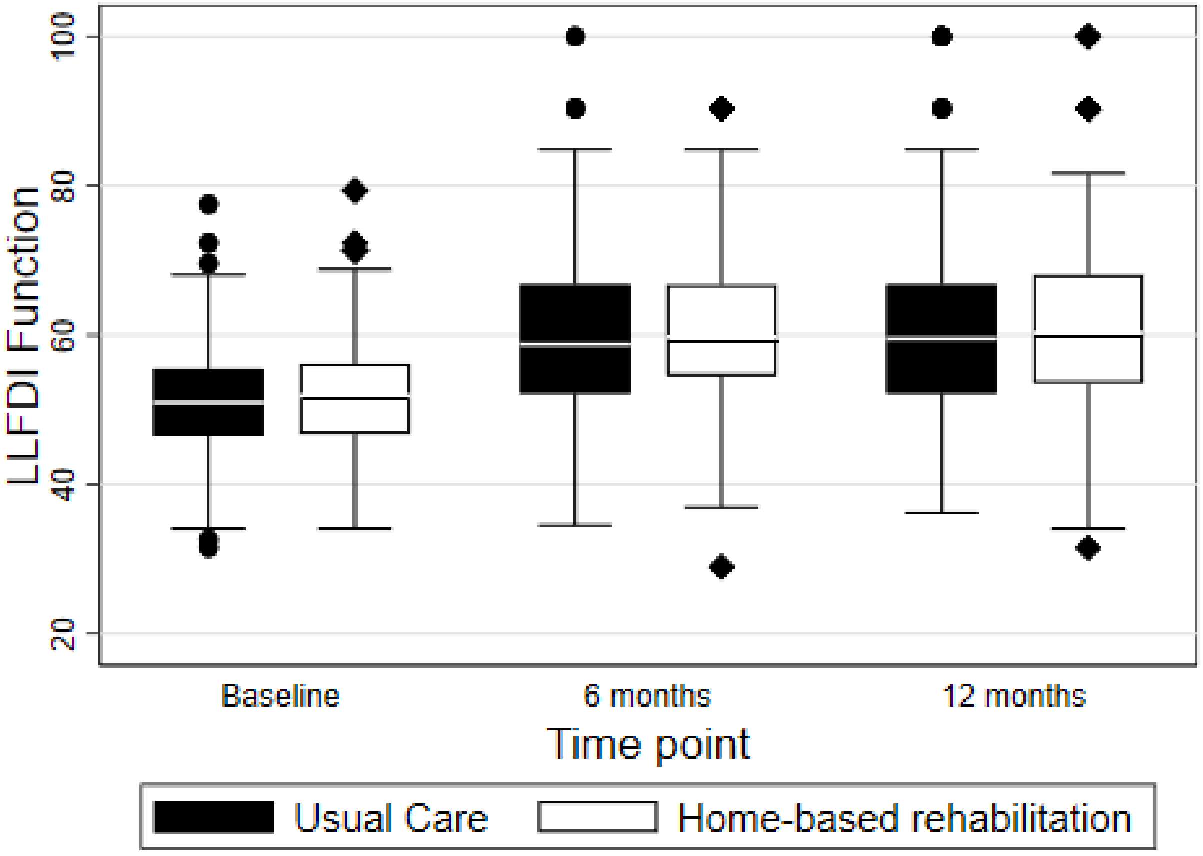
LLFDI Function
100
80
60
40
20
Baseline
6 months
12 months
Time point
Usual Care
Home-based rehabilitation

Corka

CORKA Screening Tool

Study code:	Site ID code:	Participants Study Number*:	Initials:
C K	–	–	

* Add in once Participant has been randomised

DOB: _ _/_ _ _/_ _ _ _ **Principal Investigator:** ________________ Date _ _/_ _ _/_ _ _ _

Participant Screening Number (number given when participant originally screened) CSN __ __ __ __

From pre-admission clinic, complete the following:

SCORE *(Please Circle)*

1. What is the participant's Body Mass Index (BMI)?

Normal weight (<25kg/m^2)	**0**
Overweight (25-29.9kg/m^2)	**1**
Obese (≥30kg/m^2)	**2**

2. From Q1 of the Oxford Knee Score (OKS) questionnaire;

'How would you describe the pain you <u>usually</u> have from your knee?'

None OR very mild OR mild	**0**
Moderate OR severe	**4**

3. What is the participant's health status?

Fit and healthy with mild systemic disease i.e. controlled hypertension/diabetes	**0**
Severe systemic disease i.e. unstable coronary heart disease	**2**

4. In the **past four weeks** have you been limited in the kind of work you do or other regular activities you carry out as a result of feeling anxious or depressed?

None of the time OR a little of the time OR some of the time	**0**
Most of the time OR all of the time	**2**

TOTAL: ☐

For patients with a score of 5 or more, continue to screen for eligibility onto the study.

For patients with a score below 5, stop the screening process and record this in the screening log.

For peer review only

For peer review only - http://bmjopen.bmj.com/site/about/guidelines.xhtml

Appendix B – content of treatment Interventions

The CORKA home-based intervention was a multicomponent rehabilitation programme delivered to participants in their own home by a mixture of qualified staff and rehabilitation assistants. Its main component was an exercise programme, but it also included functional task practice, adherence approaches and provision of appropriate aids or equipment. It consisted of an initial appointment and 6 follow up sessions.

Initial appointment – assessment

The subjective assessment included their current symptoms and the perceived level of pain or discomfort on a VAS scale from 0-10 with 0 being 'no symptoms' and 10 the 'worse imaginable symptoms', their social history, any medication taken, their history of falls and their activities of daily living (ADLs).

The objective assessment documented the participant's wound, the range of movement in the operated and non-operated knees, strength of quadriceps, hamstrings, hip abductors and the calf muscles, scored on an Oxford grading scale. Standing balance was assessed by observing and recording the ability to stand with feet side by side, or in a semi-tandem or tandem stance. An assessment was made of gait and if appropriate ability to ascend and descend stairs. If deemed necessary and safe any transfers were assessed.

Finally, observation of at least one functional task took place which was identified by the participant as being potentially problematic. The exercise programme was subsequently tailored to meet the specific needs, goals and functional problems of the individual.

Initial appointment – exercise programme

Following the assessment the physiotherapist and rehabilitation assistant set each participant an exercise programme, which would replace any post-operative programme given on discharge from hospital. It would consist of exercises categorised into the following sections :

1. Knee range of motion - flexion
2. Knee range of motion – extension
3. Basic quadriceps strengthening
4. Strengthening exercises – quadriceps
5. Strengthening exercises – hamstrings
6. Strengthening exercises – hip abductors
7. Strengthening exercises – calf
8. Static balance exercises
9. Dynamic balance and walking skills exercises

The selection of each specific exercise took place as follows, for ROM exercises the physiotherapist selected an exercise and then checked that they and the participant were happy with how it was performed. If this was the case the participant was asked to perform 3 sets of 10-15 repetitions, unless a stretch had been selected, when they were asked to undertake 3 sets of 3-5 repetitions with a 30 to 60 second hold.

For strengthening exercises the participant was given an exercise that they could perform for 6-12 repetitions with good form at a moderate intensity. Perceived intensity was measured using the 10 point Rating of Perceived Exertion (RPE) scale (Borg 1998). The participant was given an exercise

For peer review only

where they feel they were working at RPE of 3-4 (moderate to somewhat hard). Participants were asked to complete 1 to 3 sets of 6-12 reps of strengthening exercises.

For the balance section, participants were prescribed an exercise that was sufficiently challenging without putting the participant at risk of falling. Participants were asked to perform 3 repetitions of the exercise with a 30 second hold.

For the final section, gait skills there were a variety of exercises to choose from. The physiotherapist was asked to prescribe an exercise that was sufficiently challenging without putting the participants at risk of falling. The exact number of sets and reps were determined based on the particular exercise selected and the individual patient.

During session 3 a graduated walking programme was introduced. Participants were asked about the maximum time or distance that they have managed to walk since surgery and were encouraged to increase this distance or time gradually in small increments.

Participants were also asked to practice between 1-3 specific functional tasks highlighted in the initial assessment.

<u>CORKA intervention exercise prescription time points</u>

	Range of Movement	Basic Quads strength	Strengthening	Balance	Gait Skills	Task practice	Walking programme
Session 1	✓	✓	✓	✓	✓	✓	
Session 2	✘	✘	⇧	⇧	⇧	⇧	
Session 3	✘	✘	⇧	⇧	⇧	⇧	✓
Session 4	✘	✘	⇧	⇧	⇧	⇧	⇧
Session 5	✘	✘	⇧	⇧	⇧	⇧	⇧
Session 6	✘	✘	⇧	⇧	⇧	⇧	⇧
Session 7	✘	✘	⇧	⇧	⇧	⇧	⇧

✓ = issue exercise

✘ = Stop exercise once appropriate level of function reached

⇧ = Progress exercise

For peer review only

CORKA Intervention Exercises Table

Exercise	Level	Purpose	Functional relevance
Hip and knee flexion 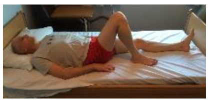	1A	Lower limb range of movement	Walking, stair climbing, kneeling
Passive knee flexion 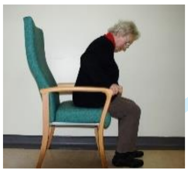	1B	Lower limb range of movement	Walking, stair climbing, sitting and standing up from a chair, kneeling
Active assisted knee flexion 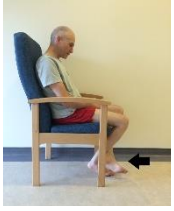	1C	Lower limb range of movement	Walking, stair climbing, sitting and standing up from a chair, kneeling
Hold-relax knee flexion 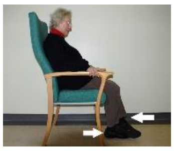	1D	Lower limb range of movement	Walking, stair climbing, sitting and standing up from a chair, kneeling
Active Knee flexion 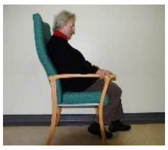	1E	Lower limb range of movement	Walking, stair climbing, sitting and standing up from a chair, kneeling
Hip and knee flexion with overpressure 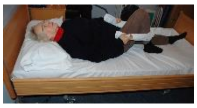	1F	Lower limb range of movement	Walking, stair climbing, kneeling
Knee extension stretch 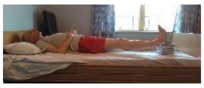	2A	Lower limb range of movement	Walking, stair climbing, standing up from a chair

For peer review only

Knee extension with overpressure 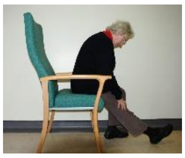	2B	Lower limb range of movement	Walking, stair climbing, standing up from a chair
Knee extension 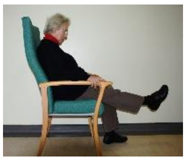	2C	Lower limb range of movement	Walking, stair climbing, standing up from a chair, dressing, getting in/out of car
Hamstring stretch 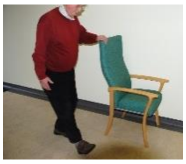	2D	Lower limb range of movement	Walking, stair climbing, dressing
Calf stretch 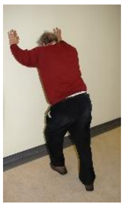	2E	Lower limb range of movement	Walking, stair climbing, dressing
Static Quadriceps 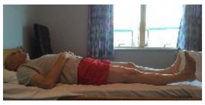	3A	Lower limb strength	Walking, stair climbing, standing up from a chair
Inner range quadriceps 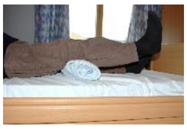	3B	Lower limb strength	Walking, stair climbing, standing up from a chair
Long arc quadriceps 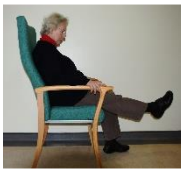	4A	Lower limb strength	Walking, stair climbing, standing up from a chair

For peer review only

Knee extension with ankle weight 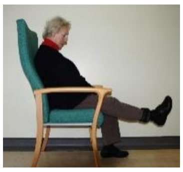	4B	Lower limb strength	Walking, stair climbing, standing up from a chair
Sit to stand using arms 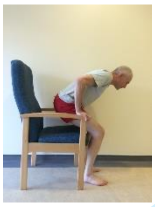	4C	Upper and lower body strength	Walking, stair climbing, standing up from a chair, lifting/carrying household objects, getting in/out of car
Sit to stand without arms 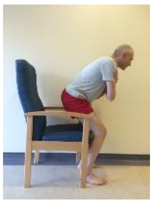	4D	Lower limb and trunk strength	Stair climbing, standing up from a chair, walking, getting in/out of car
Wall slide 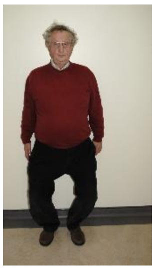	4E	Lower limb strength	Stair climbing, standing up from a chair, walking, getting in/out of car, lifting household objects
Mini Squats 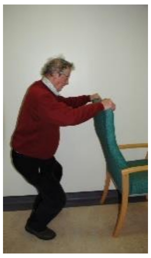	4F	Lower limb strength	Stair climbing, standing up from a chair, walking, getting in/out of car, lifting household objects

For peer review only

Single leg dip 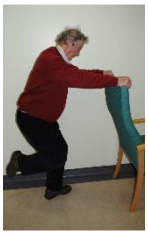	4G	Lower limb strength	Stair climbing, standing up from a chair, walking, lifting household objects
Static hamstrings 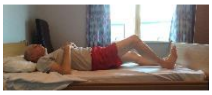	5A	Lower limb strength	Stair climbing, standing up from a chair, walking, lifting household objects
Sliding heel backwards 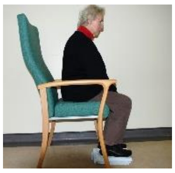	5B	Lower limb strength and range of movement	Stair climbing, standing up from a chair, walking, kneeling
Standing hamstring curls 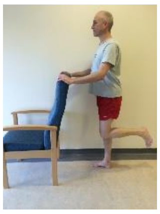	5C	Lower limb strength	Stair climbing, standing up from a chair, walking, lifting household objects
Active hamstrings 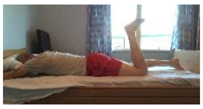	5D	Lower limb strength	Stair climbing, standing up from a chair, walking, lifting household objects
Standing hamstring curls with ankle weight 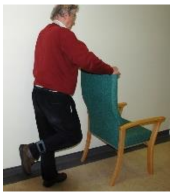	5E	Lower limb strength	Stair climbing, standing up from a chair, walking, lifting household objects
Lunge 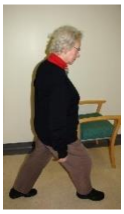	5F	Lower limb strength	Stair climbing, standing up from a chair, walking, lifting household objects

For peer review only

Hip abduction 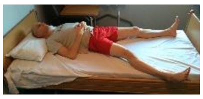	6A	Lower limb strength	Walking, dressing, getting into/out of bed
Bridging 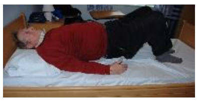	6B	Lower limb strength	Dressing, walking , standing up from a chair
Hip hitching 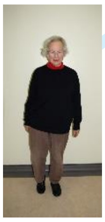	6C	Lower limb strength	Walking, dressing, getting into/out of bed
Standing hip abduction 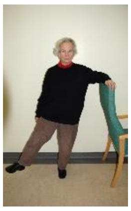	6D	Lower limb strength	Walking, dressing, getting into/out of bed, standing from a chair
Standing hip abduction with ankle weight 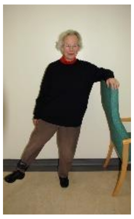	6E	Lower limb strength	Walking, dressing, getting into/out of bed, standing from a chair
Short lever hip abduction in side lying 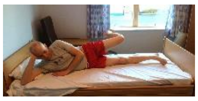	6F	Lower limb strength	Walking, dressing, getting into/out of bed
Long lever hip abduction in side lying 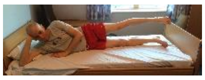	6G	Lower limb strength	Walking, dressing, getting into/out of bed
Hip abduction in side lying with ankle weight 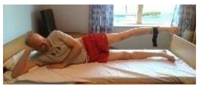	6H	Lower limb strength	Walking, dressing, getting into/out of bed

For peer review only

Bilateral calf raise 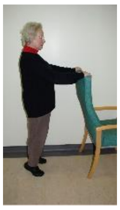	7A	Lower limb strength	Walking and stair climbing
Single calf raise 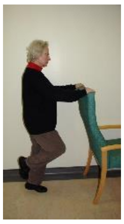	7B	Lower limb strength	Walking and stair climbing
Standing feet together 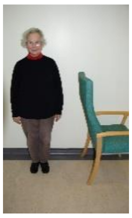	8A	Balance	Falls Prevention
Semi-tandem stance 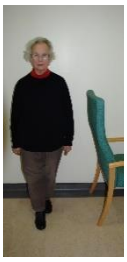	8B	Balance	Falls prevention
Tandem stance 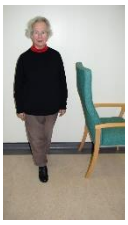	8C	Balance	Falls Prevention
Single leg stance 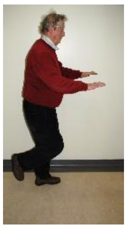	8D	Balance	Falls Prevention

For peer review only

Walking on the spot 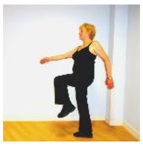	9A	Walking skills and balance	Walking and falls prevention
Waling forwards and backwards 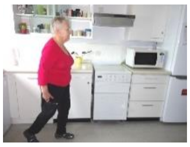	9B	Walking skills and balance	Walking a straight path and falls prevention
Side stepping 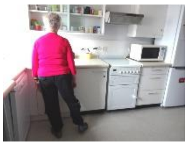	9C	Walking skills and balance	Walking, stepping around obstacles, falls prevention
Step ups 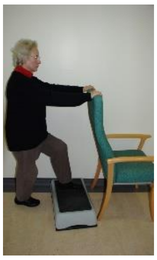	9D	Walking skills and balance	Walking, stepping onto kurbs, stair climbing, falls prevention
Mini lunge step up 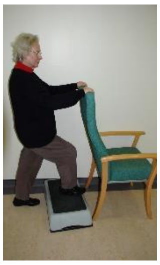	9E	Walking skills and balance	Walking, stepping onto kurbs, stair climbing, falls prevention
Turning 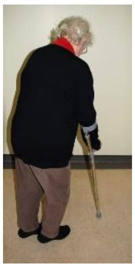	9F	Walking skills and balance	Walking with aid and falls prevention

For peer review only

Figure of 8 walk 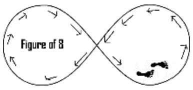	9G	Walking skills and balance	Walking both straight and curved paths, falls prevention
Tandem walking 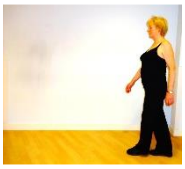	9H	Walking skills and balance	Walking and falls prevention

Usual Care

Participants randomised to usual care underwent standard in and out-patient post-operative physiotherapy as it was delivered routinely at each of the 14 research sites.

On discharge participants were given general written advice following knee arthroplasty and a home exercise programme to continue with until they were seen by an out-patient physiotherapist. To standardise usual care, participants would receive at least 1 session of physiotherapy and up to a maximum of 6 sessions which aligns with what the NHS can realistically provide. The exact duration and content of the physiotherapy sessions were at the treating physiotherapist's discretion, but in general would be between 30 and 60 minutes duration and would include a combination of range of motion and strengthening exercises, gait re-education with progression of walking aids and advice. Any differences seen in usual care across the sites is reflective of the variety of standard care delivered post knee arthroplasty across the UK.

Photographs

All participants consented to the use of their image in the treatment guide

For peer review only

CONSORT 2010 checklist of information to include when reporting a randomised trial*

Section/Topic	Item No	Checklist item	Reported on page No
**Title and abstract**			
	1a	Identification as a randomised trial in the title	1
	1b	Structured summary of trial design, methods, results, and conclusions (for specific guidance see CONSORT for abstracts)	1-2
**Introduction**			
Background and objectives	2a	Scientific background and explanation of rationale	3-4
	2b	Specific objectives or hypotheses	1 & 4
**Methods**			
Trial design	3a	Description of trial design (such as parallel, factorial) including allocation ratio	4-8
	3b	Important changes to methods after trial commencement (such as eligibility criteria), with reasons	n/a
Participants	4a	Eligibility criteria for participants	5
	4b	Settings and locations where the data were collected	6
Interventions	5	The interventions for each group with sufficient details to allow replication, including how and when they were actually administered	6
Outcomes	6a	Completely defined pre-specified primary and secondary outcome measures, including how and when they were assessed	7
	6b	Any changes to trial outcomes after the trial commenced, with reasons	n/a
Sample size	7a	How sample size was determined	7
	7b	When applicable, explanation of any interim analyses and stopping guidelines	n/a
Randomisation:			
Sequence generation	8a	Method used to generate the random allocation sequence	5
	8b	Type of randomisation; details of any restriction (such as blocking and block size)	5
Allocation concealment mechanism	9	Mechanism used to implement the random allocation sequence (such as sequentially numbered containers), describing any steps taken to conceal the sequence until interventions were assigned	5
Implementation	10	Who generated the random allocation sequence, who enrolled participants, and who assigned participants to interventions	5
Blinding	11a	If done, who was blinded after assignment to interventions (for example, participants, care providers, those	5

For peer review only

		assessing outcomes) and how	
	11b	If relevant, description of the similarity of interventions	5-6
Statistical methods	12a	Statistical methods used to compare groups for primary and secondary outcomes	6-8
	12b	Methods for additional analyses, such as subgroup analyses and adjusted analyses	6-8
**Results**			
Participant flow (a diagram is strongly recommended)	13a	For each group, the numbers of participants who were randomly assigned, received intended treatment, and were analysed for the primary outcome	9,22
	13b	For each group, losses and exclusions after randomisation, together with reasons	9,22
Recruitment	14a	Dates defining the periods of recruitment and follow-up	6
	14b	Why the trial ended or was stopped	n/a
Baseline data	15	A table showing baseline demographic and clinical characteristics for each group	Table 1
Numbers analysed	16	For each group, number of participants (denominator) included in each analysis and whether the analysis was by original assigned groups	9 & 24
Outcomes and estimation	17a	For each primary and secondary outcome, results for each group, and the estimated effect size and its precision (such as 95% confidence interval)	27-29
	17b	For binary outcomes, presentation of both absolute and relative effect sizes is recommended	n/a
Ancillary analyses	18	Results of any other analyses performed, including subgroup analyses and adjusted analyses, distinguishing pre-specified from exploratory	9
Harms	19	All important harms or unintended effects in each group (for specific guidance see CONSORT for harms)	11,30
**Discussion**			
Limitations	20	Trial limitations, addressing sources of potential bias, imprecision, and, if relevant, multiplicity of analyses	12
Generalisability	21	Generalisability (external validity, applicability) of the trial findings	11-13
Interpretation	22	Interpretation consistent with results, balancing benefits and harms, and considering other relevant evidence	11-13
**Other information**			
Registration	23	Registration number and name of trial registry	2
Protocol	24	Where the full trial protocol can be accessed, if available	4
Funding	25	Sources of funding and other support (such as supply of drugs), role of funders	2

*We strongly recommend reading this statement in conjunction with the CONSORT 2010 Explanation and Elaboration for important clarifications on all the items. If relevant, we also recommend reading CONSORT extensions for cluster randomised trials, non-inferiority and equivalence trials, non-pharmacological treatments, herbal interventions, and pragmatic trials. Additional extensions are forthcoming: for those and for up to date references relevant to this checklist, see www.consort-statement.org.

For peer review only - http://bmjopen.bmj.com/site/about/guidelines.xhtml

TIDieR Template for Intervention Description and Replication

The TIDieR (Template for Intervention Description and Replication) Checklist*:

Information to include when describing an intervention and the location of the information

Item number	Item	Where located ** Primary paper (page or appendix number)	Other † (details)
	**BRIEF NAME**		
**1.**	Provide the name or a phrase that describes the intervention.	P5 lines 126-136	Paper Ref 13
	**WHY**		
**2.**	Describe any rationale, theory, or goal of the elements essential to the intervention.	_P3 l83-89	_Ref 13 & 22_
	**WHAT**		
**3.**	Materials: Describe any physical or informational materials used in the intervention, including those provided to participants or used in intervention delivery or in training of intervention providers. Provide information on where the materials can be accessed (e.g. online appendix, URL).	______________	_Ref 13 & 22_
**4.**	Procedures: Describe each of the procedures, activities, and/or processes used in the intervention, including any enabling or support activities.	______________	__Ref 13 & 22_
	**WHO PROVIDED**		
**5.**	For each category of intervention provider (e.g. psychologist, nursing assistant), describe their expertise, background and any specific training given.	_P5 l 135-136_	______________
	**HOW**		
**6.**	Describe the modes of delivery (e.g. face-to-face or by some other mechanism, such as internet or telephone) of the intervention and whether it was provided individually or in a group.	_p5 l 126-146__	______________
	**WHERE**		
**7.**	Describe the type(s) of location(s) where the intervention occurred, including any necessary infrastructure or relevant features.	__p5 126-146_	______________

For peer review only - http://bmjopen.bmj.com/site/about/guidelines.xhtml

For peer review only

	**WHEN and HOW MUCH**		
**8.**	Describe the number of times the intervention was delivered and over what period of time including the number of sessions, their schedule, and their duration, intensity or dose.	_p5 l133-135	______________
	**TAILORING**		
**9.**	If the intervention was planned to be personalised, titrated or adapted, then describe what, why, when, and how.	______________	__Ref 13 & 22_
	**MODIFICATIONS**		
**10.‡**	If the intervention was modified during the course of the study, describe the changes (what, why, when, and how).	________n/a___	______________
	**HOW WELL**		
**11.**	Planned: If intervention adherence or fidelity was assessed, describe how and by whom, and if any strategies were used to maintain or improve fidelity, describe them.	______________	_Ref 11 & 22_
**12.‡**	Actual: If intervention adherence or fidelity was assessed, describe the extent to which the intervention was delivered as planned.	______________	_Ref 22

** **Authors** - use N/A if an item is not applicable for the intervention being described. **Reviewers** – use '?' if information about the element is not reported/not sufficiently reported.

† If the information is not provided in the primary paper, give details of where this information is available. This may include locations such as a published protocol or other published papers (provide citation details) or a website (provide the URL).

‡ If completing the TIDieR checklist for a protocol, these items are not relevant to the protocol and cannot be described until the study is complete.

* We strongly recommend using this checklist in conjunction with the TIDieR guide (see *BMJ* 2014;348:g1687) which contains an explanation and elaboration for each item.

* The focus of TIDieR is on reporting details of the intervention elements (and where relevant, comparison elements) of a study. Other elements and methodological features of studies are covered by other reporting statements and checklists and have not been duplicated as part of the TIDieR checklist. When a **randomised trial** is being reported, the TIDieR checklist should be used in conjunction with the CONSORT statement (see www.consort-statement.org) as an extension of **Item 5 of the CONSORT 2010 Statement.** When a **clinical trial protocol** is being reported, the TIDieR checklist should be used in conjunction with the SPIRIT statement as an extension of **Item 11 of the SPIRIT 2013 Statement** (see www.spirit-statement.org). For alternate study designs, TIDieR can be used in conjunction with the appropriate checklist for that study design (see www.equator-network.org).

For peer review only - http://bmjopen.bmj.com/site/about/guidelines.xhtml

BMJ Open

A home-based rehabilitation programme compared with traditional physiotherapy for patients at risk of poor outcome after knee arthroplasty: The CORKA randomised controlled trial.

Journal:	*BMJ Open*
Manuscript ID	bmjopen-2021-052598.R2
Article Type:	Original research
Date Submitted by the Author:	04-Aug-2021
Complete List of Authors:	Barker, Karen L.; University of Oxford, Nuffield Department of Orthopaedics, Rheumatology and Musculoskeletal Sciences<br>Room, Jonathan; Oxford University Hospitals NHS Foundation Trust Nuffield Orthopaedic Centre<br>Knight, Ruth; University of Oxford, Oxford Clinicial Trials Research Unit (OCTRU); Centre Statistics in Medicine<br>Dutton, Susan; University of Oxford, Oxford Clinical Trials Research Unit (OCTRU); Centre statistics in Medicine<br>Toye, Francine; Oxford University Hospitals NHS Foundation Trust Nuffield Orthopaedic Centre, Nuffield Orthopaedic Centre Physiotherapy Research Unit<br>Leal, Jose; University of Oxford, Nuffield Department of Population Health<br>Kenealy, Nicola; Oxford University, Nuffield Department of Orthopaedics, Rheumatology and Musculoskeletal Sciences<br>Maia Schlüssel, Michael; University of Oxford, Nuffield Department of Orthopaedics, Rheumatology and Musculoskeletal Sciences<br>Collins, Gary; University of Oxford, Centre for Statistics in Medicine<br>Beard, David; University of Oxford, Nuffield Dept of Orthopaedics, Rheumatology and Musculoskeletal Sciences<br>Price, Andrew; University of Oxford, Nuffield Department of Orthopaedics, Rheumatology and Musculoskeletal Sciences<br>Underwood, Martin; Warwick University, Warwick Medical School<br>Drummond, Avril; University of Nottingham, Div of Rehabilitation and Ageing<br>Lamb, Sarah; University of Exeter, College of Medicine and Health; University of Oxford, NDORMS
**Primary Subject Heading**:	Evidence based practice
Secondary Subject Heading:	Rehabilitation medicine
Keywords:	Adult orthopaedics < ORTHOPAEDIC & TRAUMA SURGERY, Knee < ORTHOPAEDIC & TRAUMA SURGERY, REHABILITATION MEDICINE

For peer review only - http://bmjopen.bmj.com/site/about/guidelines.xhtml

I, the Submitting Author has the right to grant and does grant on behalf of all authors of the Work (as defined in the below author licence), an exclusive licence and/or a non-exclusive licence for contributions from authors who are: i) UK Crown employees; ii) where BMJ has agreed a CC-BY licence shall apply, and/or iii) in accordance with the terms applicable for US Federal Government officers or employees acting as part of their official duties; on a worldwide, perpetual, irrevocable, royalty-free basis to BMJ Publishing Group Ltd ("BMJ") its licensees and where the relevant Journal is co-owned by BMJ to the co-owners of the Journal, to publish the Work in this journal and any other BMJ products and to exploit all rights, as set out in our licence.

The Submitting Author accepts and understands that any supply made under these terms is made by BMJ to the Submitting Author unless you are acting as an employee on behalf of your employer or a postgraduate student of an affiliated institution which is paying any applicable article publishing charge ("APC") for Open Access articles. Where the Submitting Author wishes to make the Work available on an Open Access basis (and intends to pay the relevant APC), the terms of reuse of such Open Access shall be governed by a Creative Commons licence – details of these licences and which Creative Commons licence will apply to this Work are set out in our licence referred to above.

Other than as permitted in any relevant BMJ Author's Self Archiving Policies, I confirm this Work has not been accepted for publication elsewhere, is not being considered for publication elsewhere and does not duplicate material already published. I confirm all authors consent to publication of this Work and authorise the granting of this licence.

For peer review only

A home-based rehabilitation programme compared with traditional physiotherapy for patients at risk of poor outcome after knee arthroplasty: The CORKA randomised controlled trial.

Karen L. Barker [1,2] *, Jon Room[1,2], Ruth Knight[3], Susan J. Dutton[3], Fran Toye[2], Jose Leal[4], Nicola Kenealy[1], Michael Maia Schlüssel[3], Gary Collins[3], David J. Beard[1], Andrew Price[1], Martin Underwood[5], Avril Drummond[6], Sarah E Lamb[1,7,] on behalf of CORKA Trial group.

1 Nuffield Department of Orthopaedics, Rheumatology and Musculoskeletal Sciences, University of Oxford, Oxford, UK.

2 Physiotherapy Research Unit, Nuffield Orthopaedic Centre, Oxford University Hospitals NHS Foundation Trust, Oxford, UK.

3 Oxford Clinical Trials Research Unit (OCTRU), Centre for Statistics in Medicine, Nuffield Department of Orthopaedics, Rheumatology and Musculoskeletal Sciences, University of Oxford, Oxford, UK.

4 Health Economics Research Centre, Nuffield Department of Population Health, University of Oxford, Oxford, UK.

5 Warwick Clinical Trials Unit, Warwick Medical School, University of Warwick, Coventry, UK

6 School of Health Sciences, University of Nottingham, Nottingham, UK

7 School of Medicine and Health, University of Exeter

*Corresponding Author Email karen.barker@ouh.nhs.uk.

Physiotherapy Research Unit

Nuffield Orthopaedic Centre

Windmill Rd, Oxford OX3 7LD

Tel no: 01865 738080

Keywords: knee arthroplasty, physical therapy, rehabilitation

Word Count: 4143

Abstract

Objectives: To evaluate whether a home-based rehabilitation programme for people assessed as being at risk of a poor outcome after knee arthroplasty offers superior outcomes to traditional out-patient physiotherapy.

Design: A prospective, single-blind, two-arm randomised controlled superiority trial.

Setting: 14 National Health Service (NHS) physiotherapy departments in the UK.

Participants: 621 participants identified at high risk of a poor outcome after knee arthroplasty using a bespoke screening tool.

Interventions: A multi-component home-based rehabilitation programme delivered by rehabilitation assistants with supervision from qualified therapists versus usual care out-patient physiotherapy.

Main outcome measures: The primary outcome was the Late Life Function and Disability Instrument (LLFDI) at 12 months. Secondary outcomes were the Oxford Knee Score (OKS; a disease-specific measure of function), Knee injury and Osteoarthritis Outcome Score (KOOS) Quality of Life subscale, Physical Activity Scale for the Elderly (PASE), EQ-5D-5L, and physical function assessed using the Figure of 8 Walk test, 30 Second Chair Stand Test (30SCST), and Single Leg Stance.

Results: 621 participants were randomised between March 2015 and January 2018. 309 were assigned to CORKA home-based rehabilitation, receiving a median 5 treatment sessions (IQR 4-7). 312 were assigned to usual care, receiving a median 4 sessions (IQR 2-6). The primary outcome, LLFDI function total score at 12 months, was collected for 279 participants (89%) in the home-based CORKA group and 287 participants (92%) in the usual-care group. No clinically or statistically significant difference was found between the groups (intention-to-treat adjusted

For peer review only

For peer review only - http://bmjopen.bmj.com/site/about/guidelines.xhtml

difference = 0.49 points; 95% confidence interval -0.89 to 1.88; p = 0.48). There were no statistically significant differences between the groups on any of the patient-reported or physical secondary outcome measures at 6 or 12 months.

There were 18 participants in the intervention group reporting a serious adverse event (5.8%), only one directly related to the intervention., all other adverse events recorded throughout the trial related to underlying chronic medical conditions.

Conclusions: The CORKA intervention was not superior to usual care. The trial detected no significant differences, clinical or statistical, between the two groups on either primary or secondary outcomes. CORKA offers an evaluation of an intervention utilising a different service delivery model for this patient group.

Trial Registration: ISRCTN 13517704

Funding: National Institute for Health Research Health Technology Assessment programme (HTA 12/196/08)

Article Summary

Strengths and limitations of this study:

- Large sample size and low loss to follow up which ensured sufficient numbers to make the study sufficiently powered to detect any between group differences.
- The trial was pragmatic and utilised an innovative workforce model that could readily translate into NHS practice within existing commissioning and financial constraints.
- The study offers an alternative service delivery model in line with the latest UK guidance from NICE.
- Limitations are that the screening tool did not identify patients that were a good fit of risk of poor outcome for the intervention that we developed.
- Another limitation, common to all trials of post operative physiotherapy after knee arthroplasty was the absence of a no physiotherapy treatment control group (as post

For peer review only

discharge physiotherapy is a core component of UK arthroplasty practice it is not racticable to recruit to a no treatment comparator especially for those identified as being at higher risk of a poor outcome.

Introduction

Knee Arthroplasty operations in the UK continue to rise with the National Joint Registry reporting over 100,000 in 2017 alone (1). This is an increase of over 3% from 2010. The population receiving surgery is getting older, frailer and with more co-existing health conditions. Data from patient-reported outcome measures (PROMs) have shown that most patients achieve a satisfactory outcome. However, between 10% and 15% are unsatisfied or report little or no improvement after surgery (2). We do not yet know how to identify these patients or target rehabilitation to improve their outcomes.

Physiotherapy provision in the UK varies following discharge from hospital after KA but typically patients will continue to have rehabilitation needs to help restore muscle strength and endurance, range of motion, walking distance, and performance of higher-level functional activities. Patients are commonly referred for further physiotherapy in an out-patient setting to assist with these issues. The timing of this referral varies, with some hospitals advocating immediate commencement of therapy after discharge from hospital and others waiting until the first post-operative review at around 6 weeks before considering referral. This typically involves between 4 and 6 sessions of physiotherapy in an out-patient clinic setting. Patients do not routinely receive Occupational Therapy input following knee arthroplasty surgery unless they are highlighted to have particular difficulties with their daily activities or have specific barriers to discharge home. Concern has been raised that many exercise programmes lack adequate intensity to enable optimal recovery (3,4). Internationally, where much longer courses of physiotherapy are often provided, research has indicated that 12-18 hours of physiotherapy (5) or a mean of 17 visits (6) may be needed to produce a benefit. These levels of care are substantially higher than those provided in the UK and, in the current economic climate, may be more than the National Health Service (NHS) can afford.

For peer review only

Given the rising number of operations, the relatively limited therapy resources available, and the increasing age and frailty of patients receiving this surgery, it is important that rehabilitation resources are concentrated on those patients who need the most help to achieve a good outcome. Not all patients need post-operative out-patient physiotherapy and some will make a full recovery using self-directed rehabilitation (7–10). Current rehabilitation strategies do not meet the needs of all patients, particularly those who are socially isolated, do not have easy access to transport, and are frail (11). It is thus pertinent to focus our resources on those who are most likely to be at risk of a poor outcome, least likely to be able to engage with a self-management approach, and able to benefit most from rehabilitation input To maximise access to therapy and to provide this within a similar cost envelope to current practice we designed an intervention to be delivered in the patients' homes utilising a workforce model that utilise rehabilitation assistants supported by qualified therapy staff, drawing on the success of this model in delivering therapy programmes such as the OTAGO falls prevention intervention (12). This trial was carried out to determine if a targeted home-based intervention for those at risk of poor outcome, delivered by a mix of qualified staff and rehabilitation assistants, offers superior outcomes in patient reported and physical function at 1 year following KA compared to traditional out-patient physiotherapy.

Methods

The CORKA trial was a multicentre prospective, single blinded, two-arm randomised controlled superiority trial with assessment for the clinical outcomes at baseline, 6 and 12 months. The study was carried out according to the published protocol (13).

Recruitment

Participants were recruited from 14 NHS Trusts across England. Patients aged 55 years and over due to undergo knee arthroplasty were sent information concerning the trial prior to their pre-operative assessment appointment. Those expressing interest were screened in the clinic using a screening tool developed for the trial. This aimed to identify patients at risk of a poorer outcome based on data collected as part of standard pre-operative assessment screening. The

For peer review only

tool was developed using all data from the Knee Arthroplasty Trial dataset and further details of its development has been previously published (14). The screening tool had a range of possible scores from 0 – 10, patients with a score of 5 or more were classified as being at increased risk of a poor outcome after 1 year defined as an Oxford Knee Score of 26 or less. [Appendix A]. The performance of the model was fair, as measured by its discrimination. Participants who were eligible were screened again post-operatively to confirm their eligibility and consent. All participants provided written informed consent. Exclusion criteria included any major peri-operative complications, further surgery planned within the next 12 months and absolute contraindications to exercise. Screening was performed by research clinicians at the local sites, and screening logs recorded the reasons for ineligibility and why eligible patients declined to participate.

Randomisation

Randomisation took place on the third post-operative day, or on hospital discharge if earlier. Should any participant have experienced a severe medical peri-operative event they would not be entered into the trial but continue with usual care as able. Participants were randomly allocated by a web-based randomisation system to either 'usual care' or 'home-based rehabilitation programme' in a 1:1 ratio using permuted blocks of various sizes (sizes 2, 4 and 6 in a 1:2:1 ratio, that is on average there were twice as many blocks of size 4 than either size 2 or size 6) to prevent prediction of treatment allocation, and this was stratified by recruitment site. Once randomised, participants and those delivering the rehabilitation were aware of the treatment allocation due to the nature of the intervention. Those carrying out follow-up outcome measurements remained blinded to treatment allocation.

Interventions

<u>CORKA home-based intervention</u>

The CORKA intervention started up to 4 weeks after surgery, with most participants seen within 2 weeks. In the period between surgery and the initial appointment participants continued with

For peer review only

a home exercise programme given to them by ward physiotherapists in line with standard current practice.

The CORKA home-based intervention is a multicomponent rehabilitation programme developed in collaboration with clinicians and patients; it has been described in detail in a previous publication (15). It consisted of an initial assessment appointment and up to 6 follow-up sessions. Its aim was to improve the function and participation in activities of participants at risk of a poor outcome after surgery. The primary component of the rehabilitation programme was an individually adapted exercise package conducted in participants' own homes. During the participants' initial assessment, the physiotherapist focused on tasks that were identified as being problematic. The exercise programme was then tailored to the individuals' needs and goals. If an exercise was felt to be unsafe for any particular participant, therapists could omit it and substitute with an additional exercise from the package; or utilise a more stable starting position. Additional components consisted of functional task practice, adherence approaches such as utilisation of techniques such as goal setting and exercise dairies. If required, they were provided with appropriate aids and equipment,

The physiotherapist undertook the initial assessment and prescription of the exercise programme, observed by the rehabilitation assistant. For future sessions the rehabilitation assistant continued the programme and modified as required using treatment algorithms and decision aids to progress. The physiotherapist undertook one further session in the middle of the programme to review the participant's progress and their exercise programme. The way this was operationalised is outlined in Appendix B.

All therapists delivering the CORKA intervention received a 2-3 hour training session which included instructions on how to assess and treat CORKA participants, prescribe and progress the different categories within the exercise programme.

<u>Usual care</u>

Those allocated to the usual-care arm received standard post-operative physiotherapy. Usual care after surgery could vary considerably across the trial's different UK locations. However, it was highly likely that usual care would include several of the following: between 1 and 6 sessions of physiotherapy in an out-patient setting, class-based setting, or hydrotherapy;

For peer review only

written advice on home exercises on discharge from hospital; and an assessment of any potential home requirements or barriers to discharge by an occupational therapist. To standardise usual care as much as possible, participants were expected to attend a minimum of 1 session and a maximum of 6 sessions of usual care physiotherapy.

Quality assurance checks took place at all CORKA research sites. They included fidelity checks during which assessment and treatment delivery were observed. All aspects of the intervention were checked against a pre-defined fidelity checklist created in line with the study protocol.

Outcomes

Baseline data were collected face-to-face no more than four weeks before surgery. Follow-up data collection was by face-to-face clinical assessments at 6 and 12 months following randomisation. Where face-to-face assessment was not possible, postal and telephone data collection methods were used to obtain self-reported core data. Baseline data included the Functional Co-morbidities index to provide baseline characteristics of the participants.

Primary outcome

The primary outcome was the Late Life Function and Disability Instrument (LLFDI) overall function score. It was developed specifically to assess change in community-dwelling older adults. It assesses and responds to meaningful change in two distinct outcomes: a person's ability to do discrete actions or activities using a 32-item *function* component (Primary outcome) and a person's performance of socially defined life tasks using a 16-item *disability* component (secondary outcome).

Secondary outcomes

Secondary outcomes consisted of self-reported and physical measures. The self-reported measures were the Oxford Knee Score (OKS), Physical Activity Scale for the Elderly (PASE), Knee Injury and Osteoarthritis Outcome Score (KOOS) Quality of Life subscale, and EQ-5D-5L questionnaires. The physical measures were the Figure of 8 Walk Test (F8WT), 30 Second Chair

For peer review only

Stand Test (30SCST), and Single Leg Stance (SLS). A health resource diary collected the exercises undertaken, medication taken, use of healthcare services and personnel, and falls.

Analysis

A detailed statistical analysis plan has previously been published (16). The LLFDI score was chosen as a clinically relevant outcome for the population and the sample size calculation was therefore based on this variable. As there is no formal minimally clinically important difference for the LLFDI the calculation was based on a moderately small standard effect size of 0.275 which gave a sample of 620 participants (310 per arm) with 90% power, 5% (two-sided) significance, allowing for 10% loss to follow up.

Two analysis populations were considered: the intention-to-treat (ITT) population and the per-protocol (PP) population. The ITT population included all randomised participants analysed according to their allocated intervention. The PP population included only participants who received at least one session of their allocated intervention, did not receive more treatment than intended (more than 6 sessions of usual care or 7 sessions of home-based rehabilitation), and provided follow-up data.

LLFDI function scores at 6 and 12-months post-randomisation were summarised by treatment group and analysed using a linear mixed effects model with repeated measures adjusted for baseline score and recruiting site (stratification factor). Time was treated as categorical and an interaction between the outcome measurement time point and randomised group was included to allow the treatment effect to be estimated at each time point, reported as the adjusted mean difference in LLFDI between groups with 95% CI and associated p-value. The underlying assumptions of this model were assessed. Twelve months post-randomisation was considered the primary endpoint. The primary analysis was performed for the ITT population using multiple imputation (MI) to impute missing data. MI was performed separately for each treatment group and the model included type of knee replacement (TKR vs. UKR), recruiting centre, gender, whether or not the participant had had previous lower limb surgery, Charnley

For peer review only

ABC score and current mobility support used. As sensitivity analyses this was repeated for the ITT population using available cases, the PP population using available cases and using a complier average causal effect (CACE) analysis (17).

Linear mixed effects models with repeated measures, similar to that described for the primary outcome, were used to analyse each of the secondary PROMs (LLFDI disability limitation, LLFDI disability frequency, OKS, KOOS QoL, PASE, EQ-5D- 5L utility and VAS), 30SCST (number of stands), and F8WT (time and steps). Differences between the two groups in SLS times were compared using a Wilcoxon rank-sum test since the assumptions of the linear mixed effects model were not satisfied. All of these analyses were performed for the ITT population using available cases only. For the key secondary outcomes (LLFDI disability limitation, LLFDI disability frequency, OKS, and KOOS QoL), these analyses were repeated for the ITT population using multiple imputation (imputation model as outlined for the primary outcome) and the PP population using available cases.

The number and proportion of SAEs were reported by treatment group, as well as the number and proportion of participants experiencing an SAE. The rates were compared across treatment groups.

Additional Analysis

An economic analysis and nested qualitative analysis were conducted as part of this study. Both economic and qualitative analysis will be reported elsewhere.

Patient and Public Involvement

The study was developed with a group of service users that identified concern about the access to rehabilitation for older more frail patients after knee arthroplasty. The intervention was developed with active involvement of PPI stakeholders who worked alongside us to develop the intervention. A PPI representative collaborated on the application for funding and was a full

For peer review only

voting member of the trial steering group which benefitted from their expertise and active engagement throughout the period of the study.

Results

The CORKA recruited participants between March 2015 and January 2018.2,788 patients were screened for eligibility. and 621 participants were randomised, 312 to usual care and 309 to the home based rehabilitation programme. A CONSORT flow chart summarises the flow of participants through the trial including details on the number of participants randomised and the numbers allocated to and receiving at least 1 session of each treatment (See Figure 1). Of the 621 participants, 34 (5.5%) withdrew from follow-up during the course of the trial. Three participants died between the 6 and 12 months follow ups.

The mean age of participants was 70.4 (SD 8.07), 59.7% were female and mean BMI was 31.5 (SD 4.74). Most participants scored 5 or 6 on the screening tool (79.5%) with 5 being the minimum score needed to be eligible for the study and indicating a risk of poor outcome. The baseline characteristics of the participants is summarised in Table 1.

Participants were defined as complying with usual care if they attended at least 1 treatment session and for home-based rehabilitation at least 4 treatment sessions. We used different thresholds for the two treatments as usual care varied considerably between sites including as little as one session. Therefore, compliance in this arm was defined as participants receiving at least one session. For the intervention arm, it was pre-specified that at least 4 of the 7 planned sessions were required to be compliant. Therapy compliance was 95.2% in usual care and 87.1% for the CORKA intervention. The median number of sessions for usual care was 4 (IQR 2,6) and for the CORKA intervention was 5 (IQR 4,7).

Primary outcome analysis

For peer review only

For the ITT analysis using multiple imputation there was no statistically significant difference between the two treatment groups at the primary time point of 12 months post-randomisation (adjusted difference 0.49, 95% CI: -0.89 to 1.88) or at 6 months (adjusted difference 0.66, 95% CI: -0.70, 2.03). This equates to a standardised effect size of 0.03 at 12 months.

Trends in LLFDI function scores over time are plotted for each treatment group in Figure 2. There was a substantial improvement between baseline and 6 months in both groups, with minimal additional difference between 6 and 12 months post-randomisation.

None of the sensitivity analyses conducted (ITT using available cases, per-protocol analysis, CACE analysis) demonstrated a different result.

Secondary outcome analysis

For the secondary PROMS ITT analysis using available cases, no significant differences between the two treatment groups at either time point (6 and 12 months) was identified for any of the secondary outcomes (see Table 2). The sensitivity analyses of the key secondary PROMS identified only one difference, of borderline statistical significance, for the LLFDI disability limitation at 6 months using the ITT population and multiple imputation (adjusted difference 2.67, 95% CI: 0.14 to 5.19,).

The physical measures are summarised by treatment group over time in Table 3. With the exception of one of the SLS tests, there were no differences in change in performance between the groups on the physical measures.

The total number of SAEs experienced in this trial was low; 18 events in 14 participants in the usual care group (4.5%) and 20 events in 18 participants in the CORKA intervention group (5.8%), experienced. Adverse events were independently reviewed and categorised using the System Organ Classes codes and as related to the intervention or not. For all SAEs only one was classified as 'possibly related'. In total there were 7 SAEs in the musculoskeletal and connective tissue disorder category, with one categorised as related to the treatment interventions (Table

For peer review only

4). The majority of the SAEs were related to medical conditions unconnected with the participant's surgery and were classified into categories such as cardiac disorders, renal and urinary disorder, or immune system disorders. There was not a significant difference in the number of participants experiencing an SAE between the two groups (Risk difference 1.3%, 95% CI -2.1% to 4.8%).

Discussion

No statistical or clinically meaningful differences were found between CORKA and usual out-patient physiotherapy. Outcomes across a range of measures were closely matched irrespective of which of the two interventions was received. The use of a workforce model using rehabilitation assistants offered flexibility of staffing and did not result in any increase in adverse events.

Strengths and limitations of the study

Strengths of this study include the large sample size and low loss to follow up which ensured sufficient numbers to make the study sufficiently powered to detect any between group differences. The trial was pragmatic and utilised an innovative workforce model that could readily translate into NHS practice within existing commissioning and financial constraints. The study offers an alternative service delivery model in line with the latest UK guidance from NICE. Limitations are that the screening tool did not identify patients that were a good fit of risk of poor outcome for the intervention that we developed. The screening tool has not been validated; but uses items that are used by many knee arthroplasty trials to determine entry, these are broad and inclusive, reflecting common clinical practice. Recent recommendations from the UK National Institute for Health and Clinical Excellence noted that it would be useful if a tool existed that indicated those who would benefit from supervised rehabilitation and recognised that there is a significant proportion of patients to whom this will apply (18). In developing our screening tool for the trial, we attempted just this, but recognise that there was a poor match between the patients screened and the intervention that we developed. Many of

For peer review only

our participants were borderline high risk scoring just above the cut-point of 5, resulting in fewer participants at higher risk than anticipated which may have affected the results. Another limitation, common to all trials of post-operative physiotherapy after knee arthroplasty, was the absence of a control group. As out-patient physiotherapy remains an accepted and expected core component of UK arthroplasty practice it was not deemed ethical or practicable to attempt to recruit to a no treatment comparator in a group who had already been identified as at higher risk of a poor outcome. As with most physiotherapy trials it was not feasible to blind patients or therapists to treatment allocation. We also chose to deliver an intervention that met current commissioning guidance of around six sessions. It is arguable that more intensive treatment as is common in other countries may have been more effective, but this is not supported by studies using longer interventions (19-24).

We chose to use the LLFDI as our primary outcome over a more knee focused outcome as it is designed to map to the WHO ICF and assess change in community dwelling older adults encompassing the range of activities relevant for older, potentially frailer patients after arthroplasty. At the time of designing the trial the MCID was not published. Subsequently, Beauchamp et al reported that substantial changes are reflected in a 5-point change in the overall function scale (25). We found substantial changes from baseline to 1 year within both groups, but no between group effect. The CORKA home-based intervention showed an overall change of 8.9 points and usual care 8.7 points, indicating that the combined effect of surgery and rehabilitation unsurprisingly produced a highly significant improvement in function. Most of this improvement was probably due to the surgical procedure, but we cannot disaggregate the contribution of physiotherapy to quantify the contribution of one without the other. We found no significant between group difference. It is arguable that a more specific outcome measure targeted at knee arthroplasty may have been a better choice. We included the OKS as a secondary measure to address this potential limitation. The results of the LLFDI and OKS were well-matched.

Implications for patients and policy

For peer review only

The benefit of post-operative physiotherapeutic interventions are poorly established. A recent evidence review by NICE, of all patients receiving arthroplasty surgery, found no clinically important differences in quality of life, PROMS or functional outcomes between group or individually based supervision and self-directed rehabilitation. They recommended self-directed rehabilitation as an acceptable option for people who are well enough and have personal circumstances that make it possible; but recognised that there are likely to be subgroups who will benefit from a more supervised rehabilitation programme. The CORKA trial is one of few trials to specifically target individuals who are at risk of poor outcome post surgery. The TRIO trial (26) also targeted physiotherapy at patients considered at risk of poor outcome, based upon Oxford Knee Score at 6 weeks. Similar to this study they found no statistical differences between the two treatment arms of outpatient physiotherapy and a single physiotherapy review and home exercise based regimen.

Although we screened for patients deemed at risk of poor outcome the OKS of 37.8 points was higher than the reported average for all knee arthroplasty patients at one year of 36 points.. The TRIO trial similarly found their results, although a little lower, were similar to the unselected average (26). Most of this improvement was probably due to the surgical procedure, but we cannot disaggregate the contribution of physiotherapy to quantify the contribution of one without the other.

The CORKA intervention was based on pre-operative outcome measures, with a home-based rehabilitation programme. There may also be benefits to home supervised rehabilitation that are not captured by the current evidence or the outcome measures used in existing studies. For example, participants in our embedded qualitative study were very positive about the CORKA home-based intervention and the supervision provided by the therapists and rehabilitation assistants (27). The CORKA home-based intervention could also benefit, for example, those too frail to travel a significant distance to a larger hospital. Using the CORKA combination of qualified therapists and rehabilitation assistants might be a practical option for certain remote areas of the UK or countries that have large geographical areas not covered by therapists. This

For peer review only

suggests there is an argument for choice, according to personal preference or the practicalities of service provision.

It also addresses the workforce shortage issue by using an innovative workforce model of advanced rehabilitation assistants, moving UK service provision closer to that which has been proven to be effective in North America, where the use of physical therapy assistant graded staff is well embedded. This is a particular concern given both the projected increased need for joint arthroplasty over the next decade to accommodate an ageing population and the pressure of potential reductions in NHS funding. Evaluating the value of treatment modalities offered to these patients is crucial because many more patients are being discharged home earlier from the acute setting leaving less time available for acute physical recovery, rehabilitation, and education in hospital, increasing the potential burden of care for these patients and their families.

Future research

Further research should focus on developing a screening tool that is more sensitive in identifying those patients who will benefit from additional input beyond self-directed rehabilitation as recommended by the NICE guidance on primary joint replacement (18).

The CORKA home-based intervention was delivered by rehabilitation assistants supervised by qualified therapists in a ratio of 5:2 sessions. Given the workforce challenges in the NHS further work looking at different workforce models and interventions solely using rehabilitation assistants should be explored.

Conclusions

This large randomised controlled trial found no important differences in outcomes when rehabilitation was delivered using either a home-based, rehabilitation assistant delivered

For peer review only

rehabilitation package or a traditional out-patient physiotherapy model. Home based rehabilitation was feasible but did not confer benefits over standard out patient physiotherapy.

Contributorship Statement:

Karen L Barker (Professor of Physiotherapy) – Chief Investigator, led funding application, trial conception and design, development of interventions, supervision, and writing and reviewing report.

Jonathan Room (Research Physiotherapist) – Principal Investigator, development of intervention, and writing and reviewing report.

Ruth Knight (Statistician) – Conducting statistical analysis and writing and reviewing report.
Seamus Kent (Health Economist) – Conducting economic analysis and writing and reviewing report.

Susan Dutton (Statistician) – Designing and conducting statistical analysis and writing and reviewing report.

Fran Toye (Qualitative lead) -, conducting interviews and analysis, and writing chapter for report.

Jose Leal (Health Economist) – Designing and conducting economic analysis, supervision, and writing and reviewing report.

Nicola Kenealy (Trial Manager) – Trial management and writing and reviewing report.

For peer review only

Michael Maia Schlussel (Statistician) – Screening tool development and writing for report.

Gary Collins (co-applicant) – Supervised screening tool development.

David Beard (co-applicant) – Clinical trials expertise and contributed to report.

Andrew Price (co-applicant) – Surgical expertise and contributed to report.

Martin Underwood (co-applicant) – Primary care expertise and contributed to report.

Avril Drummond (co-applicant) – Occupational therapy expertise and contributed to report.

Sarah Lamb (co-applicant) – Clinical trials expertise, key member of trial management group, and writing and reviewing report.

Acknowledgements:
The authors would like to acknowledge the site principal investigators and research clinicians

Leon Palmer-Wilson, Ana Glennon (Ashford Hospital), Helen Wilson (Countess of Chester Hospital), Christian Brookes, Denise Hill (Dorset County Hospital), Susan Dowdle, Hazel Burt (Dorset HealthCare Hospital), Jane Harrison (Epsom Hospital), Sarah Adcock, Justine Theaker (Manchester Royal Infirmary), Gladys Nadar Arulmani (Medway Community Hospital), Sunil Jain (Medway Maritime Hospital), Mike Reed (North Tyneside General Hospital), Jonathan Room (Oxford University Hospitals), Genevieve Simpson, Gemma Knight (Royal United Hospitals Bath), Tricia Monroe (Moorgreen Hospital), Gareth Stephens, Sarah Rich (Royal Orthopaedic Hospital Birmingham).

Funding support: This trial was funded by the National Institute for Health Research Health Technology Assessment programme (HTA 12/196/08)

For peer review only

Conflict of Interest:

G Collins is a member of the HTA Commissioning Board

A Price has a consultancy with Zimmer Biomet, outside the submitted work

M Underwood has grants from NIHR, during the conduct of the study; grants from NIHR, Personal fees from NIHR, Personal fees from NICE, non financial support from Stryker PLC, grants from SERCO ltd.

S Lamb has grants from NIHR Health Technology Assessment Programme during the conduct of the study and is a member of the following Boards:

HTA Additional Capacity Funding Board 2010-2015
HTA Clinical Trials Board 2010-2015
HTA End of Life Care and Add on Studies 2015-2015
HTA Funding Boards Policy Group (Formally CSG) 2010-2015
HTA MNCH Methods Group 2013-2015
HTA Post-board funding teleconference (PG members to attend) 2010-2015
HTA Primary Care Themed Call board 2013-2014
HTA Prioritisation Group 2014-2015
NIHR CTU Standing Advisory Committee 2012-2016

Ethical approvals

The study protocol was approved by the South-Central Research Ethics Committee (Reference 15/SC/0019). Ethics permission was obtained for all participating sites.

Data availability statement

Deidentified individual participant data will be available on reasonable request to the Chief Investigator. Data will be available beginning 9 months and ending 36 months following article publication to investigators who provide a methodologically sound proposal. Proposals may be

For peer review only

submitted up to 36 months following article publication. To gain access data requestors will need to sign a data access agreement and the study in question.

Figure Caption

Figure 1 - CONSORT flow chart

Figure 2 - The LLFDI function score from baseline to 12 months post randomisation for each treatment group

References

1. National Joint Registry for England, Wales NI and the I of M. 15th Annual Report 2018. www.hqip.org.uk/wp-content/uploads/2018/11/NJR-15th-Annual-Report-2018. Accessed july 2021.
2. Jones CA, Beaupre LA, Johnston DWC, Suarez-Almazor ME. Total Joint Arthroplasties: Current Concepts of Patient Outcomes after Surgery. Rheumatic Disease Clinics of North America. 2007;33(1):71-86..
3. Naylor J, Harmer A, Fransen M, Crosbie J, Innes L. Status of physiotherapy rehabilitation after total knee replacement in Australia. Physiother Res Int. 2006;11(1):35-47.
4. Westby MD, Kennedy D, Jones DL, Jones A, Doyle-Waters M, Backman C. Post-acute physiotherapy for primary total knee arthroplasty. Cochrane Database of Systematic Reviews. 2008 (2).
5. Moffet H, Collet JP, Shapiro SH, Paradis G, Marquis F, Roy L. Effectiveness of intensive rehabilitation on functional ability and quality of life after first total knee arthroplasty: A single-blind randomized controlled trial. Arch Phys Med Rehabil. 2004;85:546-56.
6. Petterson SC, Mizner RL, Stevens JE, Raisis LEO, Bodenstab A, Newcomb W, et al. Improved function from progressive strengthening interventions after total knee arthroplasty: A randomized clinical trial with an imbedded prospective cohort. Arthritis Care Res. 2009;61 (2):174-83.
7. Rajan RA, Pack Y, Jackson H, Gillies C, Asirvatham R. No need for outpatient physiotherapy following total knee arthroplasty: A randomized trial of 120 patients. Acta Orthop Scand. 2004;75 91):71-3.;
8. Piva SR, Gil AB, Almeida GJM, DiGioia AM, Levison TJ, Fitzgerald GK.. A balance exercise program appears to improve function for patients with total knee arthroplasty : a randomized clinical trial.Phys Ther 2010;90: 880-94.
9. Artz N, Dixon S, Wylde V, Marques E, Beswick AD, Lenguerrand E, et al. Comparison of group-based outpatient physiotherapy with usual care after total knee replacement: A feasibility study for a randomized controlled trial. Clin Rehabil. 2017;31 (4):487-99.
10. Fillingham YA, Darrith B, Lonner JH, Culvern C, Crizer M, Della Valle CJ. Formal Physical Therapy May Not Be Necessary After Unicompartmental Knee Arthroplasty: A Randomized Clinical Trial. J Arthroplasty. 2018;33 (7s): S93.
11 Judge A, Arden NK, Cooper C, Kassim Javaid M, Carr AJ, Field RE, et al. Predictors of

For peer review only

outcomes of total knee replacement surgery. Rheumatology (Oxford). 2012;51(10):1804-13

12. Shubert TE, Smith ML, Goto L, Jiang L, Ory MG. Otago Exercise Program in the United States: Comparison of 2 Implementation Models. Phys Ther. 2017;97(2):187-97.

13. Barker KL, Beard D, Price A, Toye F, Underwood M, Drummond A, Collins G, Dutton S, Campbell H, Kenealey N, Room J, Lamb SE. Community-based rehabilitation after knee arthroplasty (CORKA): study protocol for a randomised controlled trial. Trials 2016 Oct 13;17(1):501.

14. Schlussel M, Collins G, Dutton S, Barker K. Development of the CORKA trial screening tool for identifying patients at increased risk of poor outcome following knee replacement. Trials. 2017;18:S1, P430.

15. Room J, Batting M, Barker KL. Development of a functional rehabilitation intervention for post knee arthroplasty patients: community based Rehabilitation post Knee Arthroplasty (CORKA) trial. Physiotherapy. 2020 Mar;106:52-64.

16. Vadher K, Knight R, Barker KL, Dutton SJ. Community based rehabilitation after knee arthroplasty (CORKA): statistical analysis plan for a randomised controlled trial. Trials. 2018 Nov 19;19(1):638

17. Dunn G, Maracy M, Tomenson B. Estimating treatment effects from randomized clinical trials with noncompliance and loss to follow-up: the role of instrumental variable methods. Stat Methods Med Res. 2005;14(4):369-95.

18. National Institute for Health and Care Excellence. Joint replacement (primary): hip, knee and shoulder [R] Evidence review for outpatient hip and knee postoperative rehabilitation. NICE guideline [NG157]. 2020. www.nice.org.uk/guidance/ng157. Accessed 16.06.2020.

19. Hamilton DF, Loth FC, MacDonald DJ, MacFarlane GJ, Beard DJ, Simpson AHRW, et al. Exploring variation in patient access of post-discharge physiotherapy following total hip and knee arthroplasty under a choice based system in the UK: An observational cohort study. BMJ Open. 2019;

20. Khan F, Ng L, Gonzalez S, Hale T, Turner-Stokes L. Multidisciplinary rehabilitation programmes following joint replacement at the hip and knee in chronic arthropathy. Cochrane Database of Systematic Reviews. 2008.

21. Artz N, Elvers KT, Lowe CM, Sackley C, Jepson P, Beswick AD. Effectiveness of physiotherapy exercise following total knee replacement: Systematic review and meta-analysis. BMC Musculoskeletal Disorders. 2015.

22. Roos EM. Effectiveness and practice variation of rehabilitation after joint replacement. Current Opinion in Rheumatology. 2003.

23. Smith TO, Dainty JR, Clark EM, Whitehouse MR, Price AJ, MacGregor AJ. Demographic and geographical variability in physiotherapy provision following hip and knee replacement. An analysis from the National Joint Registry for England, Wales, Northern Ireland and the Isle of Man. Physiotherapy 2020;106:1-11. doi:10.1016/j.physio.2019.11.003

24. Buhagiar MA, Naylor JM, Harris IA, Xuan W, Adie S, Lewin A. Assessment of Outcomes of Inpatient or Clinic-Based vs Home-Based Rehabilitation After Total Knee Arthroplasty: A Systematic Review and Meta-analysis. JAMA Netw Open 2019;2:e192810. doi:10.1001/

For peer review only

For peer review only - http://bmjopen.bmj.com/site/about/guidelines.xhtml

jamanetworkopen.2019.2810

25 Beauchamp MK, Ward RE, Jette AM, Bean JF. Meaningful Change Estimates for the Late-Life Function and Disability Instrument in Older Adults. J Gerontol A Biol Sci Med Sci. 2019;74(4):556-9.

26. Hamilton DF, Beard DJ, Barker KL, Macfarlane GJ, Tuck CE, Stoddart A, Wilton T, Hutchinson JD, Murray GD, Simpson AHRW. Targeting rehabilitation to improve outcomes after total knee arthroplasty in patients at risk of poor outcomes: randomised controlled trial. BMJ 2020 Oct 13;371:m3576.

27. Barker KL, Room J, Knight R, Dutton SJ, Toye F, Leal J, Kent S, Kenealy N, Schussel MM, Collins G, Beard DJ, Price A, Underwood M, Drummond A, Cook E, Lamb SE. COmmunity-based Rehabilitation after Knee Arthroplasty: A randomised controlled trial with economic and qualitative evaluations (CORKA Trial). Health Technology Assessment: 2020; 24: 1-116

For peer review only

650
651 **Table 1 - Descriptive characteristics at baseline by treatment group**
652

	Usual care (n = 312)	Home-based rehab (n = 309)	Total (n = 621)
**Gender[a]**			
Male	125 (40.1%)	125 (40.5%)	250 (40.3%)
Female	187 (59.9%)	184 (59.5%)	371 (59.7%)
**Age (years)[b]**	70.18 (8.14)	70.67 (8.01)	70.42 (8.07)
**BMI[b]**	31.65 (4.99)	31.34 (4.48)	31.50 (4.74)
**Side of operation[a]**			
Right	169 (54.2%)	169 (54.7%)	338 (54.4%)
Left	142 (45.5%)	139 (45.0%)	281 (45.2%)
Not recorded	1 (0.3%)	1 (0.3%)	2 (0.3%)
**Knee arthroplasty type[a]**			
TKA	229 (73.4%)	231 (74.8%)	460 (74.1%)
UKA	82 (26.3%)	77 (24.9%)	159 (25.6%)
Not recorded	1 (0.3%)	1 (0.3%)	2 (0.3%)
**ASA grade[a]**			
Healthy	38 (12.2%)	55 (17.8%)	93 (15.0%)
Mild systemic disease	218 (69.9%)	202 (65.4%)	420 (67.6%)
Severe systemic disease	43 (13.8%)	44 (14.2%)	87 (14.0%)
Not recorded	13 (4.2%)	8 (2.6%)	21 (3.4%)
**Falls in the last year[a]**			
Yes	77 (24.7%)	89 (28.8%)	166 (26.7%)
No	235 (75.3%)	220 (71.2%)	455 (73.3%)
**If yes, number of falls[c]**	1 (1, 3)	2 (1, 3)	2 (1, 3)
**Previous lower limb surgery[a]**			
Yes	200 (64.1%)	189 (61.2%)	389 (62.6%)
No	112 (35.9%)	120 (38.8%)	232 (37.4%)
**Screening tool score[c]**	6 (5, 6)	6 (5, 6)	6 (5, 6)

For peer review only

**Charnley ABC[a]**			
A - single knee arthroplasty	134 (42.9%)	138 (44.7%)	272 (43.8%)
B - both knees affected	140 (44.9%)	145 (46.9%)	285 (45.9%)
C - multiple joint disease/other disability	38 (12.2%)	26 (8.4%)	64 (10.3%)
**Stairs mobility[a]**			
Normal	19 (6.1%)	19 (6.1%)	38 (6.1%)
One step at a time	34 (10.9%)	39 (12.6%)	73 (11.8%)
Down with rail	18 (5.8%)	19 (6.1%)	37 (6.0%)
Up/down with rail	225 (72.1%)	216 (69.9%)	441 (71.0%)
Unable down	2 (0.6%)	3 (1.0%)	5 (0.8%)
Unable	14 (4.5%)	12 (3.9%)	26 (4.2%)
Missing	0 (0.0%)	1 (0.3%)	1 (0.2%)
**Support mobility[a]**			
None	178 (57.1%)	178 (57.6%)	356 (57.3%)
Stick outdoors	83 (26.6%)	78 (25.2%)	161 (25.9%)
Stick always	34 (10.9%)	31 (10.0%)	65 (10.5%)
2 sticks	6 (1.9%)	7 (2.3%)	13 (2.1%)
2 crutches	5 (1.6%)	7 (2.3%)	12 (1.9%)
Walking frame	6 (1.9%)	8 (2.6%)	14 (2.3%)
**Functional comorbidity index[d]**			
0	189 (60.6%)	176 (57.0%)	365 (58.8%)
1-3	112 (35.9%)	125 (40.5%)	237 (38.2%)
4-6	8 (2.6%)	6 (1.9%)	14 (2.3%)
7+	2 (0.6%)	0 (0.0%)	2 (0.3%)
Missing	1 (0.3%)	2 (0.6%)	3 (0.5%)

653
654
655 ASA = American Society of Anaesthesiologists; BMI = body mass index; TKA = total knee arthroplasty; UKA = unicompartmental
656 knee arthroplasty.
657 [a] These values are reported as numbers and percentages
658 [b] These values are reported as means and standard deviations
659 [c] These values are reported as medians and interquartile ranges
660 [d] The functional comorbidity index (85) counts the number of comorbidities experienced by each patient, giving more weight to
661 more severe conditions. This was categorised in four groups: (i) no comorbidities (0), (ii) small number of comorbidities (1-3),
662 (iii) medium number of comorbidities (4-6), and (iv) high number of comorbidities (7+).
663

For peer review only

Table 2 - Comparison of treatment groups for secondary outcomes (intention-to-treat population using available cases)

		Usual care		Home-based rehab		Adjusted difference (95% CI)[1]	p-value
		Mean (SD)	Total	Mean (SD)	Total		
**LLFDI Disability (frequency)**	**Baseline**	51.28 (7.25)	311	51.55 (7.46)	308	-	-
	**6 months**	54.27 (6.86)	283	54.91 (6.85)	275	0.64 (-0.50, 1.78)	0.27
	**12 months**	54.13 (6.87)	287	55.05 (6.87)	279	0.93 (-0.21, 2.06)	0.11
**LLFDI Disability (limitation)**	**Baseline**	66.26 (11.96)	312	66.25 (12.48)	305	-	-
	**6 months**	72.33 (15.25)	284	74.72 (15.24)	275	2.39 (-0.14, 4.92)	0.06
	**12 months**	74.10 (15.27)	287	74.75 (15.28)	277	0.65 (-1.87, 3.18)	0.61
**OKS**	**Baseline**	20.59 (7.50)	312	20.81 (7.31)	308	-	-
	**6 months**	35.69 (7.69)	284	36.23 (7.69)	277	0.54 (-0.73, 1.81)	0.40
	**12 months**	37.34 (7.71)	287	37.80 (7.71)	278	0.46 (-0.81, 1.73)	0.48
**KOOS – QoL subscale**	**Baseline**	25.50 (16.58)	301	25.50 (15.89)	300	-	-
	**6 months**	61.26 (22.31)	273	62.47 (22.29)	269	1.21 (-2.55, 4.97)	0.53
	**12 months**	65.53 (22.37)	277	65.26 (22.36)	271	-0.27 (-4.02, 3.47)	0.89
**PASE**	**Baseline**	121.24 (73.90)	302	115.11 (72.95)	297	-	-
	**6 months**	150.62 (66.72)	259	149.41 (66.46)	245	-1.22 (-12.86, 10.42)	0.84
	**12 months**	156.58 (66.82)	263	154.63 (66.73)	251	-1.95 (-13.51, 9.61)	0.74
**EQ5D-5L Utility**	**Baseline**	0.52 (0.22)	308	0.52 (0.23)	308	-	-
	**6 months**	0.75 (0.18)	277	0.75 (0.18)	275	0.00 (-0.03, 0.03)	0.85
	**12 months**	0.76 (0.18)	282	0.76 (0.18)	278	-0.00 (-0.03, 0.03)	0.99
**EQ5D-5LVAS**	**Baseline**	68.53 (19.46)	312	68.03 (19.13)	308	-	-

For peer review only

**6 months**	75.81 (14.55)	283	75.51 (14.54)	275	-0.30 (-2.71, 2.11)	0.81
**12 months**	76.80 (14.57)	287	77.05 (14.58)	278	0.24 (-2.16, 2.65)	0.84

Notes: Totals refer to the total number of participants in each group that provided each score at each timepoint. Adjusted differences were calculated using linear mixed effects models adjusted for stratification factors and baseline scores. LLFDI = Late Life Function and Disability Instrument, each LLFDI disability scale ranges from 0 to 100 with higher scores indicating less disability. OKS = Oxford Knee Score, scores range from 0 to 48 with higher scores indicating better knee function. KOOS = Knee injury and Osteoarthritis Outcome Score, scores range from 0 to 100 with higher scores indicating better QoL. PASE = Physical Activity Scale for the Elderly, higher scores indicate greater levels of physical activity. EQ5D-5L utility scores range from -0.594 to 1 and EQ5D-5L visual analogue scale (VAS) ranges from 0 to 100, in both cases higher scores indicate better quality of life. CI = confidence interval; SD = standard deviation.

For peer review only

Table 3 - Comparison of physical measures between treatment groups at follow-up time points

	Usual care (n = 312)		Home-based rehab (n = 309)		Adjusted difference (95% CI)	p-value
	Summary	N	Summary	N		
30SCST # stands[a]						
**Baseline**	8.2 (3.5)	312	8.4 (3.7)	309	-	-
**6 months**	11.1 (3.1)	278	11.0 (3.1)	275	-0.06 (-0.548, 0.46)	0.82
**12 months**	11.7 (3.1)	279	11.5 (3.1)	276	-0.22 (-0.74, 0.3)	0.41
F8WT (seconds)[a]						
**Baseline**	11.8 (5.4)	312	12.1 (4.9)	309	-	-
**6 months**	10.2 (3.6)	277	9.6 (3.6)	275	-0.57 (-1.17, 0.04)	0.07
**12 months**	9.3 (3.6)	280	9.1 (3.6)	274	-0.17 (-0.78, 0.43)	0.58
F8WT steps[a]						
**Baseline**	16.4 (4.5)	312	17.3 (5.0)	309	-	-
**6 months**	15.5 (3.2)	277	15.2 (3.2)	275	-0.36 (-0.91, 0.18)	0.19
**12 months**	15.2 (3.2)	280	14.9 (3.3)	274	-0.23 (-0.77, 0.31)	0.40
KA leg SLS average (seconds)[a]						
**Baseline**	5.3 (2.6, 13.0)	310	4.5 (1.9, 14.5)	308	-	-
**6 months**	10.0 (3.9, 30.3)	279	10.0 (3.6, 26.8)	276	-	0.57
**12 months**	13.3 (5.9, 31.1)	280	11.7 (3.4, 31.1)	276	-	0.14
Contralateral leg SLS average[b] ***(seconds)***						
**Baseline**	7.2 (3.6, 22.0)	310	6.3 (2.6, 18.0)	308	-	-
**6 months**	10.9 (4.2, 30.8)	279	9.8 (3.5, 29.7)	276	-	0.43
**12 months**	14.7 (5.6, 30.7)	280	11.0 (3.2, 29.7)	276	-	0.03

For peer review only

Notes: Totals refer to the total number of participants in each group that provided each score at each timepoint. 30SCST = 30 Seconds Chair Stand Test, which records the number of stands in 30 seconds and where higher scores indicate better function. F8WT = Figure of 8 Walk Test, where a quicker time and fewer steps indicates better function. SLS = Single Leg Stance, in which stance time is averaged over three trials at standing on the same leg, with a maximum score of 45 seconds and higher scores indicate better function

[a] *Summaries are mean (SD)*

[b] *Summaries are median (IQR)*

For peer review only

Table 4: SOC codes of SAEs by treatment group

	Usual Care (n= 14; 4.5%)	Home-based Rehab (n= 18; 5.8%)
**Blood/lymphatic**	2	2
**Cardiac**	2	1
**Endocrine**	1	0
**Gastrointestinal**	2	2
**Immune system**	0	1
**Infections/infestations**	1	3
**Musculoskeletal**	1	6
**Nervous system**	0	1
**Renal/urinary**	2	0
**Respiratory/thoracic**	0	1
**Skin**	1	0
**Social circumstances**	1	0
**Vascular**	1	0
**Unknown**	0	1

For peer review only

Figure 1 - CONSORT flow chart

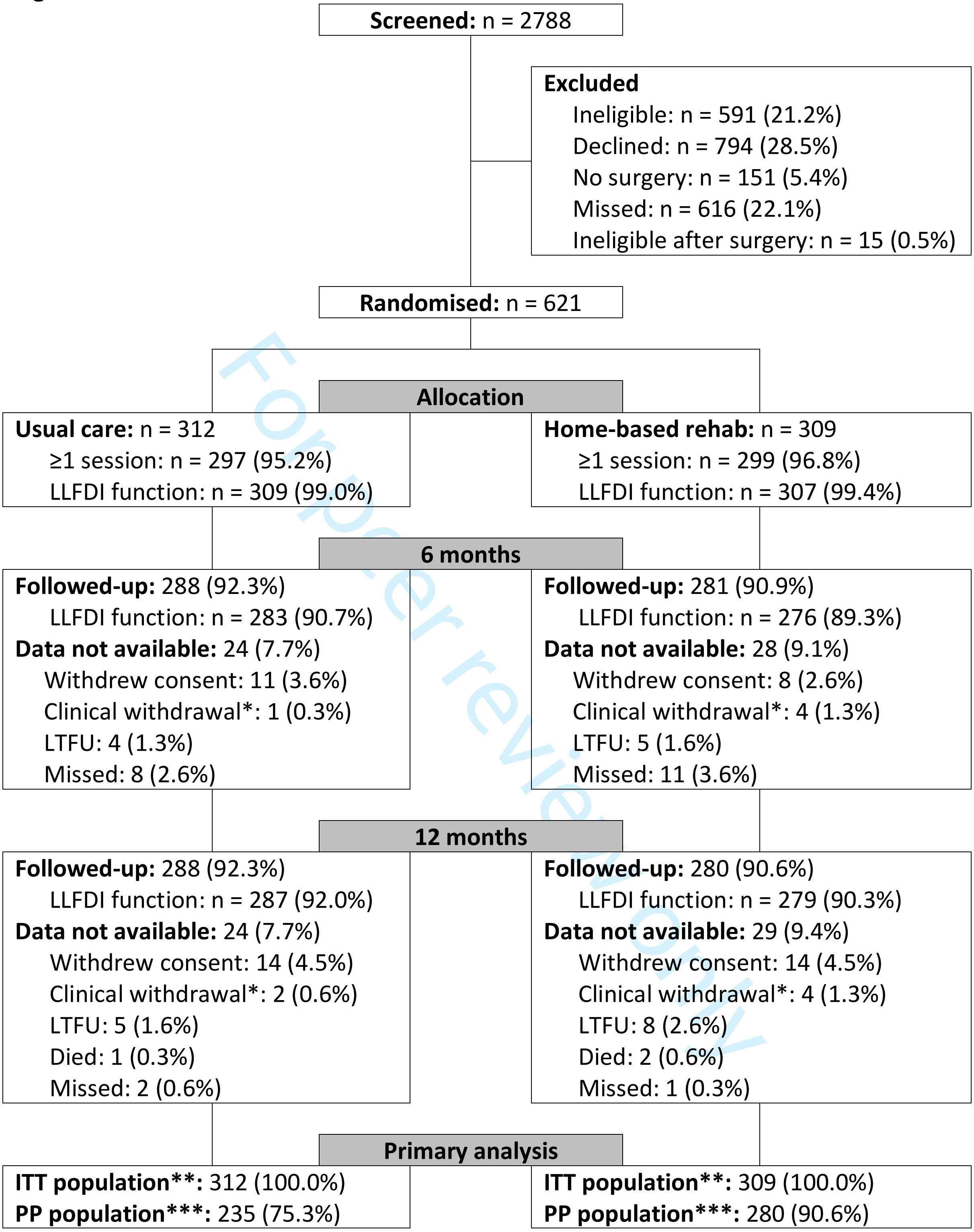

**Clinical withdrawals include those who withdrew due to an adverse event, serious adverse event, or medical contraindication*

**Missing follow-up data in the intent-to-treat (ITT) population was imputed using multiple imputation for the primary outcome analysis.*

***Analysis of the per-protocol (PP) population was based on available data only.*

LLFDI: Late Life Function and Disability Instrument; LTFU: lost to follow-up

For peer review only

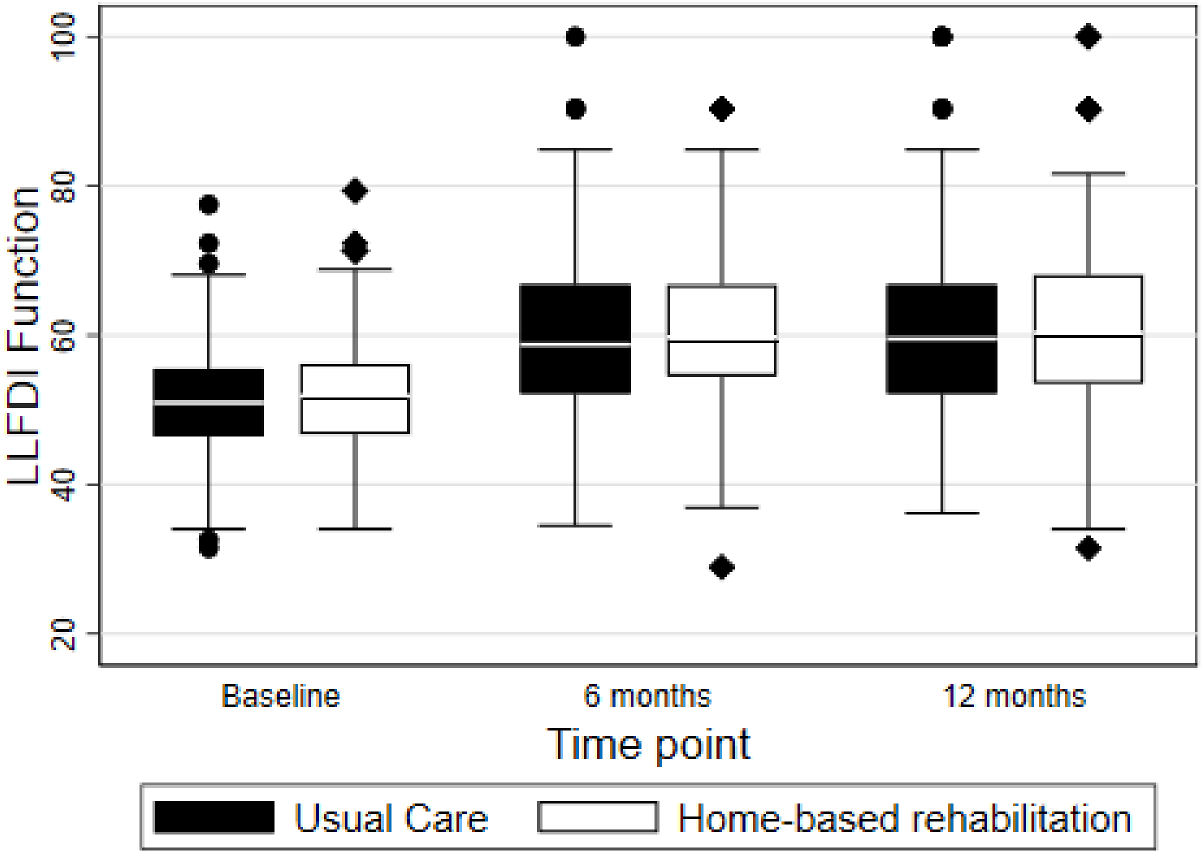

For peer review only - http://bmjopen.bmj.com/site/about/guidelines.xhtml

Corka

CORKA Screening Tool

Study code:	Site ID code:	Participants Study Number*:	Initials:
C K –	–		

* Add in once Participant has been randomised

DOB: _ _/_ _ _/_ _ _ _ **Principal Investigator:** ________________ Date _ _/_ _ _/_ _ _ _

Participant Screening Number (number given when participant originally screened) CSN __ __ __ __

From pre-admission clinic, complete the following:

	**SCORE** *(Please Circle)*
1. What is the participant's Body Mass Index (BMI)?	
Normal weight (<25kg/m^2)	**0**
Overweight (25-29.9kg/m^2)	**1**
Obese (≥30kg/m^2)	**2**
2. From Q1 of the Oxford Knee Score (OKS) questionnaire; *'How would you describe the pain you usually have from your knee?'*	
None OR very mild OR mild	**0**
Moderate OR severe	**4**
3. What is the participant's health status?	
Fit and healthy with mild systemic disease i.e. controlled hypertension/diabetes	**0**
Severe systemic disease i.e. unstable coronary heart disease	**2**
4. In the **past four weeks** have you been limited in the kind of work you do or other regular activities you carry out as a result of feeling anxious or depressed?	
None of the time OR a little of the time OR some of the time	**0**
Most of the time OR all of the time	**2**
**TOTAL:**	

For patients with a score of 5 or more, continue to screen for eligibility onto the study.

For patients with a score below 5, stop the screening process and record this in the screening log.

For peer review only - http://bmjopen.bmj.com/site/about/guidelines.xhtml

Appendix B – content of treatment Interventions

The CORKA home-based intervention was a multicomponent rehabilitation programme delivered to participants in their own home by a mixture of qualified staff and rehabilitation assistants. Its main component was an exercise programme, but it also included functional task practice, adherence approaches and provision of appropriate aids or equipment. It consisted of an initial appointment and 6 follow up sessions.

Initial appointment – assessment

The subjective assessment included their current symptoms and the perceived level of pain or discomfort on a VAS scale from 0-10 with 0 being 'no symptoms' and 10 the 'worse imaginable symptoms', their social history, any medication taken, their history of falls and their activities of daily living (ADLs).

The objective assessment documented the participant's wound, the range of movement in the operated and non-operated knees, strength of quadriceps, hamstrings, hip abductors and the calf muscles, scored on an Oxford grading scale. Standing balance was assessed by observing and recording the ability to stand with feet side by side, or in a semi-tandem or tandem stance. An assessment was made of gait and if appropriate ability to ascend and descend stairs. If deemed necessary and safe any transfers were assessed.

Finally, observation of at least one functional task took place which was identified by the participant as being potentially problematic. The exercise programme was subsequently tailored to meet the specific needs, goals and functional problems of the individual.

Initial appointment – exercise programme

Following the assessment the physiotherapist and rehabilitation assistant set each participant an exercise programme, which would replace any post-operative programme given on discharge from hospital. It would consist of exercises categorised into the following sections :

1. Knee range of motion - flexion
2. Knee range of motion – extension
3. Basic quadriceps strengthening
4. Strengthening exercises – quadriceps
5. Strengthening exercises – hamstrings
6. Strengthening exercises – hip abductors
7. Strengthening exercises – calf
8. Static balance exercises
9. Dynamic balance and walking skills exercises

The selection of each specific exercise took place as follows, for ROM exercises the physiotherapist selected an exercise and then checked that they and the participant were happy with how it was performed. If this was the case the participant was asked to perform 3 sets of 10-15 repetitions, unless a stretch had been selected, when they were asked to undertake 3 sets of 3-5 repetitions with a 30 to 60 second hold.

For strengthening exercises the participant was given an exercise that they could perform for 6-12 repetitions with good form at a moderate intensity. Perceived intensity was measured using the 10 point Rating of Perceived Exertion (RPE) scale (Borg 1998). The participant was given an exercise

For peer review only

where they feel they were working at RPE of 3-4 (moderate to somewhat hard). Participants were asked to complete 1 to 3 sets of 6-12 reps of strengthening exercises.

For the balance section, participants were prescribed an exercise that was sufficiently challenging without putting the participant at risk of falling. Participants were asked to perform 3 repetitions of the exercise with a 30 second hold.

For the final section, gait skills there were a variety of exercises to choose from. The physiotherapist was asked to prescribe an exercise that was sufficiently challenging without putting the participants at risk of falling. The exact number of sets and reps were determined based on the particular exercise selected and the individual patient.

During session 3 a graduated walking programme was introduced. Participants were asked about the maximum time or distance that they have managed to walk since surgery and were encouraged to increase this distance or time gradually in small increments.

Participants were also asked to practice between 1-3 specific functional tasks highlighted in the initial assessment.

CORKA intervention exercise prescription time points

	Range of Movement	Basic Quads strength	Strengthening	Balance	Gait Skills	Task practice	Walking programme
Session 1	✓	✓	✓	✓	✓	✓	
Session 2	✘	✘	⇧	⇧	⇧	⇧	
Session 3	✘	✘	⇧	⇧	⇧	⇧	✓
Session 4	✘	✘	⇧	⇧	⇧	⇧	⇧
Session 5	✘	✘	⇧	⇧	⇧	⇧	⇧
Session 6	✘	✘	⇧	⇧	⇧	⇧	⇧
Session 7	✘	✘	⇧	⇧	⇧	⇧	⇧

✓ = issue exercise

✘ = Stop exercise once appropriate level of function reached

⇧ = Progress exercise

For peer review only

CORKA Intervention Exercises Table

Exercise	Level	Purpose	Functional relevance
Hip and knee flexion 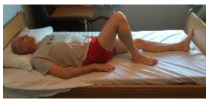	1A	Lower limb range of movement	Walking, stair climbing, kneeling
Passive knee flexion 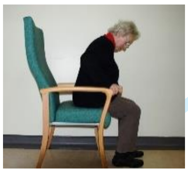	1B	Lower limb range of movement	Walking, stair climbing, sitting and standing up from a chair, kneeling
Active assisted knee flexion 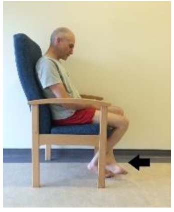	1C	Lower limb range of movement	Walking, stair climbing, sitting and standing up from a chair, kneeling
Hold-relax knee flexion 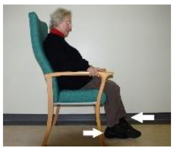	1D	Lower limb range of movement	Walking, stair climbing, sitting and standing up from a chair, kneeling
Active Knee flexion 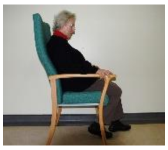	1E	Lower limb range of movement	Walking, stair climbing, sitting and standing up from a chair, kneeling
Hip and knee flexion with overpressure 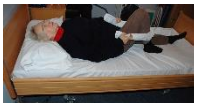	1F	Lower limb range of movement	Walking, stair climbing, kneeling
Knee extension stretch 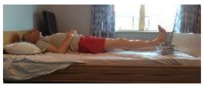	2A	Lower limb range of movement	Walking, stair climbing, standing up from a chair

Knee extension with overpressure 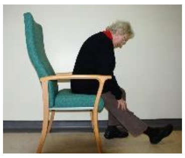	2B	Lower limb range of movement	Walking, stair climbing, standing up from a chair
Knee extension 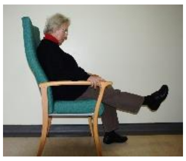	2C	Lower limb range of movement	Walking, stair climbing, standing up from a chair, dressing, getting in/out of car
Hamstring stretch 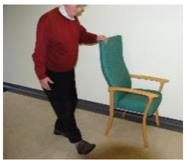	2D	Lower limb range of movement	Walking, stair climbing, dressing
Calf stretch 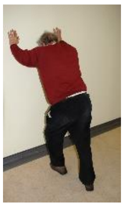	2E	Lower limb range of movement	Walking, stair climbing, dressing
Static Quadriceps 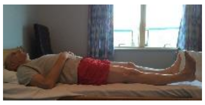	3A	Lower limb strength	Walking, stair climbing, standing up from a chair
Inner range quadriceps 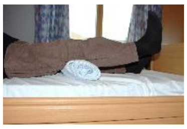	3B	Lower limb strength	Walking, stair climbing, standing up from a chair
Long arc quadriceps 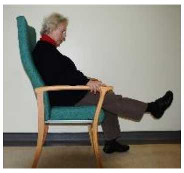	4A	Lower limb strength	Walking, stair climbing, standing up from a chair

For peer review only

Knee extension with ankle weight 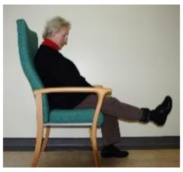	4B	Lower limb strength	Walking, stair climbing, standing up from a chair
Sit to stand using arms 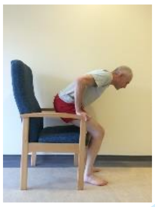	4C	Upper and lower body strength	Walking, stair climbing, standing up from a chair, lifting/carrying household objects, getting in/out of car
Sit to stand without arms 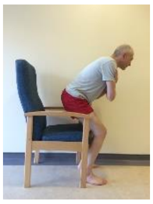	4D	Lower limb and trunk strength	Stair climbing, standing up from a chair, walking, getting in/out of car
Wall slide 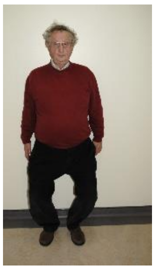	4E	Lower limb strength	Stair climbing, standing up from a chair, walking, getting in/out of car, lifting household objects
Mini Squats 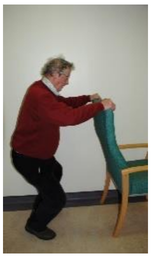	4F	Lower limb strength	Stair climbing, standing up from a chair, walking, getting in/out of car, lifting household objects

For peer review only

Single leg dip 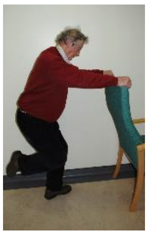	4G	Lower limb strength	Stair climbing, standing up from a chair, walking, lifting household objects
Static hamstrings 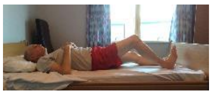	5A	Lower limb strength	Stair climbing, standing up from a chair, walking, lifting household objects
Sliding heel backwards 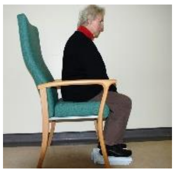	5B	Lower limb strength and range of movement	Stair climbing, standing up from a chair, walking, kneeling
Standing hamstring curls 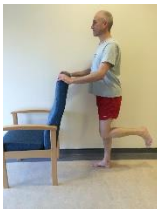	5C	Lower limb strength	Stair climbing, standing up from a chair, walking, lifting household objects
Active hamstrings 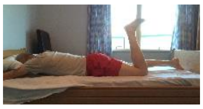	5D	Lower limb strength	Stair climbing, standing up from a chair, walking, lifting household objects
Standing hamstring curls with ankle weight 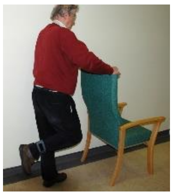	5E	Lower limb strength	Stair climbing, standing up from a chair, walking, lifting household objects
Lunge 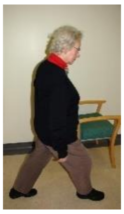	5F	Lower limb strength	Stair climbing, standing up from a chair, walking, lifting household objects

For peer review only

Hip abduction 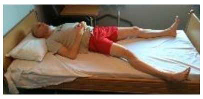	6A	Lower limb strength	Walking, dressing, getting into/out of bed
Bridging 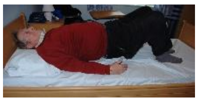	6B	Lower limb strength	Dressing, walking , standing up from a chair
Hip hitching 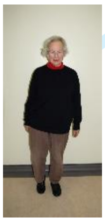	6C	Lower limb strength	Walking, dressing, getting into/out of bed
Standing hip abduction 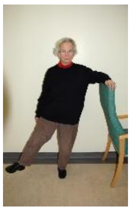	6D	Lower limb strength	Walking, dressing, getting into/out of bed, standing from a chair
Standing hip abduction with ankle weight 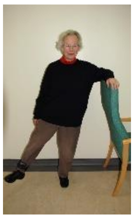	6E	Lower limb strength	Walking, dressing, getting into/out of bed, standing from a chair
Short lever hip abduction in side lying 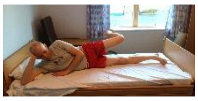	6F	Lower limb strength	Walking, dressing, getting into/out of bed
Long lever hip abduction in side lying 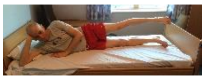	6G	Lower limb strength	Walking, dressing, getting into/out of bed
Hip abduction in side lying with ankle weight 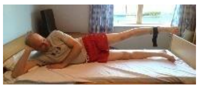	6H	Lower limb strength	Walking, dressing, getting into/out of bed

For peer review only

Bilateral calf raise 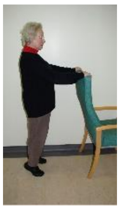	7A	Lower limb strength	Walking and stair climbing
Single calf raise 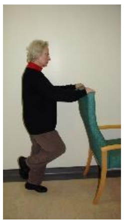	7B	Lower limb strength	Walking and stair climbing
Standing feet together 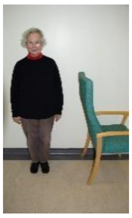	8A	Balance	Falls Prevention
Semi-tandem stance 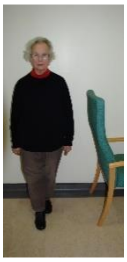	8B	Balance	Falls prevention
Tandem stance 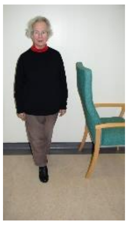	8C	Balance	Falls Prevention
Single leg stance 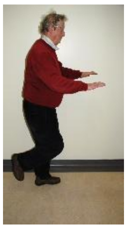	8D	Balance	Falls Prevention

For peer review only

Walking on the spot 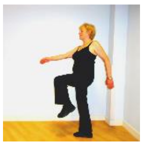	9A	Walking skills and balance	Walking and falls prevention
Waling forwards and backwards 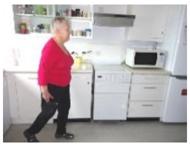	9B	Walking skills and balance	Walking a straight path and falls prevention
Side stepping 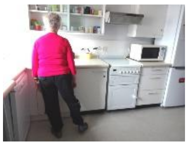	9C	Walking skills and balance	Walking, stepping around obstacles, falls prevention
Step ups 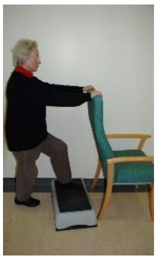	9D	Walking skills and balance	Walking, stepping onto kurbs, stair climbing, falls prevention
Mini lunge step up 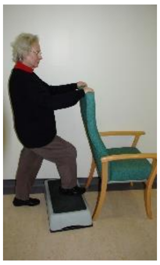	9E	Walking skills and balance	Walking, stepping onto kurbs, stair climbing, falls prevention
Turning 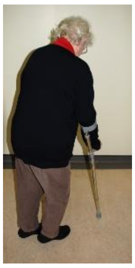	9F	Walking skills and balance	Walking with aid and falls prevention

Figure of 8 walk 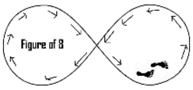	9G	Walking skills and balance	Walking both straight and curved paths, falls prevention
Tandem walking 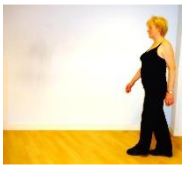	9H	Walking skills and balance	Walking and falls prevention

Usual Care

Participants randomised to usual care underwent standard in and out-patient post-operative physiotherapy as it was delivered routinely at each of the 14 research sites.

On discharge participants were given general written advice following knee arthroplasty and a home exercise programme to continue with until they were seen by an out-patient physiotherapist. To standardise usual care, participants would receive at least 1 session of physiotherapy and up to a maximum of 6 sessions which aligns with what the NHS can realistically provide. The exact duration and content of the physiotherapy sessions were at the treating physiotherapist's discretion, but in general would be between 30 and 60 minutes duration and would include a combination of range of motion and strengthening exercises, gait re-education with progression of walking aids and advice. Any differences seen in usual care across the sites is reflective of the variety of standard care delivered post knee arthroplasty across the UK.

Photographs

All participants consented to the use of their image in the treatment guide

For peer review only

CONSORT 2010 checklist of information to include when reporting a randomised trial*

Section/Topic	Item No	Checklist item	Reported on page No
**Title and abstract**			
	1a	Identification as a randomised trial in the title	1
	1b	Structured summary of trial design, methods, results, and conclusions (for specific guidance see CONSORT for abstracts)	1-2
**Introduction**			
Background and objectives	2a	Scientific background and explanation of rationale	3-4
	2b	Specific objectives or hypotheses	1 & 4
**Methods**			
Trial design	3a	Description of trial design (such as parallel, factorial) including allocation ratio	4-8
	3b	Important changes to methods after trial commencement (such as eligibility criteria), with reasons	n/a
Participants	4a	Eligibility criteria for participants	5
	4b	Settings and locations where the data were collected	6
Interventions	5	The interventions for each group with sufficient details to allow replication, including how and when they were actually administered	6
Outcomes	6a	Completely defined pre-specified primary and secondary outcome measures, including how and when they were assessed	7
	6b	Any changes to trial outcomes after the trial commenced, with reasons	n/a
Sample size	7a	How sample size was determined	7
	7b	When applicable, explanation of any interim analyses and stopping guidelines	n/a
Randomisation:			
Sequence generation	8a	Method used to generate the random allocation sequence	5
	8b	Type of randomisation; details of any restriction (such as blocking and block size)	5
Allocation concealment mechanism	9	Mechanism used to implement the random allocation sequence (such as sequentially numbered containers), describing any steps taken to conceal the sequence until interventions were assigned	5
Implementation	10	Who generated the random allocation sequence, who enrolled participants, and who assigned participants to interventions	5
Blinding	11a	If done, who was blinded after assignment to interventions (for example, participants, care providers, those	5

For peer review only

For peer review only - http://bmjopen.bmj.com/site/about/guidelines.xhtml

		assessing outcomes) and how	
	11b	If relevant, description of the similarity of interventions	5-6
Statistical methods	12a	Statistical methods used to compare groups for primary and secondary outcomes	6-8
	12b	Methods for additional analyses, such as subgroup analyses and adjusted analyses	6-8
**Results**			
Participant flow (a diagram is strongly recommended)	13a	For each group, the numbers of participants who were randomly assigned, received intended treatment, and were analysed for the primary outcome	9,22
	13b	For each group, losses and exclusions after randomisation, together with reasons	9,22
Recruitment	14a	Dates defining the periods of recruitment and follow-up	6
	14b	Why the trial ended or was stopped	n/a
Baseline data	15	A table showing baseline demographic and clinical characteristics for each group	Table 1
Numbers analysed	16	For each group, number of participants (denominator) included in each analysis and whether the analysis was by original assigned groups	9 & 24
Outcomes and estimation	17a	For each primary and secondary outcome, results for each group, and the estimated effect size and its precision (such as 95% confidence interval)	27-29
	17b	For binary outcomes, presentation of both absolute and relative effect sizes is recommended	n/a
Ancillary analyses	18	Results of any other analyses performed, including subgroup analyses and adjusted analyses, distinguishing pre-specified from exploratory	9
Harms	19	All important harms or unintended effects in each group (for specific guidance see CONSORT for harms)	11,30
**Discussion**			
Limitations	20	Trial limitations, addressing sources of potential bias, imprecision, and, if relevant, multiplicity of analyses	12
Generalisability	21	Generalisability (external validity, applicability) of the trial findings	11-13
Interpretation	22	Interpretation consistent with results, balancing benefits and harms, and considering other relevant evidence	11-13
**Other information**			
Registration	23	Registration number and name of trial registry	2
Protocol	24	Where the full trial protocol can be accessed, if available	4
Funding	25	Sources of funding and other support (such as supply of drugs), role of funders	2

*We strongly recommend reading this statement in conjunction with the CONSORT 2010 Explanation and Elaboration for important clarifications on all the items. If relevant, we also recommend reading CONSORT extensions for cluster randomised trials, non-inferiority and equivalence trials, non-pharmacological treatments, herbal interventions, and pragmatic trials. Additional extensions are forthcoming: for those and for up to date references relevant to this checklist, see www.consort-statement.org.

For peer review only - http://bmjopen.bmj.com/site/about/guidelines.xhtml

TIDieR
Template for Intervention Description and Replication

The TIDieR (Template for Intervention Description and Replication) Checklist*:

Information to include when describing an intervention and the location of the information

Item number	Item	Where located ** Primary paper (page or appendix number)	Other † (details)
	**BRIEF NAME**		
**1.**	Provide the name or a phrase that describes the intervention.	P5 lines 126-136	Paper Ref 13
	**WHY**		
**2.**	Describe any rationale, theory, or goal of the elements essential to the intervention.	_P3 l83-89	_Ref 13 & 22_
	**WHAT**		
**3.**	Materials: Describe any physical or informational materials used in the intervention, including those provided to participants or used in intervention delivery or in training of intervention providers. Provide information on where the materials can be accessed (e.g. online appendix, URL).	______________	_Ref 13 & 22_
**4.**	Procedures: Describe each of the procedures, activities, and/or processes used in the intervention, including any enabling or support activities.	______________	__Ref 13 & 22_
	**WHO PROVIDED**		
**5.**	For each category of intervention provider (e.g. psychologist, nursing assistant), describe their expertise, background and any specific training given.	_P5 l 135-136_	______________
	**HOW**		
**6.**	Describe the modes of delivery (e.g. face-to-face or by some other mechanism, such as internet or telephone) of the intervention and whether it was provided individually or in a group.	_p5 l 126-146__	______________
	**WHERE**		
**7.**	Describe the type(s) of location(s) where the intervention occurred, including any necessary infrastructure or relevant features.	__p5 126-146_	______________

For peer review only - http://bmjopen.bmj.com/site/about/guidelines.xhtml

	**WHEN and HOW MUCH**		
**8.**	Describe the number of times the intervention was delivered and over what period of time including the number of sessions, their schedule, and their duration, intensity or dose.	_p5 l133-135	______________
	**TAILORING**		
**9.**	If the intervention was planned to be personalised, titrated or adapted, then describe what, why, when, and how.	______________	__Ref 13 & 22_
	**MODIFICATIONS**		
**10.‡**	If the intervention was modified during the course of the study, describe the changes (what, why, when, and how).	________n/a___	______________
	**HOW WELL**		
**11.**	Planned: If intervention adherence or fidelity was assessed, describe how and by whom, and if any strategies were used to maintain or improve fidelity, describe them.	______________	_Ref 11 & 22_
**12.‡**	Actual: If intervention adherence or fidelity was assessed, describe the extent to which the intervention was delivered as planned.	______________	_Ref 22

** **Authors** - use N/A if an item is not applicable for the intervention being described. **Reviewers** – use '?' if information about the element is not reported/not sufficiently reported.

† If the information is not provided in the primary paper, give details of where this information is available. This may include locations such as a published protocol or other published papers (provide citation details) or a website (provide the URL).

‡ If completing the TIDieR checklist for a protocol, these items are not relevant to the protocol and cannot be described until the study is complete.

* We strongly recommend using this checklist in conjunction with the TIDieR guide (see *BMJ* 2014;348:g1687) which contains an explanation and elaboration for each item.

* The focus of TIDieR is on reporting details of the intervention elements (and where relevant, comparison elements) of a study. Other elements and methodological features of studies are covered by other reporting statements and checklists and have not been duplicated as part of the TIDieR checklist. When a **randomised trial** is being reported, the TIDieR checklist should be used in conjunction with the CONSORT statement (see www.consort-statement.org) as an extension of **Item 5 of the CONSORT 2010 Statement.** When a **clinical trial protocol** is being reported, the TIDieR checklist should be used in conjunction with the SPIRIT statement as an extension of **Item 11 of the SPIRIT 2013 Statement** (see www.spirit-statement.org). For alternate study designs, TIDieR can be used in conjunction with the appropriate checklist for that study design (see www.equator-network.org).

For peer review only - http://bmjopen.bmj.com/site/about/guidelines.xhtml
